# Land vertebrates increasingly exposed to multiple extreme events by 2085

**DOI:** 10.1038/s41559-026-03050-0

**Published:** 2026-04-24

**Authors:** Stefanie Heinicke, Karim Zantout, Hjalmar S. Kühl, Christopher P. O. Reyer, Sandra Zimmermann, Maik Billing, Simon N. Gosling, Manolis Grillakis, Stijn Hantson, Akihiko Ito, Sian Kou-Giesbrecht, Aristeidis Koutroulis, Benedikt Mester, Hannes Müller Schmied, Sebastian Ostberg, Kedar Otta, Yadu Pokhrel, Katja Frieler

**Affiliations:** 1 Potsdam Institute for Climate Impact Research (PIK), Leibniz Association, Potsdam, Germany; 2 Faculty of Information Management and Media, Karlsruhe University of Applied Sciences, Karlsruhe, Germany; 3 Senckenberg Natural History Museum Görlitz, Görlitz, Germany; 4 International Institute Zittau, Dresden University of Technology, Zittau, Germany; 5 Institute for Environmental Science and Geography, University of Potsdam, Potsdam, Germany; 6 School of Geography, University of Nottingham, Nottingham, UK; 7 School of Chemical and Environmental Engineering, Technical University of Crete, Chania, Greece; 8 Facultad de Ciencias Naturales, Universidad del Rosario, Bogotá, Colombia; 9 Graduate School of Agricultural and Life Sciences, The University of Tokyo, Tokyo, Japan; 10 School of Resource and Environmental Management, Simon Fraser University, Burnaby, Canada; 11 Institute of Physical Geography, Johann Wolfgang Goethe University Frankfurt, Frankfurt am Main, Germany; 12 Senckenberg Leibniz Biodiversity and Climate Research Centre (SBiK-F), Frankfurt am Main, Germany; 13 National Institute for Environmental Studies, Tsukuba, Japan; 14 Department of Civil and Environmental Engineering, Michigan State University, East Lansing, MI USA; 15 Present Address: Swiss Re, Munich, Germany

**Keywords:** Climate-change ecology, Biodiversity

## Abstract

Understanding how species are exposed to different types of extreme events is an emerging priority to inform biodiversity conservation under climate change. Using climate impact projections and species range data, we predict changes in exposure to droughts, heatwaves, river floods and wildfires for 33,936 terrestrial vertebrate species and 794 ecoregions. By 2050, under a medium–high emission scenario (SSP3–7.0), on average 74% of the area within species’ current geographic ranges are projected to be exposed to heatwaves, 16% to wildfires, 8% to droughts and 3% to river floods. These trends include species-rich areas in the Amazon basin, Africa and Southeast Asia. By 2050, 22 ecoregions, primarily in mid-latitudes, are estimated to have at least 50% of their area exposed to two or more types of extreme events, increasing to 236 ecoregions by 2085 (SSP3–7.0). By 2085, 36% of the area within species’ ranges are projected to be exposed to multiple event types (SSP3–7.0). These findings highlight the need for further research into species’ sensitivity and adaptive capacity to extreme events, and for conservation strategies that address the impacts of multiple extreme events.

## Abstract (web summary)

There is growing evidence that extreme events linked to anthropogenic climate change can have important effects on biodiversity. Here the authors assess the exposure of terrestrial vertebrates to multiple types of extreme climate events globally.

## Main

Extreme climate events, such as heatwaves and wildfires, can have devastating impacts on terrestrial biodiversity. For example, the 2019/2020 heatwave in Australia killed more than 72,000 flying foxes^1^. In that same year, wildfires in the Pantanal killed an estimated 17 million vertebrates^2^. A review of 519 studies found that 57% of studies documented negative responses of species to extreme events, including 100 cases with a population decline of more than 25% and 31 records of local extirpations^3^.

Climate change has led to an increase in extreme climate events—a trend that is projected to intensify^4^. Vulnerability to climate change depends on three conditions: the degree to which species are exposed, their sensitivity to change and their ability to adapt^5^. One key reason extreme events are rarely integrated into conservation planning and species risk assessments is our limited understanding of the types of events to which species will be exposed most strongly across different geographic regions^6^. As a first step, we focus here on exposure, examining how species’ exposure to different types of extreme events and to multiple hazards is projected to change.

Most studies on species’ exposure to climate extremes have focused on events that can be derived directly from the output of global climate models, especially temperature extremes^7,8^ (but see ref. ^9^). However, published studies demonstrate that species’ sensitivity varies by event type, with different extremes affecting body condition, reproductive success and survival through distinct mechanisms (Table 1). In addition, the impact can be amplified when extreme events are compounded spatially and/or temporally^10^. Biodiversity research rarely incorporates output from climate impact models, that is, process-based models that translate climate data into climate impacts such as wildfires or floods (Methods). Yet, estimates of burned area from impact models, for example, are more relevant to species risks than fire weather indices derived from climate models alone. Investigating exposure to various types of extreme events can thus help identify which changes are most relevant for terrestrial biodiversity, and inform effective conservation planning.

**Table 1:** Documented examples of species’ sensitivity to different extreme event types for terrestrial vertebrates (all references in Supplementary Table 9)

Extreme event	Amphibian	Bird	Mammal	Reptile
Heatwave	Positive: • reduced infection with heat sensitive chytrid fungi Negative: • thermal stress • increased evaporative water loss • reduction in diversity of gut and skin microbiome • reduced survival of tadpoles; decline in fecundity	Negative: • dehydration and hyperthermia, leading to decline in physical condition or mortality • nest abandonment; decline in sperm quality and reproductive success • population decline • reduction in foraging activity and foraging closer to water sources	Negative: • heat stress and dehydration, leading to mortality • abandonment of offspring • decline in body condition and fecundity • population decline • alteration of activity patterns, reduction in foraging time	Ambiguous: • change in female:male ratio of embryos Negative: • reduced survival of eggs, hatchlings and adults • oxidative stress • lower growth rate
Wildfire	Positive: • habitat creation for species adapted to disturbance Negative: • injury and death by flames, smoke and heat • increase in predation risk after fire • reduction in water quality leading to slower development and smaller size of tadpoles • increased breeding dispersal • decrease in body growth	Positive: • creation of new habitat for species using dead trees, for example, for nesting cavities Negative: • injury and death by flames, smoke and heat • smoke leading to reduced body mass and activity • disruption of migration, for example due to dense smoke • loss of nesting sites, for example, tree collapse	Positive: • reduced predation in burned areas • reduction in vegetation cover and increase in insects can improve foraging Negative: • injury and death by flames, smoke and heat • change in foraging behaviour and increase in predation risk • reduction in food availability • decline in body condition • decline in fecundity	Positive: • reduction in vegetation cover can benefit species preferring open habitat • increase in introduced species Negative: • injury and death by flames, smoke and heat • reduction in food sources and shelter leading to lower survival • habitat fragmentation hindering dispersal • destruction of eggs and offspring reducing reproductive success
Drought	Positive: • increased persistence and colonization rates for certain species • improved body condition Negative: • increased susceptibility to fungal infection • reduced body condition • loss of breeding sites • decline in larval density • reduction in fecundity and survival, leading to population decline • (temporal) decline in no. of occupied sites	Positive: • in wetter regions, increased nesting success under drought • increase in abundance Ambiguous: • distribute to other areas to avoid poor conditions Negative: • (temporal) reduction in habitat for wetland birds • decline in nest success • increased mortality • population decline • increased use of artificial water sources can lead to spread of lethal disease	Positive: • population increase Ambiguous: • move to more suitable areas Negative: • decline in body condition • decline in reproductive success • increased mortality • (temporal) population decline	Positive: • increase in abundance Ambiguous: • change in species assemblages Negative: • reduced prey availability • reduction in number of offspring • reduced body condition • decline in abundance • increased mortality and local (temporal) extirpation
River flood	Negative: • habitat destruction, especially species that live underground • increased mortality through higher predation or sometimes drowning • disruption of breeding due to mortality of eggs and tadpoles, leading to population declines	Positive: • population increase and increase in fecundity, for example, through creation of new (temporary) habitats (for example, wetlands, marshes) and feeding opportunities (aquatic prey) Negative: • decrease in body condition and fecundity • destruction of nests leading to loss of eggs and nestlings • local extirpation and population decline	Positive: • population increase for aquatic species, for example due to higher prey abundance Negative: • injury and drowning • displacement leading to stress and crowding in refuge areas resulting in higher competition and predation • increase prevalence of water-borne diseases, for example, leptospirosis • local extirpation and population decline	Positive: • increase in prey availability, for example, arthropods Negative: • mortality • reduced prey availability, for example, mammal • displacement • habitat destruction

We analysed projected changes in extreme event exposure globally for terrestrial vertebrates, that is, amphibians, birds, mammals and reptiles, using a dataset of four extreme climate events (drought, heatwave, river flood and wildfire; for event definition see Methods). We used climate impact simulations from the Inter-Sectoral Impact Model Intercomparison Project (ISIMIP Phase 3b^11^), which provides output from six climate impact models (Supplementary Table 1) forced by climate projections from five climate models of the Coupled Model Intercomparison Project (CMIP6) under three emission scenarios: low (SSP1–2.6), medium–high (SSP3–7.0) and high (SSP5–8.5). We defined extreme events relative to their historical distribution within each grid cell (at a resolution of 0.5°), thereby accounting for natural spatial variations. We analysed changes in extreme event exposure between a baseline period centred on 2000 (1985–2014) and future periods, using 30-year moving windows centred between 2030 and 2085. For each grid cell, we calculated the event frequency as the proportion of years with an event within the 30-year window. We also identified grid cells with high frequencies of multiple extreme events (≥0.33 year^−1^, corresponding to events every third year) and summed the number of different event types per cell.

**Fig. 1:**
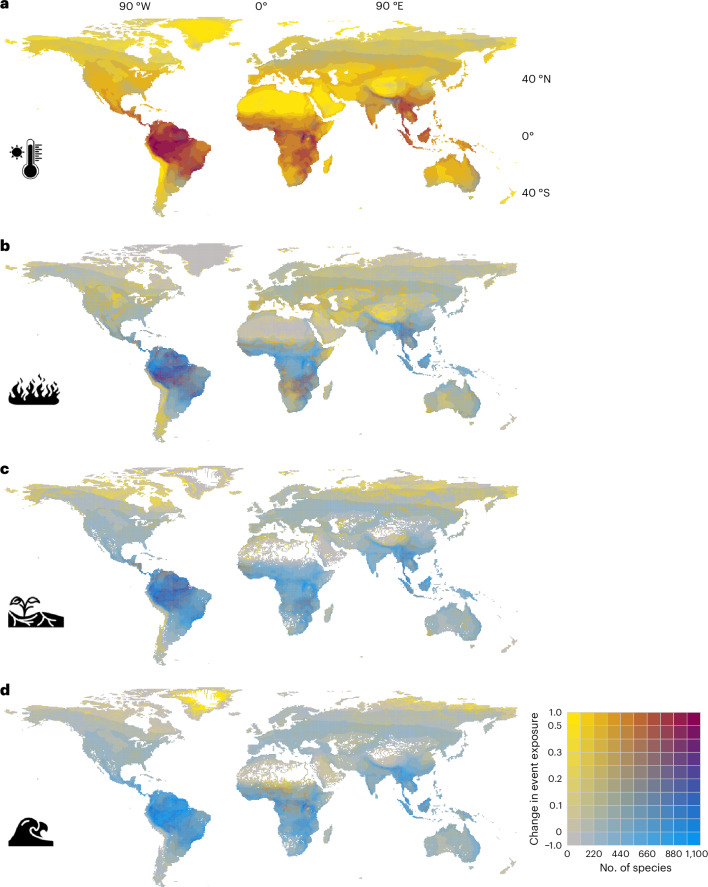
Exposure of species richness to projected change in extreme event occurrences. **a**–**d**, change in annual frequency of extreme events from 2000 to 2050 for SSP3–7.0 for heatwave (**a**), wildfire (**b**), drought (**c**) and river flood (**d**) for all four taxa combined based on species richness data from the IUCN Red List of Threatened Species. Icons from the Noun Project. Basemaps reproduced from ISIMIP Repository under a CC0 1.0 Universal Public Domain license; Lange, S., Büchner, M. 10.48364/ISIMIP.822294.

**Extended Data Fig. 1:**
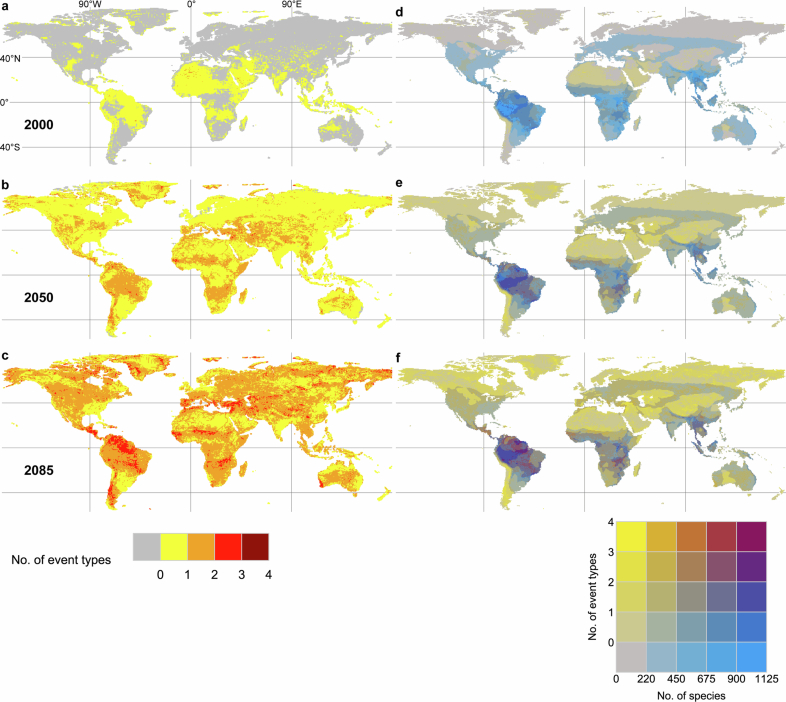
Exposure of species richness to multiple event types. **a-c**, number of event types projected for SSP3 − 7.0 for the year **(a)** 2000, **(b)** 2050 and **(c)** 2085, **d-f**, exposure to multiple events mapped against species richness combined across all four taxa (from the IUCN Red List of Threatened Species) for the same three time periods.

To identify hotspots of terrestrial biodiversity exposure, we used species richness (all species (Fig. 1 and Extended Data Fig. 1d–f) and threatened species (Supplementary Fig. 1)) and rarity-weighted richness (Supplementary Fig. 2) data from the International Union for Conservation of Nature (IUCN) Red List of Threatened Species^12^. To quantify exposure for individual species, we used species range layers from the IUCN Red List of Threatened Species^12^, the Global Assessment of Reptile Distributions^13^ and BirdLife International^14^. Due to the spatial resolution, extreme event data are not available for small islands. Species restricted to small islands not represented in the ISIMIP grid were excluded resulting in a final dataset of 33,936 terrestrial vertebrate species (7,605 amphibians; 10,562 birds; 5,476 mammals and 10,293 reptiles). For each year, we quantified species exposure as the proportion of grid cells where extreme events occur, weighted by event frequency, grid cell area and the proportion of overlap between each cell and the species’ range. Using the same approach we also quantified exposure for 794 ecoregions^15^.

## Results

### Exposure to single event types

**Fig. 2:**
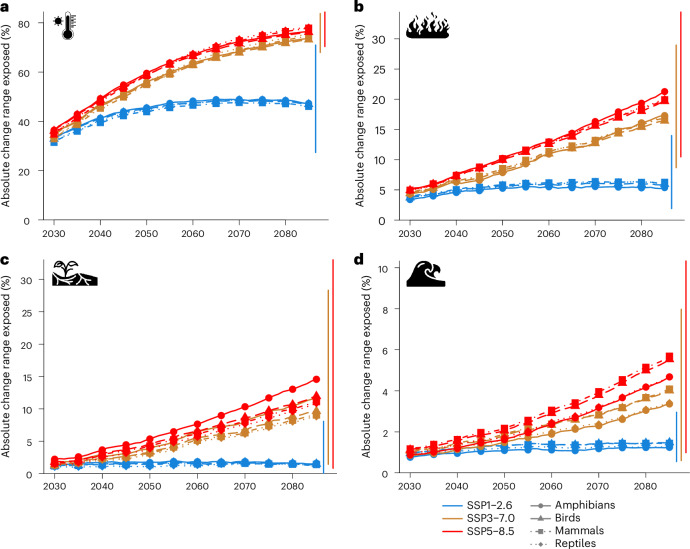
Exposure of terrestrial vertebrates to projected change in extreme event occurrences. **a**–**d**, Change in proportion of geographic range exposed relative to year 2000 averaged for each taxon for three scenarios for heatwave (**a**), wildfire (**b**), drought (**c**) and river flood (**d**). Vertical lines show the range of model ensemble across all four taxa for the year 2085. Values for each taxon and each climate model–impact model combination are in Supplementary Figs. 13–16. Icons from the Noun Project.

**Extended Data Fig. 2:**
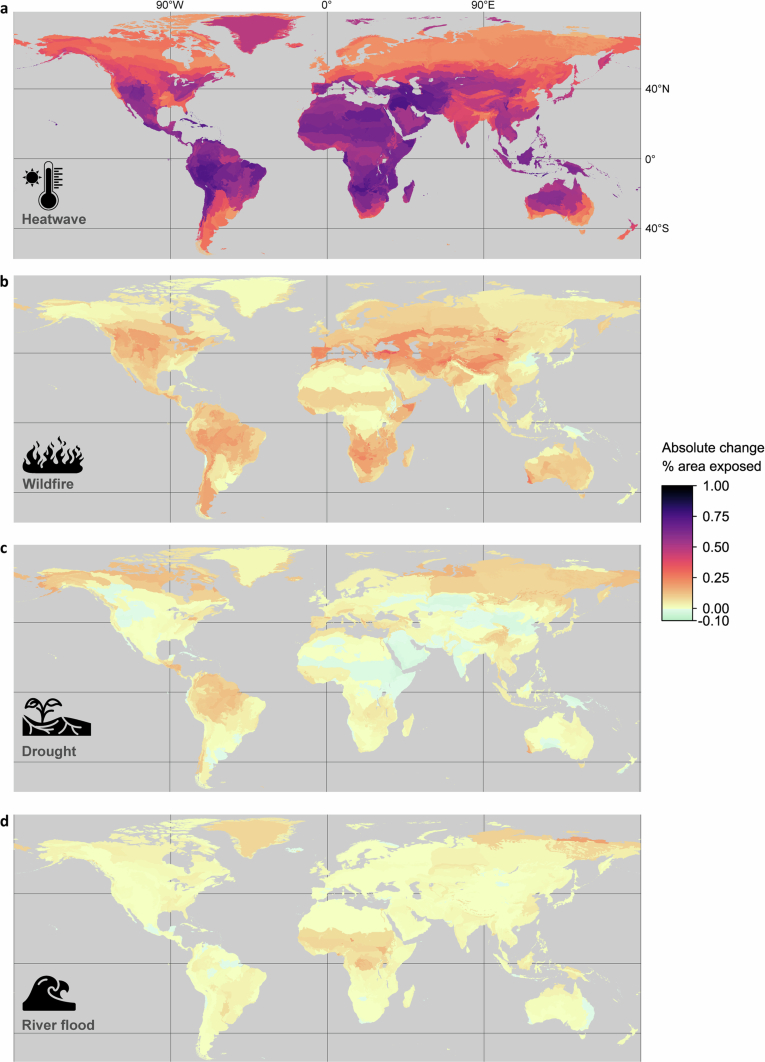
Exposure to projected change in extreme event occurrences for ecoregions. Change in frequency of extreme events from 2000 to 2050 for SSP3 − 7.0 for (**a**) heatwave, (**b**) wildfire, (**c**) drought, (**d**) river flood for 794 ecoregions. Values for each climate model – impact model combination are in Supplementary Fig. 17.

We found the strongest increase in extreme event exposure for heatwaves (Fig. 1). Under SSP1–2.6, aligned with the Paris Agreement of staying below 2° warming, on average 63% (model ensemble minimum to maximum: 45–81%; Methods) of the area within species’ ranges are projected to be exposed to heatwaves by 2050 (Supplementary Table 2). This corresponds to an increase by 45% (31–64%) compared with 2000 levels (Fig. 2a). Under SSP3–7.0, matching current developments of regional rivalry and high emissions more closely, 74% (59–90%) of the area within species’ ranges are projected to be exposed, corresponding to an increase by 56% (45–73%, Supplementary Table 2). By 2050, 9,434 (6,864–10,296) bird, 4,729 (3,302–5,299) mammal, 6,849 (5,089–7,384) amphibian and 9,155 (6,676–9,886) reptile species are projected to have at least 50% of their geographic range exposed to heatwaves (SSP3–7.0, Supplementary Tables 3–6). By 2085, 93% (85–99%) of the area within species’ ranges will be exposed to heatwaves for SSP3–7.0. Strong increases in event frequency are not only projected for species-rich areas (that is, Amazon basin, tropical Africa and Southeast Asia, Fig. 1a), but exposure increases by 50% (41–71%) across all ecoregions (SSP3–7.0; Extended Data Fig. 2a and Supplementary Table 7). Southeast Asia further emerges as a hotspot for threatened species exposure (Supplementary Fig. 1).

Extreme wildfires are projected to be the second most prevalent event. By 2050, 16% (11–22%) of the area within species’ ranges are projected to be exposed for SSP3–7.0, reaching 25% (17–35%) by 2085 (Supplementary Table 2). Hotspots for increased wildfire frequency in species-rich areas are the Amazon basin, southern Africa and Southeast Asia (Fig. 1b). Mid-latitude ecoregions will be increasingly exposed, with 130 ecoregions projected to have at least 25% of the area exposed to wildfires by 2050 (SSP3–7.0; Extended Data Fig. 2).

Drought exposure, as defined in this study, remains relatively low: 8% (3–15%) of the area within species’ ranges is projected to be exposed in 2050 (SSP3–7.0), increasing to 14% (5–29%) by 2085 (Fig. 1c and Supplementary Table 2). Amphibian ranges will be exposed more strongly compared with other taxa, especially by 2085 and for SSP3–7.0 and SSP5–8.5 (Fig. 2c and Supplementary Fig. 3). Strong increases in flood exposure are projected only for localized areas in Central Africa, taiga and tundra, especially by 2085, and for SSP5–8.5, affecting small fractions of species’ ranges (Fig. 2d). By 2050, 3% (2–5%) of the area within species’ ranges is projected to be exposed to river floods, increasing to 5% (2–10%) by 2085 (SSP3–7.0; Supplementary Table 2).

### Exposure to multiple event types

**Fig. 3:**
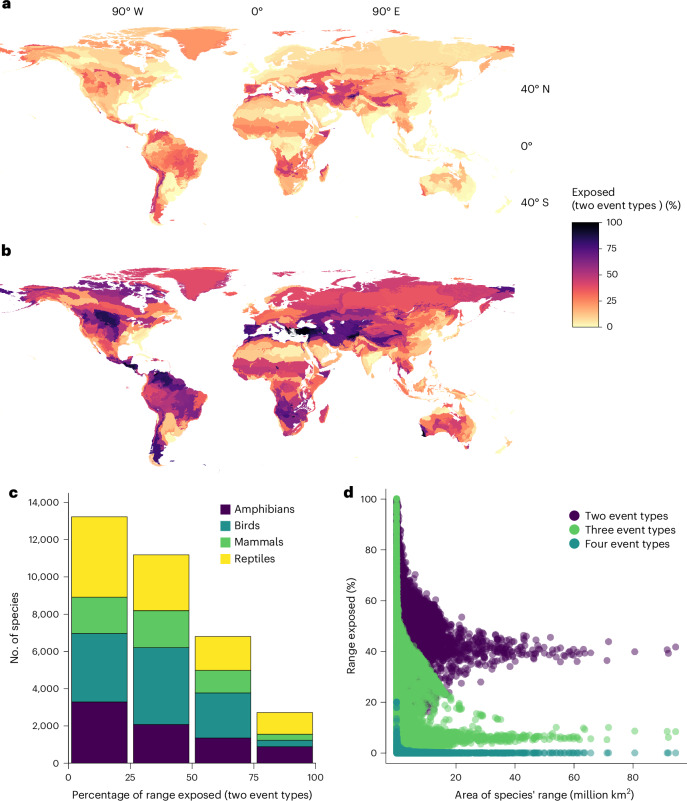
Exposure to multiple extreme events. **a**,**b**, Proportion of ecoregion projected to be exposed to at least two types of extreme events for SSP3–7.0 for the years 2050 (**a**) and 2085 (**b**). **c**, Proportion of geographic range exposed to at least two different types of extreme events for SSP3–7.0 for the year 2085. **d**, Proportion of range exposed against area of species range for different numbers of extreme event type for each species. Sensitivity of results for applying different thresholds to define multiple events are shown in Supplementary Figs. 10 and 11. Basemaps in **a** and **b** from the ISIMIP Repository under a CC0 1.0 license (10.48364/ISIMIP.822294).

By 2050, 14% (10–18%) of the area within species’ ranges are projected to be exposed to at least two types of extreme events, increasing to 36% (26–45%) by 2085 (SSP3–7.0; Supplementary Table 2, breakdown by event type combination in Supplementary Table 8 and Supplementary Fig. 4), suggesting that terrestrial vertebrates could be exposed to different types of extreme events in the same year or in consecutive years (Fig. 3 and Extended Data Fig. 1). However, for 22 ecoregions, more than 50% of the area is exposed to at least two types of events already by 2050 (SSP3–7.0; Supplementary Table 7), with these ecoregions visible in mid-latitude regions in Fig. 3a. By 2085, this increases to 236 ecoregions (Fig. 3b). For SSP1–2.6, 9% (5–12%) of the area within species’ ranges is projected to be exposed to multiple events by 2085, but this increases to 44% (36–54%) under SSP5–8.5 (Supplementary Table 2).

## Discussion

Three key considerations are necessary to contextualize our findings. First, although extreme events can impact biodiversity negatively, they can also benefit certain species^10^ (Table 1). For example, the ornate chorus frog (*Pseudacris ornate*) experiences lower predation pressure during droughts^16^. Second, some species and ecosystems are adapted to, or even rely on, these disturbances. For example, the riffian skink (*Chalcides colosii*) was found only in forested areas in early postfire years, because it depends on open habitat^17^. As a result, regions with regular flooding or moderate-intensity wildfires often exhibit greater biodiversity^17^. Third, species exhibit behavioural and physiological plasticity to adapt to changing conditions. For example, gorillas (*Gorilla beringei*) have been shown to drink more frequently with increasing maximum daily temperatures^18^. However, adaptive behaviours may involve trade-offs. For example, although high temperatures can reduce risk of fungal infections in amphibians because these fungi grow more slowly, they do not actively seek out hotter areas, probably to avoid desiccation^19^.

However, climate change is intensifying extreme events beyond what many species are likely to be able to adapt to, especially within a short timeframe^20^. Species with restricted ranges face particularly severe risks^21^, as demonstrated by the Carnaby’s Black Cockatoo (*Calyptorhynchus latirostris*), which experienced a 60% population decline following the 2011 Western Australian heatwave^22^. Meta-analyses indicate native species typically show higher vulnerability to these events than non-native species^23^. Extreme events can also cause abrupt, widespread impacts across taxonomic groups simultaneously—the 2011 Australian heatwave not only led to a crash of the Black Cockatoo population, but also caused high shrub and tree mortality, altering the habitat quality for many species^22^. Thus, when extreme events exacerbate other stressors like habitat loss and disease, they create synergistic threats to biodiversity^24^. This compounding effect was shown in a study on the 2019–2020 Australian fire showing 27–40% greater declines across plant and animal species when fire was preceded by drought, highlighting the danger of multi-hazard extreme events^25^.

Although our analysis faces limitations from uncertainties in scenarios, data and modelling, and the coarse spatial and temporal resolution, it is a first step to integrate biodiversity data with harmonized multi-hazard data. The absence of small islands in our analysis may lead to an underestimation of exposure for island-endemic species, which are particularly vulnerable given their limited range size and dispersal options. We use current species distributions with known constraints^26^, without accounting for species range shifts in response to habitat destruction or climate change. Evidence shows that range shifts often follow complex, inconsistent patterns influenced by factors beyond climate alone^27,28^, making reliable predictions challenging across all terrestrial vertebrates. Our projections show substantial increases in extreme event exposure within relatively short timeframes (by 2050), during which many species will probably shift their ranges only partially. This temporal mismatch between rapid climate change and slower biological adaptation underscores the urgency of conservation interventions.

We quantify exposure to locally extreme conditions. Whether this exposure translates into impacts depends on population-level sensitivity and adaptive capacity^5^. Although there are studies that use a species-specific threshold to define an extreme event and thereby already include species sensitivity in their threshold definition, this implies a focus on a single mechanism of sensitivity (for example, exceedance of thermal tolerance limits^29,30^). Moreover, using a fixed sensitivity threshold implies a binary classification of impact (that is, within or outside thermal tolerance), whereas extreme event impact can be gradual (for example, by changing foraging activity or altering reproductive success well within physiological tolerance limits). Furthermore, thermal tolerance can vary substantially within species across their geographic range due to local adaptation, making it difficult to define a single biologically meaningful threshold applicable across an entire species’ distribution^31,32^. Our grid-based approach focuses on exposure only, but does not assume a single mechanism of sensitivity and instead captures exposure to conditions that can impact species through multiple pathways, such as body condition, behavioural changes or resource availability (Table 1) and allows for a consistent assessment across different event types. Therefore, our estimates are not directly comparable with studies that define extremes using species-specific physiological thresholds (for example, ref. ^29^).

Because studies differ in methodology (for example, extreme event definition, input data, spatial scale, species subsets), there are differences in projected exposure magnitude and spatial patterns. For example, our results show lower levels of projected drought exposure than other studies^8^, due to differences in species subsets and whether droughts were defined using climate data (for example, precipitation) or climate impact data (for example, soil moisture). Yet, consistent with previous work, we find that amphibians are likely to experience stronger drought exposure than other taxonomic groups^7,33^. Studies consistently show that species are likely to face increasing exposure to extreme events.

Our spatially explicit projections provide a baseline of exposure to extreme events across terrestrial vertebrates and highlight the need for finer-scale research on population-level sensitivity and adaptive capacity^34^, because knowledge gaps persist even for well-studied groups like great apes^35^. Research agendas on species’ vulnerability to increasing extreme events, as well as the subsequent decline in ecosystem services^36^, need to be developed collaboratively between scientists and conservation practitioners, integrating research with applied conservation practice and feasible management interventions. At the same time this study underlines that a low-emission scenario reduces the number of species facing significant changes in extreme event exposure, emphasizing the need for ambitious climate change mitigation.

## Methods

### Extreme event data

The extreme event dataset was generated following the methodology by ref. ^37^ and is described in ref. ^38^ (analytical workflow in Supplementary Fig. 5). It is based on climate and climate impact projections from ISIMIP Phase 3b^11^.

Climate models (also called general circulation models) simulate the physical climate system. They use the laws of physics to estimate how temperature, precipitation, wind patterns, ocean circulation and other climate variables change over time under different greenhouse gas scenarios. The CMIP provides a standardized framework for running and comparing simulations from several climate models under consistent scenarios and protocols. Results from climate models form the backbone of the IPCC Working Group I report, that is, the physical science of climate change.

Climate impact models take the output of climate models as inputs and simulate how these climate variables affect natural or human systems through dynamic, process-based representations of physical, biological, geological and chemical processes in combination with socio-economic boundary conditions (for example, land use). For example, hydrological models simulate the terrestrial water cycle and translate changes in temperature and precipitation into changes in soil moisture or river discharge, by simulating processes such as water storage, evapotranspiration and runoff^39^. Vegetation models simulate vegetation composition and distribution by representing processes such as photosynthesis, plant growth, fire disturbance and vegetation structure. ISIMIP provides a harmonized framework for running and comparing climate impact models across different sectors under consistent simulation protocols, forcing data and scenarios, similar to how CMIP coordinates climate models. Climate impact model results form an important basis for IPCC Working Group II assessments on climate change impacts and adaptation.

Details on the hydrological and vegetation models used in this study and references for the model description papers are in Supplementary Table 1. Impact models are forced by daily bias-adjusted climate data from five CMIP6 climate models: GFDL-ESM4, UKESM1-0-LL, IPSL-CM6A-LR, MPI-ESM1-2-HR and MRI-ESM2-0. The climate models were selected for the ISIMIP3b simulation round based on how the models performed in the historical period, their structural independence and the presentation of processes, and representing the range of equilibrium climate sensitivity of the CMIP6 model ensemble^11^. Simulations were run for a historical (1850–2014) and a future (2015–2100) period. Future simulations are based on three emission scenarios: low (SSP1–2.6), medium–high (SSP3–7.0) and high (SSP5–8.5). In addition, all models ran baseline simulations for stable pre-industrial climate conditions for 1850–2100, which we refer to here as the ‘pre-industrial control simulation’. These long simulations allow for a more robust identification of extreme events return periods as they include natural climate variability^11,40^. Spatial resolution is 0.5° latitude × 0.5° longitude (for consistency among different impact categories).

Exposure to extreme events was calculated as the area exposed annually^37^. This aggregation to an annual time scale allowed for an intercomparison of different extreme event types, while acknowledging that extreme climate impacts typically occur on smaller time scales^38^.

Various approaches exist for defining extreme events (for example, droughts^41^ and heatwaves^42^). We defined extreme events relative to the historical distribution within each grid cell. This approach quantifies conditions that deviate substantially from local historical patterns. A grid cell is classified as experiencing extreme conditions when patterns exceed the 97.5th percentile (2.5th for droughts) of the pre-industrial control simulation (1850–2100) for that cell following the approach established by ref. ^37^. Percentile-based definitions are standard in climate extremes research, with thresholds typically ranging from 90th to 99th percentile^4^. Our choice is conservative within this range and maintains consistency with previous assessments^37^. Recent biodiversity studies have applied similar grid-based percentile thresholds to quantify species exposure to extreme events^8,33,43^.

Drought exposure was derived from soil moisture simulated by three global hydrological models (H08, JULES-W2 and WaterGAP2-2e; Supplementary Table 1), that is, going beyond meteorological droughts^41^. The hydrological models simulate soil moisture at root level, at ~1-m depth, at daily temporal resolution, which is then aggregated to average monthly soil moisture. Unlike extreme events such as heatwaves and floods, which evolve within days and can be captured at daily resolution, soil moisture droughts develop over longer periods (months and years) and are therefore analysed at monthly time scale^44^. Soil moisture responds slowly to meteorological changes due to physical buffering of water in the soil, making monthly aggregation appropriate for drought assessment^45^. A grid cell is classified as drought-affected in a given year if soil moisture falls below the 2.5th percentile of the pre-industrial control simulation for three or more consecutive months^37^. A soil moisture mask excludes dry grid cells where annual mean discharge is below 0.1 mm per day.

Heatwaves are calculated from daily near-surface air temperature from climate models using a two-step approach^38^. First, we identify heatwave periods using the HeatWave Magnitude Index daily (HWMId)^46^. The HWMId quantifies the intensity of all heatwave periods occurring in a year. A heatwave period is defined as at least three consecutive days for which daily maximum temperature is higher than the 90th percentile of daily maximum temperatures under pre-industrial climate conditions, centred on a 31-day window^37^. The index produces a single value that increases with the severity and persistence of the heatwave. Because thresholds are calculated separately for each grid cell using a 31-day moving window centred on each day of the year, the method accounts for both spatial and seasonal temperature variation. Short heatwaves (3–4 days) split across the December–January boundary may be missed, but this affects pre-industrial, historical and future periods equally and would lead to underestimation rather than overestimation of exposure. Second, to identify extreme heatwaves for this study, we classified a grid cell as exposed to an extreme heatwave if the HWMId exceeded the 97.5th percentile of the pre-industrial control simulation for that grid cell.

River flood is based on daily runoff simulated by three global hydrological models (H08, JULES-W2 and WaterGAP2-2e; Supplementary Table 1). The river routing model CaMa-Flood^47^ redistributes runoff along prescribed river networks to determine outflow (detailed in ref. ^48^). A generalized extreme value distribution was fitted to the annual maxima time series of daily outflow of the pre-industrial control simulation. For each year, a grid cell is classified as flooded if the return period of maximum annual outflow exceeds 40 years, which corresponds to the 97.5th percentile. This is a binary classification. Based on outflow CaMa-Flood determines flood depth and flooded area. To exclude very dry areas, grid cells for which less than 1% of area is flooded are excluded and the soil moisture mask from above was applied. Note that flooded area is upscaled from 0.25° × 0.25° resolution (the original resolution of CaMa-Flood output) to 0.5° × 0.5° to ensure consistency between impact types.

Wildfire exposure was based on burned area simulated by three global vegetation models (CLASSIC, LPJmL5-7-10-fire, VISIT; Supplementary Table 1). The vegetation models simulate burned area at sub-annual time step (daily by CLASSIC and LPJmL5-7-10-fire, and monthly by VISIT). Monthly burned area output is commonly used in fire modelling^49,50^, although some models have moved to finer temporal resolution. Burned area is summed to annual totals for all models. A wildfire is considered an extreme event if the yearly burned area exceeds the 97.5th percentile of the respective pre-industrial control simulation, consistent with the other event types and ref. ^37^.

For a detailed discussion of the limitations of this dataset we refer to ref. ^38^. One limitation is the approach chosen to define extreme events, as alternative thresholds could yield different results. We implemented a sensitivity analysis using 95th, 97.5th and 99th percentile thresholds (5th, 2.5th and 1st for droughts) and show that the choice of percentile threshold introduces substantially less variability than climate model and impact model selection (Supplementary Figs. 6–9), which is line with previous assessments^44^. Furthermore, although we identify extreme values from impact model outputs, these models can have challenges simulating extreme events^48^. Specifically, the fire models do not fully capture the characteristics of megafires^38^ that have been documented to affect species in recent studies.

### Change in extreme event exposure

For each 30-year period, we calculated the event frequency by dividing the number of years with events by 30. We then calculated the mean across all climate model–impact model combinations. Ensemble minimum refers to the minimum value across all model combinations and the ensemble maximum to the maximum value across all model combinations. Extreme event exposure was determined for two time periods. For the historical period, we calculated average exposure for a 30-year period centred on 2000 (1985–2014). For the future period, we calculated 30-year averages as a moving window centred on 2030–2085. The change in frequency was then determined as the difference between future and past periods. For example, a change in event frequency from 0 to 0.5 means that in a grid cell without any event in the historical period (frequency = 0), an event is projected to occur in 15 of the 30 years, that is, on average every second year (frequency = 0.5). Although we focus on event frequency to enable consistent comparison across event types, we acknowledge that intensity and duration are important for biological impacts^29^ and can vary independently of frequency.

To identify areas exposed to multiple extreme events, we first determined for each grid cell whether the frequency of each event type was ≥0.33 (corresponding to events occurring at least every third year on average). We then summed the number of different event types meeting this threshold per grid cell, for the years 2000, 2050 and 2085. We provide results for applying different thresholds ranging from 0.2 to 0.5 in Supplementary Figs. 10 and 11.

### Species and ecoregion exposure to extreme events

For the hotspot maps, we used species richness layers from the IUCN Red List of Threatened Species (v.2023-1, accessed August 2024) for amphibians, birds, mammals and reptiles. For each taxon, we used three types of layers: number of species per grid cell for Fig. 1a–d (species richness), number of threatened species for Supplementary Fig. 1 and rarity weighted richness for Supplementary Fig. 2 (aggregate importance of each grid cell to the species occurring there, unitless). IUCN-aggregated richness patterns are consistent with those from individual species range layers (Supplementary Fig. 12). The original data in Mollweide projection at 30-km resolution were reprojected to WGS84 and resampled to match the 0.5-degree ISIMIP grid.

For all quantitative analysis, we used range layers for individual species. Species range data were obtained from multiple sources: the IUCN Red List of Threatened Species^12^ (terrestrial mammals and amphibians), the Global Assessment of Reptile Distributions^13^ (GARD, reptiles) and BirdLife International^14^ (birds). Following IUCN guidelines for extent of occurrence maps, we included polygons for extant species of native, reintroduced and assisted colonization origin and all seasonality codes. The initial dataset comprised 7,731 amphibians; 10,992 birds; 5,593 mammals and 10,914 reptiles. We mapped species ranges to the ISIMIP grid, by calculating for each grid cell the proportion of overlap with the species range. Due to the spatial resolution, small islands are not included in the ISIMIP grid and extreme event data are not available. Species restricted to small islands not represented in the ISIMIP grid were excluded (126 amphibians, 430 birds, 117 mammals and 621 reptiles), resulting in a final dataset of 33,936 terrestrial vertebrate species (7,605 amphibians; 10,562 birds; 5,476 mammals and 10,293 reptiles).

Ecoregion polygons were obtained from the Ecoregions 2017^15^ dataset. The dataset includes 847 ecoregions, but 53 ecoregions had no overlap with ISIMIP land cells. We used the remaining 794 ecoregions for our analysis.

To quantify species exposure to extreme events, we calculated the proportion of grid cells exposed, and weighted each grid cell by how often it was exposed within a 30-year period, by grid cell area and the proportion of overlap between each cell and the species range. Weighting event exposure by how much each grid cell overlaps with the species range reduces overestimation for small-ranged species. Grid cell area in a 0.5-degree global grid vary due to the spherical geometry of Earth, necessitating area-based weighting. Similarly, for ecoregion exposure, we weighted grid cells by event frequency, grid cell area and the proportion of overlap between each cell and the ecoregion polygon. All analyses were conducted in R (v.4.1.0) using the packages ncdf4 (v.1.19), sf (v.1.0.8) and sp (v.1.5.0).

### Reporting summary

Further information on research design is available in the Nature Portfolio Reporting Summary linked to this article.

## Supplementary information

Supplementary Information Supplementary Tables 1–9 and Figs. 1–17.

Reporting Summary

## Data Availability

Climate projections, climate impact projections and extreme event data are available from the ISIMIP Repository (https://data.isimip.org/); specifically, heatwave and wildfire data can be downloaded from 10.48364/ISIMIP.920810 (ref. ^51^) and drought and river flood data from 10.48364/ISIMIP.792444 (ref. ^52^). Species richness layers were downloaded from the IUCN Red List of Threatened Species (https://www.iucnredlist.org/). Species range data were downloaded from the IUCN Red List of Threatened Species (for terrestrial mammals and amphibians), from the Global Assessment of Reptile Distributions (GARD, http://www.gardinitiative.org/) and BirdLife International (http://datazone.birdlife.org). Ecoregions were downloaded from https://ecoregions.appspot.com/.

## Code availability

The code to reproduce the results of this study is available via Zenodo at 10.5281/zenodo.18861352 (ref. ^53^).
