# Supplementary material for: Land vertebrates increasingly exposed to multiple extreme events by 2085
Source: Nat Ecol Evol. 2026 Apr 24;10(5):854–63. doi: 10.1038/s41559-026-03050-0 (PMC13167462; doi:10.1038/s41559-026-03050-0)
Supplement: Supplementary file 1 — Supplementary Tables 1–9 and Figs. 1–17. [file 41559_2026_3050_MOESM1_ESM.pdf]

nature ecology & evolution

Supplementary information https://doi.org/10.1038/s41559-026-03050-0

Land vertebrates increasingly exposed to multiple extreme events by 2085

In the format provided by the authors and unedited

Supplementary Information

Supplementary tables

Supplementary Table 1 | Climate impact models

Supplementary Table 2 | Exposure averaged across all four taxa

Supplementary Table 3 | Amphibian exposure

Supplementary Table 4 | Bird exposure

Supplementary Table 5 | Mammal exposure

Supplementary Table 6 | Reptile exposure

Supplementary Table 7 | Ecoregion exposure

Supplementary Table 8 | Exposure to different event combinations

Supplementary Table 9 | Table 1 with references

Supplementary figures

Supplementary Fig. 1 | Exposure of threatened species richness to extreme events

Supplementary Fig. 2 | Exposure of rarity-weighted richness to extreme events

Supplementary Fig. 3 | Exposure of amphibians to droughts

Supplementary Fig. 4 | Exposure to different event combinations

Supplementary Fig. 5 | Conceptual framework of the analytical workflow

Supplementary Fig. 6 | Sensitivity analysis for threshold used to define heatwaves

Supplementary Fig. 7 | Sensitivity analysis for threshold used to define wildfires

Supplementary Fig. 8 | Sensitivity analysis for threshold used to define droughts

Supplementary Fig. 9 | Sensitivity analysis for threshold used to define floods

Supplementary Fig. 10 | Sensitivity analysis for multiple events for species

Supplementary Fig. 11 | Sensitivity analysis for multiple events for ecoregions

Supplementary Fig. 12 | Species richness patterns derived from different data sources.

Supplementary Fig. 13 | Exposure of amphibians to (a) heatwaves, (b) wildfires, (c) droughts, and (d) river floods for each climate model – impact model combination

Supplementary Fig. 14 | Exposure of birds for each climate model – impact model combination

Supplementary Fig. 15 | Exposure of mammals for each climate model – impact model combination

Supplementary Fig. 16 | Exposure of reptiles for each climate model – impact model combination

Supplementary Fig. 17 | Exposure of ecoregions for each climate model – impact model combination

Supplementary Table 1. Climate impact models. Information on climate impact models that contributed simulations for the extreme event dataset.

Extreme event type	Model	Model version	Modelling group (names of modellers and their institutions)	Main reference
Drought / River flood	H08		Kedar Otta: otta.kedar@nies.go.jp, 0000-0002-2540-9879, NIES (Japan)	Hanasaki et al., 2018
	JULES-W2	JULESvn6.2	Manolis Grillakis: egrillakis@tuc.gr, 0000-0002-4228-1803, Technical University of Crete (Greece)<br>Aristeidis Koutroulis: akoutroulis@tuc.gr, 0000-0002-2999-7575, Technical University of Crete (Greece)	Best et al., 2011
	WaterGAP2-2e	WaterGAP2.2e	Hannes Müller Schmied: hannes.mueller.schmied@em.uni-frankfurt.de, 0000-0001-5330-9923, Institute of Physical Geography (IPG), Goethe-University Frankfurt (Germany)	Müller Schmied *et al* 2021, 2024
Wildfire	CLASSIC	CLASSICv1.4	Sian Kou-Giesbrecht: sian.kougiesbrecht@gmail.com, 0000-0002-4086-0561, Canadian Centre for Climate Modelling and Analysis (Canada)	Melton et al., 2020
	LPJmL5-7-10-fire	5.7.10-fire	Sebastian Ostberg: ostberg@pik-potsdam.de, 0000-0002-2368-7015, Potsdam Institute for Climate Impact Research (Germany)	Wirth *et al* 2024, Oberhagemann *et al* 2024
	VISIT		Akihiko Ito: akihikoito@g.ecc.u-tokyo.ac.jp, 0000-0001-5265-0791, The University of Tokyo (Japan)	Ito 2019

Supplementary Table 2. Exposure averaged across all four taxa. Numbers from Supplementary Table 3 – 6 averaged across the four taxa. Results for exposure to different extreme event types calculated as mean across all combinations of climate models and impact models. Listed are absolute values for the respective year (% range exposed), and in brackets the minimum and maximum of the model ensemble. Mean change % exposed is the difference to the year 2000, where in brackets the minimum is the lowest value for change relative to year 2000 by any ensemble member (and maximum the maximum value).

		**SSP1-2.6**		**SSP3-7.0**		**SSP5-8.5**	
	**2000**	**2050**	**2085**	**2050**	**2085**	**2050**	**2085**
**Heatwave**							
% range exposed	18.4 [13.8 – 27.2]	63.1 [44.7 – 81.2]	65.2 [43.1 – 86.8]	73.9 [59.2 – 90.3]	92.5 [84.9 – 98.8]	77.4 [62.8 – 91.2]	95.6 [89.3 – 99.6]
mean change % range exposed		44.7 [30.7 – 64.1]	46.9 [29.1 – 69.7]	55.6 [45.2 – 73.1]	74.1 [69.0 – 81.7]	59.0 [48.8 – 74.0]	77.2 [71.4 – 82.4]
**Wildfire**							
% range exposed	7.9 [5.7 – 11.3]	13.4 [9.2 – 19.2]	13.8 [9.4 – 20.9]	16.1 [11.3 – 22.4]	24.8 [16.5 – 35.3]	17.9 [12.1 – 24.8]	28.1 [19.2 – 40.9]
mean change % range exposed		5.6 [2.1 – 11.0]	5.9 [2.3 – 12.8]	8.3 [4.1 – 14.3]	16.9 [9.5 – 27.1]	10.1 [5.0 – 16.6]	20.2 [12.1 – 32.8]
**Drought**							
% range exposed	4.6 [2.9 – 7.3]	6.0 [3.0 – 10.3]	6.0 [3.4 – 11.5]	7.9 [3.3 – 15.3]	14.3 [5.2 – 29.1]	9.1 [4.3 – 17.1]	16.7 [5.6 – 33.8]
mean change % range exposed		1.4 [-0.7 – 6.2]	1.5 [-1.2 – 7.4]	3.4 [-0.4 – 9.5]	9.8 [1.7 – 25.0]	4.5 [0.2 – 12.4]	12.1 [1.2 – 29.7]
**River Flood**							
% range exposed	1.7 [1.1 – 2.6]	2.9 [1.7 – 4.1]	3.0 [1.7 – 4.4]	3.4 [1.6 – 5.1]	5.4 [1.9 – 9.5]	3.6 [1.8 – 5.3]	6.8 [2.3 – 11.4]
mean change % range exposed		1.2 [0.6 – 2.0]	1.4 [0.6 – 2.7]	1.7 [0.5 – 3.4]	3.7 [0.8 – 7.4]	1.9 [0.6 – 3.5]	5.1 [1.1 – 9.5]
**Multiple events**							
% species exposed 2 EE types	1.0 [0.1 – 3.8]	7.9 [4.6 – 10.0]	8.6 [5.2 – 12.0]	14.0 [10.1 – 17.6]	36.2 [26.3 – 44.5]	18.2 [12.8 – 22.6]	44.4 [36.4 – 54.2]
% species exposed 3 EE types	0.3 [0.0 – 1.5]	0.7 [0.2 – 1.5]	0.6 [0.3 – 1.2]	2.1 [0.5 – 4.9]	9.8 [4.1 – 14.4]	3.1 [0.8 – 5.8]	14.1 [8.0 – 19.1]

Supplementary Table 3. Amphibian exposure for n=7,605 amphibian species. Results for exposure to different extreme event types calculated as mean across all combinations of climate models and impact models. Listed are absolute values for the respective year (% range exposed), and in brackets the minimum and maximum of the model ensemble. Mean change % exposed is the difference to the year 2000, where in brackets the minimum is the lowest value for change relative to year 2000 by any ensemble member (and maximum the maximum value). Results also show the number of species with ≥25% area exposed to extreme events (as well as 50% and 75%), calculated by first averaging exposure across all combinations of climate models and impact models for each species, and then counting species above the threshold. Minimum and maximum values represent the count of species exceeding the 25% threshold (and 50% and 75% threshold respectively) in individual model projections. The minimum count can exceed the mean because individual models may show strong exposure in different areas, resulting in fewer species meeting the threshold when exposures are averaged across all models.

		**SSP1-2.6**		**SSP3-7.0**		**SSP5-8.5**	
	**2000**	**2050**	**2085**	**2050**	**2085**	**2050**	**2085**
**Heatwave**							
% range exposed	20.1 [14.3 – 28.1]	65.6 [46.4 – 84.2]	67.3 [43.9 – 89.5]	76.0 [61.4 – 92.1]	93.7 [86.9 – 99.1]	79.6 [65.2 – 93.1]	96.6 [91.5 – 99.7]
no. species ≥25% exposed	2,210 [1,240 – 3,989]	7,300 [5,780 – 7,471]	7,349 [5,388 – 7,516]	7,514 [6,672 – 7,562]	7,605 [7,448 – 7,605]	7,526 [6,865 – 7,554]	7,605 [7,489 – 7,605]
no. species ≥50% exposed	53 [210 – 809]	6,010 [3,311 – 6,959]	6,209 [3,059 – 7,244]	6,849 [5,089 – 7,384]	7,515 [7,036 – 7,601]	7,079 [5,431 – 7,407]	7,596 [7221 – 7,605]
no. species ≥75% exposed	0 [28 – 240]	2,708 [1,143 – 5,868]	2,919 [1,155 – 6,594]	4,447 [2,784 – 6,888]	7,137 [6,201 – 7,533]	5,127 [3,429 – 7,066]	7,414 [6,707 – 7,590]
mean change % range exposed		45.5 [32.1 – 63.6]	47.3 [29.7 – 68.9]	55.9 [47.1 – 71.5]	73.6 [68.8 – 78.5]	59.5 [51.0 – 72.5]	76.5 [70.8 – 79.1]
**Wildfire**							
% range exposed	8.1 [5.7 – 12.6]	13.3 [9.4 – 18.3]	13.4 [9.3 – 19.6]	15.9 [11.3 – 20.6]	25.4 [17.4 – 34.7]	18.3 [12.7 – 23.8]	29.3 [21 – 40.7]
no. species ≥25% exposed	11 [96 – 826]	557 [407 – 1,627]	498 [406 – 2,049]	1,202 [785 – 2,291]	3,407 [1,803 – 4,770]	1,938 [1,102 – 2,993]	4,085 [2,505 – 5,270]
no. species ≥50% exposed	0 [0 – 224]	3 [21 – 370]	5 [39 – 308]	7 [54 – 579]	398 [432 – 1,678]	38 [110 – 669]	1,078 [786 – 2,686]
no. species ≥75% exposed	0 [0 – 75]	1 [4 – 135]	1 [8 – 99]	2 [10 – 134]	13 [70 – 463]	2 [11 – 195]	45 [198 – 788]
mean change % range exposed		5.3 [2.0 – 10.0]	5.4 [1.9 – 11.4]	7.9 [4.2 – 12.5]	17.3 [10.1 – 26.4]	10.2 [5.4 – 15.5]	21.3 [13.6 – 32.5]
**Drought**							
% range exposed	4.8 [2.6 – 8.5]	6.5 [2.5 – 11.0]	6.4 [2.8 – 12.1]	8.7 [2.7 – 17.7]	16.4 [4.5 – 32.4]	10.1 [3.6 – 20.1]	19.4 [5.1 – 37.1]
no. species ≥25% exposed	0 [6 – 490]	16 [43 – 800]	36 [63 – 857]	182 [55 – 1989]	1508 [300 – 3829]	342 [115 – 2433]	2263 [411 – 4558]
no. species ≥50% exposed	0 [0 – 107]	0 [0 – 173]	0 [1 – 108]	1 [4 – 398]	184 [37 – 1714]	3 [4 – 510]	377 [43 – 2305]
no. species ≥75% exposed	0 [0 – 19]	0 [0 – 24]	0 [0 – 24]	0 [0 – 53]	2 [3 – 753]	0 [0 – 95]	13 [6 – 1022]
mean change % range exposed		1.7 [-1.0 – 6.9]	1.6 [-1.9 – 8.1]	3.9 [-0.8 – 10.9]	11.6 [1.5 – 28.3]	5.3 [-0.2 – 14.0]	14.6 [0.8 – 33.1]
**River Flood**							
% range exposed	1.6 [1.0 – 2.4]	2.7 [1.7 – 3.8]	2.8 [1.7 – 4.0]	3.1 [1.5 – 4.8]	4.9 [1.7 – 8.9]	3.2 [1.7 – 5.0]	6.3 [2.1 – 10.7]
no. species ≥25% exposed	0 [0 – 6]	0 [0 – 65]	0 [0 – 54]	3 [0 – 121]	40 [0 – 623]	3 [0 – 186]	128 [6 – 970]
no. species ≥50% exposed	0 [0 – 0]	0 [0 – 6]	0 [0 – 8]	0 [0 – 10]	0 [0 – 80]	0 [0 – 28]	2 [0 – 196]
no. species ≥75% exposed	0 [0 – 0]	0 [0 – 1]	0 [0 – 2]	0 [0 – 2]	0 [0 – 6]	0 [0 – 6]	0 [0 – 35]
mean change % range exposed		1.1 [0.6 – 1.8]	1.2 [0.6 – 2.5]	1.5 [0.4 – 2.9]	3.4 [0.6 – 6.9]	1.6 [0.6 – 2.9]	4.7 [1.0 – 8.8]
**Multiple events**							
% species exposed 2 EE types	1.3 [0.1 – 5.7]	7.2 [3.1 – 10.3]	7.5 [3.6 – 10.5]	13.3 [7.6 – 18.8]	36.0 [22.9 – 43.8]	18.5 [11.2 – 23.4]	45.3 [38.2 – 53.4]

no. species ≥25% exposed to 2 EE types	6 [3 – 569]	537 [282 – 1,028]	545 [312 – 1,056]	1,375 [728 – 1,891]	4,319 [2,386 – 4,328]	2,244 [1,176 – 2,320]	5,186 [3,677 – 5,105]
no. species ≥50% exposed to 2 EE types	0 [0 – 329]	87 [112 – 620]	99 [162 – 504]	352 [356 – 1,306]	2,241 [1,461 – 3,153]	659 [581 – 1,683]	3,272 [2,758 – 4,065]
no. species ≥75% exposed to 2 EE types	0 [0 – 213]	14 [57 – 368]	28 [92 – 308]	80 [207 – 838]	887 [944 – 2,398]	222 [326 – 1,102]	1,605 [1,917 – 3,106]
% species exposed 3 EE types	0.5 [0.0 – 2.4]	0.9 [0.2 – 2.4]	0.7 [0.3 – 1.8]	2.5 [0.4 – 6.2]	11.6 [4.3 – 17.2]	3.8 [0.6 – 7.5]	17.1 [9.1 – 23.3]
no. species ≥25% exposed to 3 EE types	0 [0 – 232]	8 [20 – 239]	18 [17 – 165]	96 [30 – 641]	1,269 [394 – 1,581]	229 [47 – 788]	2,036 [888 – 2,165]
no. species ≥50% exposed to 3 EE types	0 [0 – 125]	0 [8 – 131]	2 [7 – 82]	11 [13 – 391]	390 [221 – 1,255]	36 [21 – 497]	801 [512 – 1,681]
no. species ≥75% exposed to 3 EE types	0 [0 – 84]	0 [6 – 82]	0 [3 – 52]	0 [7 – 209]	134 [156 – 973]	2 [17 – 274]	318 [360 – 1,312]

Supplementary Table 4. Bird exposure for n=10,562 bird species. Details described in heading of Supplementary Table 3.

		**SSP1-2.6**		**SSP3-7.0**		**SSP5-8.5**	
	**2000**	**2050**	**2085**	**2050**	**2085**	**2050**	**2085**
**Heatwave**							
% range exposed	18.8 [14.1 – 28.0]	63.8 [44.6 – 82.4]	65.7 [43.2 – 87.4]	74.2 [58.9 – 90.5]	92 [84.2 – 98.7]	77.6 [63.0 – 91.4]	95.0 [88.4 – 99.5]
no. species ≥25% exposed	2,485 [1,252 – 6,067]	10,189 [8,683 – 10,430]	10,249 [8,432 – 10,479]	10,487 [9,667 – 10,544]	10,551 [10,492 – 10,556]	10,508 [9,805 – 10,540]	10,555 [10,477 – 10,556]
no. species ≥50% exposed	24 [62 – 741]	7,970 [4,078 – 9,871]	8,555 [3,818 – 10,026]	9,434 [6,864 – 10,296]	10,503 [9,829 – 10,547]	9,798 [7,338 – 10,324]	10,537 [10,054 – 10,550]
no. species ≥75% exposed	0 [2 – 31]	3,477 [897 – 7,542]	3,774 [908 – 8,870]	5,905 [2,851 – 9,450]	9,825 [8,104 – 10,504]	6,674 [4,007 – 9,711]	10,255 [8,959 – 10,537]
mean change % range exposed		45.0 [30.5 – 65.2]	46.9 [29.1 – 70.2]	55.4 [44.8 – 73.2]	73.1 [68 – 81.4]	58.8 [48.9 – 74.1]	76.2 [70.4 – 82.2]
**Wildfire**							
% range exposed	7.8 [5.3 – 10.8]	13.4 [9.2 – 19.2]	13.7 [9.5 – 20.9]	16.0 [11.3 – 22.2]	24.2 [16.2 – 34.3]	17.7 [12.2 – 24.3]	27.4 [19.1 – 39.9]
no. species ≥25% exposed	0 [3 – 500]	301 [105 – 2,278]	213 [161 – 3,000]	913 [371 – 3,755]	4,597 [1,941 – 7,597]	2,232 [563 – 4,903]	5,435 [3,316 – 7,969]
no. species ≥50% exposed	0 [0 – 20]	0 [1 – 115]	0 [4 – 174]	0 [7 – 309]	85 [132 – 1,541]	0 [16 – 378]	824 [318 – 3,368]
no. species ≥75% exposed	0 [0 – 3]	0 [0 – 13]	0 [0 – 13]	0 [0 – 32]	0 [7 – 160]	0 [0 – 28]	0 [30 – 483]
mean change % range exposed		5.6 [2.1 – 11.4]	5.9 [2.3 – 13.1]	8.2 [3.9 – 14.4]	16.4 [9.1 – 26.5]	9.9 [5.0 – 16.5]	19.7 [12.0 – 32.1]
**Drought**							
% range exposed	4.6 [3 – 7.2]	6.1 [3.0 – 10.8]	6.1 [3.4 – 12]	8.0 [3.3 – 15.9]	14.2 [5.1 – 29.8]	9.1 [4.3 – 17.3]	16.5 [5.7 – 34.4]
no. species ≥25% exposed	0 [0 – 230]	1 [3 – 739]	2 [13 – 740]	39 [41 – 2338]	1648 [277 – 5040]	111 [46 – 2594]	2175 [362 – 6258]
no. species ≥50% exposed	0 [0 – 8]	0 [0 – 17]	0 [0 – 23]	0 [0 – 110]	33 [22 – 1785]	0 [2 – 379]	142 [33 – 2304]
no. species ≥75% exposed	0 [0 – 0]	0 [0 – 1]	0 [0 – 4]	0 [0 – 6]	0 [0 – 797]	0 [0 – 7]	0 [2 – 986]
mean change % range exposed		1.5 [-0.4 – 6.6]	1.5 [-1.2 – 7.8]	3.4 [-0.3 – 9.8]	9.6 [1.7 – 25.6]	4.5 [0.4 – 12.9]	12.0 [1.0 – 30.3]
**River Flood**							
% range exposed	1.7 [1.1 – 2.6]	3.1 [1.7 – 4.2]	3.2 [1.8 – 4.7]	3.5 [1.6 – 5.3]	5.7 [2.0 – 10.0]	3.8 [1.8 – 5.6]	7.3 [2.4 – 12.1]

no. species ≥25% exposed	0 [0 – 3]	0 [0 – 86]	0 [0 – 84]	0 [0 – 204]	20 [3 – 855]	1 [0 – 371]	185 [3 – 1362]
no. species ≥50% exposed	0 [0 – 0]	0 [0 – 1]	0 [0 – 3]	0 [0 – 3]	0 [0 – 35]	0 [0 – 17]	0 [0 – 395]
no. species ≥75% exposed	0 [0 – 0]	0 [0 – 0]	0 [0 – 0]	0 [0 – 0]	0 [0 – 1]	0 [0 – 1]	0 [0 – 12]
mean change % range exposed		1.3 [0.6 – 2.2]	1.5 [0.7 – 2.9]	1.8 [0.5 – 3.6]	4.0 [0.9 – 7.8]	2.1 [0.7 – 3.8]	5.5 [1.3 – 9.9]
**Multiple events**							
% species exposed 2 EE types	0.9 [0.1 – 3.3]	8.0 [4.2 – 9.9]	8.6 [5.0 – 12.8]	13.9 [9.4 – 17.7]	35.9 [25.7 – 45.7]	18.0 [12.5 – 22.8]	43.9 [36.3 – 54.3]
no. species ≥25% exposed to 2 EE types	0 [0 – 307]	414 [227 – 1,619]	397 [332 – 1,484]	1,741 [898 – 2,779]	6,886 [4,962 – 7,818]	3,235 [1,665 – 4,039]	8,181 [5,813 – 8,931]
no. species ≥50% exposed to 2 EE types	0 [0 – 40]	14 [36 – 458]	28 [76 – 209]	85 [142 – 1,347]	2,761 [1358 – 4507]	242 [249 – 1,718]	4,209 [2,751 – 6,185]
no. species ≥75% exposed to 2 EE types	0 [0 – 9]	0 [10 – 130]	1 [20 – 43]	6 [43 – 307]	343 [320 – 1961]	25 [58 – 493]	1,160 [942 – 2,611]
% species exposed 3 EE types	0.3 [0.0 – 1.3]	0.7 [0.1 – 1.4]	0.6 [0.3 – 1.0]	2.2 [0.4 – 5.6]	10.0 [3.7 – 14.8]	3.4 [0.7 – 6.7]	14.0 [7.9 – 18.8]
no. species ≥25% exposed to 3 EE types	0 [0 – 74]	0 [0 – 99]	0 [0 – 23]	19 [3 – 817]	1,477 [256 – 2,067]	86 [11 – 1,078]	2,162 [993 – 2,537]
no. species ≥50% exposed to 3 EE types	0 [0 – 6]	0 [0 – 14]	0 [0 – 3]	0 [2 – 201]	161 [59 – 1,308]	1 [0 – 364]	744 [235 – 1,622]
no. species ≥75% exposed to 3 EE types	0 [0 – 4]	0 [0 – 2]	0 [0 – 1]	0 [0 – 37]	18 [7 – 720]	0 [0 – 80]	96 [77 – 952]

Supplementary Table 5. Mammal exposure for n=5,476 mammal species. Details described in heading of Supplementary Table 3.

		SSP1-2.6		SSP3-7.0		SSP5-8.5	
	**2000**	**2050**	**2085**	**2050**	**2085**	**2050**	**2085**
**Heatwave**							
% range exposed	16.9 [13.0 – 25.7]	60.8 [41.9 – 79.9]	63.0 [40.4 – 86.1]	71.8 [56.4 – 89.5]	91.5 [82.8 – 98.9]	75.5 [59.6 – 90.6]	94.8 [87.2 – 99.6]
no. species ≥25% exposed	1,026 [619 – 2,677]	5,210 [4,106 – 5,365]	5,259 [3,879 – 5,421]	5,427 [4,834 – 5,467]	5,475 [5,345 – 5,476]	5,441 [4,881 – 5,463]	5,476 [5,322 – 5,476]
no. species ≥50% exposed	5 [34 – 393]	3,868 [1,887 – 4,952]	4,191 [1,811 – 5,150]	4,729 [3,302 – 5,299]	5,444 [4,981 – 5,475]	4,965 [3,512 – 5,330]	5,469 [5,086 – 5,475]
no. species ≥75% exposed	0 [0 – 23]	1,467 [502 – 3,663]	1,627 [500 – 4,384]	2,753 [1,358 – 4,761]	5,012 [4,136 – 5,445]	3,139 [1,869 – 4,964]	5,296 [4,547 – 5,471]
mean change % range exposed		43.9 [28.8 – 64.8]	46.0 [27.4 – 70.9]	54.9 [43.3 – 74.4]	74.5 [69.7 – 83.7]	58.6 [46.6 – 75.5]	77.8 [72.9 – 84.5]
**Wildfire**							
% range exposed	7.8 [5.6 – 10.9]	13.6 [9.5 – 19.3]	14.0 [9.8 – 20.8]	16.3 [11.7 – 22.6]	24.6 [17.1 – 34.6]	17.9 [12.5 – 24.8]	27.7 [19.3 – 40.1]
no. species ≥25% exposed	2 [14 – 306]	289 [194 – 1,288]	283 [236 – 1,678]	678 [420 – 1,986]	2,449 [1,243 – 3,698]	1,194 [546 – 2,509]	2,904 [1,600 – 3,928]
no. species ≥50% exposed	0 [0 – 46]	1 [12 – 161]	2 [16 – 175]	3 [21 – 301]	111 [184 – 1,031]	6 [39 – 384]	449 [350 – 1,870]
no. species ≥75% exposed	0 [0 – 15]	1 [2 – 28]	1 [3 – 27]	1 [2 – 48]	2 [17 – 162]	1 [2 – 53]	3 [41 – 293]
mean change % range exposed		5.8 [2.5 – 11.1]	6.3 [2.8 – 12.6]	8.5 [4.5 – 14.5]	16.8 [10.1 – 26.5]	10.2 [5.5 – 16.7]	19.9 [12.3 – 31.9]
**Drought**							
% range exposed	4.5 [3.1 – 6.8]	5.9 [3.4 – 10.2]	6 [3.8 – 11.1]	7.8 [4.0 – 14.5]	13.6 [5.8 – 27.8]	8.8 [4.9 – 16.0]	15.7 [6.2 – 32.6]

no. species ≥25% exposed	0 [1 – 152]	2 [8 – 376]	3 [19 – 398]	30 [57 – 1027]	733 [252 – 2,525]	100 [56 – 1,156]	1,016 [286 – 3,097]
no. species ≥50% exposed	0 [0 – 19]	0 [0 – 32]	0 [0 – 45]	0 [0 – 73]	21 [36 – 851]	0 [1 – 183]	81 [50 – 1,221]
no. species ≥75% exposed	0 [0 – 1]	0 [0 – 6]	0 [0 – 8]	0 [0 – 8]	0 [2 – 316]	0 [0 – 16]	0 [7 – 429]
mean change % range exposed		1.4 [-0.4 – 6.0]	1.4 [-0.8 – 7.0]	3.2 [0.0 – 9.0]	9.1 [2.0 – 23.6]	4.3 [0.7 – 11.8]	11.1 [1.4 – 28.4]
**River Flood**							
% range exposed	1.8 [1.1 – 2.7]	3.1 [1.7 – 4.4]	3.2 [1.7 – 4.7]	3.6 [1.7 – 5.5]	5.8 [2.0 – 10.3]	3.9 [1.7 – 5.8]	7.4 [2.3 – 12.3]
no. species ≥25% exposed	0 [0 – 14]	0 [0 – 89]	0 [0 – 83]	5 [0 – 176]	36 [4 – 532]	8 [0 – 287]	164 [4 – 767]
no. species ≥50% exposed	0 [0 – 1]	0 [0 – 13]	0 [0 – 16]	0 [0 – 24]	0 [0 – 60]	0 [0 – 41]	2 [0 – 318]
no. species ≥75% exposed	0 [0 – 0]	0 [0 – 2]	0 [0 – 2]	0 [0 – 4]	0 [0 – 9]	0 [0 – 8]	0 [0 – 57]
mean change % range exposed		1.3 [0.5 – 2.2]	1.4 [0.6 – 3.0]	1.9 [0.6 – 3.7]	4.1 [0.9 – 8.0]	2.2 [0.6 – 4.0]	5.7 [1.2 – 10.3]
**Multiple events**							
% species exposed 2 EE types	0.9 [0.1 – 3.1]	8.3 [5.1 – 9.7]	9.0 [5.6 – 12.7]	14.5 [11.6 – 17.1]	36.5 [28.1 – 45.9]	18.4 [13.7 – 22.7]	44.5 [35.8 – 55.1]
no. species ≥25% exposed to 2 EE types	0 [2 – 183]	350 [245 – 695]	397 [272 – 872]	989 [758 – 1,455]	3,528 [2,364 – 3,852]	1,659 [1,048 – 1,951]	4,107 [2,706 – 4,377]
no. species ≥50% exposed to 2 EE types	0 [0 – 56]	29 [61 – 240]	36 [87 – 249]	139 [242 – 578]	1,542 [1,102 – 2,357]	279 [290 – 823]	2,208 [1,748 – 3,179]
no. species ≥75% exposed to 2 EE types	0 [0 – 20]	5 [31 – 117]	6 [43 – 85]	18 [105 – 240]	328 [431 – 1,199]	36 [102 – 303]	775 [820 – 1,787]
% species exposed 3 EE types	0.2 [0.0 – 1.0]	0.6 [0.1 – 1.2]	0.5 [0.3 – 1.0]	2.0 [0.6 – 4.5]	9.1 [4.1 – 13.3]	2.9 [0.8 – 5.3]	12.8 [7.7 – 17.8]
no. species ≥25% exposed to 3 EE types	0 [0 – 49]	0 [1 – 68]	0 [3 – 38]	14 [24 – 337]	673 [207 – 957]	71 [21 – 428]	996 [523 – 1,307]
no. species ≥50% exposed to 3 EE types	0 [0 – 14]	0 [1 – 19]	0 [0 – 7]	0 [7 – 135]	90 [73 – 584]	0 [6 – 169]	314 [168 – 726]
no. species ≥75% exposed to 3 EE types	0 [0 – 5]	0 [0 – 5]	0 [0 – 3]	0 [1 – 40]	13 [24 – 315]	0 [0 – 83]	43 [61 – 436]

Supplementary Table 6. Reptile exposure for n=10,293 reptile species. Details described in heading of Supplementary Table 3.

		**SSP1-2.6**		**SSP3-7.0**		**SSP5-8.5**	
	**2000**	**2050**	**2085**	**2050**	**2085**	**2050**	**2085**
**Heatwave**							
% range exposed	17.7 [13.9 – 26.9]	62.2 [46.0 – 78.4]	64.9 [45.0 – 84.3]	73.7 [60.3 – 88.9]	93.0 [85.7 – 98.7]	77.0 [63.2 – 89.7]	96.0 [90.0 – 99.6]
no. species ≥25% exposed	2,106 [1,602 – 5,103]	9,827 [7,955 – 10,035]	9,862 [7,603 – 10,139]	10,182 [9,094 – 10,248]	10,295 [10,118 – 10,293]	10,198 [9,244 – 10,248]	10,299 [10,162 – 10,293]
no. species ≥50% exposed	24 [139 – 977]	7,500 [4,260 – 8,964]	8,071 [4,169 – 9,445]	9,155 [6,676 – 9,886]	10,194 [9,472 – 10,285]	9,479 [7,131 – 9,957]	10,280 [9,800 – 10,290]
no. species ≥75% exposed	0 [10 – 116]	3,021 [1,583 – 6,662]	3,437 [1,614 – 7,813]	5,495 [3,504 – 8,738]	9,617 [8,180 – 10,206]	6,282 [4,083 – 9,021]	10,027 [8,883 – 10,283]
mean change % range exposed		44.6 [31.4 – 62.8]	47.2 [30.4 – 68.7]	56.1 [45.7 – 73.3]	75.3 [69.3 – 83.1]	59.3 [48.7 – 74.1]	78.3 [71.6 – 84.0]
**Wildfire**							
% range exposed	7.9 [6.2 – 11.1]	13.4 [8.7 – 19.9]	14.0 [8.9 – 22.5]	16.3 [10.7 – 24.1]	24.8 [15.4 – 37.4]	17.8 [10.9 – 26.2]	28.0 [17.2 – 42.9]

no. species ≥25% exposed	13 [94 – 733]	741 [406 – 2,710]	807 [430 – 3,639]	1,624 [806 – 4,244]	4,456 [1,949 – 7,062]	2,223 [985 – 4,856]	5,384 [2,552 – 7,564]
no. species ≥50% exposed	0 [8 – 143]	8 [37 – 474]	10 [56 – 548]	24 [71 – 787]	476 [385 – 2,786]	59 [104 – 1,087]	1,075 [622 – 4,082]
no. species ≥75% exposed	0 [1 – 38]	1 [10 – 109]	1 [13 – 110]	2 [17 – 163]	20 [83 – 616]	3 [15 – 200]	43 [131 – 1,051]
mean change % range exposed		5.5 [1.9 – 11.4]	6.2 [2.2 – 14.0]	8.5 [3.9 – 15.6]	16.9 [8.7 – 29.0]	9.9 [4.1 – 17.7]	20.1 [10.5 – 34.4]
**Drought**							
% range exposed	4.4 [2.9 – 6.6]	5.4 [3.1 – 9.3]	5.7 [3.6 – 10.9]	7.3 [3.4 – 13.3]	13.1 [5.4 – 26.4]	8.2 [4.2 – 15.0]	15.2 [5.4 – 30.9]
no. species ≥25% exposed	1 [6 – 385]	8 [47 – 733]	20 [73 – 961]	118 [149 – 1,751]	1,444 [499 – 4,333]	263 [190 – 2,038]	1,935 [543 – 5,339]
no. species ≥50% exposed	0 [0 – 73]	0 [6 – 108]	0 [9 – 81]	0 [15 – 192]	133 [114 – 1,703]	0 [21 – 381]	280 [120 – 2,290]
no. species ≥75% exposed	0 [0 – 10]	0 [0 – 10]	0 [0 – 18]	0 [4 – 30]	0 [21 – 597]	0 [4 – 67]	5 [23 – 808]
mean change % range exposed		1.0 [-1.1 – 5.3]	1.4 [-1.0 – 6.9]	2.9 [-0.5 – 8.2]	8.8 [1.4 – 22.4]	3.8 [0.0 – 11.0]	10.8 [1.4 – 27.0]
**River Flood**							
% range exposed	1.7 [1.1 – 2.5]	2.9 [1.7 – 3.9]	2.9 [1.7 – 4.3]	3.3 [1.7 – 4.9]	5.1 [1.8 – 8.7]	3.4 [1.8 – 5.0]	6.3 [2.3 – 10.4]
no. species ≥25% exposed	0 [0 – 11]	0 [0 – 61]	0 [0 – 71]	2 [0 – 158]	45 [8 – 803]	6 [0 – 277]	205 [13 – 1,175]
no. species ≥50% exposed	0 [0 – 0]	0 [0 – 8]	0 [0 – 8]	0 [0 – 16]	0 [0 – 104]	0 [0 – 33]	0 [0 – 306]
no. species ≥75% exposed	0 [0 – 0]	0 [0 – 0]	0 [0 – 0]	0 [0 – 2]	0 [0 – 11]	0 [0 – 3]	0 [0 – 47]
mean change % range exposed		1.2 [0.5 – 2.0]	1.3 [0.6 – 2.6]	1.6 [0.5 – 3.2]	3.4 [0.6 – 7.0]	1.7 [0.6 – 3.2]	4.7 [1.1 – 8.8]
**Multiple events**							
% species exposed 2 EE types	0.9 [0.1 – 3.2]	8.1 [5.8 – 10.0]	9.4 [6.5 – 12.0]	14.4 [11.6 – 16.6]	36.3 [28.6 – 42.7]	18.1 [13.7 – 21.4]	44.0 [35.3 – 54.1]
no. species ≥25% exposed to 2 EE types	8 [7 – 392]	849 [685 – 1,406]	1,047 [746 – 1,619]	2,012 [1,458 – 2,366]	5,975 [4,052 – 6,119]	2,887 [1,879 – 3,039]	7,162 [4,652 – 7,416]
no. species ≥50% exposed to 2 EE types	0 [3 – 194]	161 [294 – 562]	223 [362 – 769]	532 [725 – 1,107]	2,979 [,2381 – 4,208]	876 [885 – 1,698]	3,966 [3,353 – 5,711]
no. species ≥75% exposed to 2 EE types	0 [2 – 107]	41 [179 – 350]	59 [211 – 377]	148 [437 – 669]	1,161 [1,392 – 2,629]	214 [498 – 979]	1,892 [2,051 – 3,826]
% species exposed 3 EE types	0.2 [0.0 – 1.2]	0.5 [0.3 – 1.1]	0.6 [0.2 – 1.2]	1.7 [0.7 – 3.2]	8.7 [4.3 – 12.3]	2.5 [1.0 – 3.8]	12.4 [7.1 – 16.7]
no. species ≥25% exposed to 3 EE types	0 [0 – 131]	10 [16 – 137]	21 [18 – 114]	79 [73 – 453]	1,200 [476 – 1,633]	165 [107 – 529]	1,899 [916 – 2,235]
no. species ≥50% exposed to 3 EE types	0 [0 – 61]	2 [4 – 48]	5 [4 – 50]	11 [38 – 168]	337 [251 – 1,027]	27 [52 – 236]	737 [432 – 1,478]
no. species ≥75% exposed to 3 EE types	0 [0 – 33]	0 [4 – 28]	2 [1 – 34]	2 [23 – 92]	93 [174 – 624]	1 [34 – 140]	212 [272 – 1,005]

Supplementary Table 7. Ecoregion exposure. Results for ecoregion exposure to different extreme event types calculated as mean across all combinations of climate models and impact models. Number of ecoregions is 794. Listed are absolute values for the respective year (% ecoregion exposed), and in brackets the minimum and maximum of the model ensemble. Mean change % exposed is the difference to the year 2000, where in brackets the minimum is the lowest value for change relative to year 2000 by any ensemble member (and maximum the maximum value). Results also show the mean number of ecoregions with ≥25% area exposed to extreme events (as well as 50% and 75%), calculated by first averaging exposure across all combinations of climate models and impact models for each ecoregion, and then counting ecoregions above the threshold. Minimum and maximum values represent the count of ecoregions exceeding the 25% threshold (and 50% and 75% threshold respectively) in individual model projections. The minimum count can exceed the mean because individual models may show strong exposure in different ecoregions, resulting in fewer ecoregions meeting the threshold when exposures are averaged across all models.

		**SSP1-2.6**		**SSP3-7.0**		**SSP5-8.5**	
	**2000**	**2050**	**2085**	**2050**	**2085**	**2050**	**2085**
**Heatwave**							
% ecoregion exposed	14.0 [11.2 – 21.1]	52.8 [39.4 – 71.9]	55.3 [38.8 – 79.4]	63.8 [53.7 – 83.4]	85.7 [78.5 – 96.4]	67.7 [55.1 – 85.4]	90.5 [84.3 – 97.6]
no. ecoregions ≥25% exposed	116 [84 – 274]	676 [523 – 754]	698 [501 – 765]	759 [645 – 775]	778 [729 – 779]	766 [657 – 776]	779 [761 – 779]
no. ecoregions ≥50% exposed	1 [8 – 59]	424 [251 – 642]	461 [242 – 703]	563 [431 – 735]	763 [660 – 778]	612 [440 – 749]	776 [719 – 779]
no. ecoregions ≥75% exposed	0 [0 – 2]	171 [90 – 428]	196 [92 – 534]	306 [215 – 608]	628 [535 – 770]	345 [234 – 646]	701 [606 – 777]
mean change % ecoregion exposed		38.8 [26.7 – 59.8]	41.3 [26.1 – 67.2]	49.8 [40.9 – 71.3]	71.7 [65.8 – 84.3]	53.7 [42.4 – 73.2]	76.5 [71.6 – 85.4]
**Wildfire**							
% ecoregion exposed	6.9 [5.1 – 9.2]	12.9 [8.8 – 18.3]	13.7 [9.2 – 19.8]	15.8 [11.0 – 22.4]	23.9 [15.4 – 33.7]	17.0 [12.0 – 23.9]	27.3 [17.0 – 38.4]
no. ecoregions ≥25% exposed	0 [0 – 39]	41 [41 – 198]	55 [50 – 230]	130 [71 – 303]	348 [179 – 472]	160 [107 – 330]	428 [206 – 531]
no. ecoregions ≥50% exposed	0 [0 – 3]	0 [2 – 30]	0 [3 – 34]	0 [6 – 58]	26 [48 – 197]	0 [6 – 69]	70 [63 – 257]
no. ecoregions ≥75% exposed	0 [0 – 0]	0 [0 – 4]	0 [0 – 3]	0 [0 – 8]	0 [6 – 33]	0 [0 – 7]	0 [9 – 64]
mean change % ecoregion exposed		6.0 [3.1 – 11.0]	6.7 [3.7 – 12.5]	8.8 [5.5 – 15.1]	17.0 [9.9 – 26.4]	10.0 [5.8 – 16.6]	20.3 [11.5 – 31.2]
**Drought**							
% ecoregion exposed	4.4 [3.1 – 5.5]	5.8 [3.8 – 8.5]	6.1 [3.9 – 9.0]	7.5 [4.5 – 11.0]	12.7 [8.3 – 22.2]	8.4 [5.2 – 13.1]	14.8 [8.8 – 26.0]
no. ecoregions ≥25% exposed	0 [0 – 21]	0 [1 – 59]	2 [2 – 77]	6 [8 – 106]	108 [57 – 266]	19 [8 – 144]	149 [81 – 339]
no. ecoregions ≥50% exposed	0 [0 – 1]	0 [0 – 25]	0 [0 – 30]	0 [0 – 31]	6 [7 – 103]	0 [0 – 30]	18 [10 – 145]
no. ecoregions ≥75% exposed	0 [0 – 0]	0 [0 – 4]	0 [0 – 7]	0 [0 – 7]	0 [0 – 39]	0 [0 – 8]	0 [0 – 54]
mean change % ecoregion exposed		1.4 [0.3 – 4.3]	1.7 [0.1 – 4.8]	3.1 [1.3 – 6.5]	8.3 [3.5 – 18.1]	4.0 [1.9 – 8.9]	10.3 [3.9 – 21.9]
**River Flood**							
% ecoregion exposed	1.6 [1.0 – 2.4]	2.9 [1.8 – 3.6]	3.0 [1.9 – 4.3]	3.2 [1.7 – 4.6]	5.2 [2.4 – 8.2]	3.5 [1.9 – 4.8]	6.4 [2.7 – 9.9]
no. ecoregions ≥25% exposed	0 [0 – 3]	0 [0 – 4]	0 [0 – 8]	1 [0 – 13]	8 [3 – 61]	5 [1 – 18]	20 [3 – 83]
no. ecoregions ≥50% exposed	0 [0 – 0]	0 [0 – 3]	0 [0 – 3]	0 [0 – 3]	0 [1 – 11]	0 [0 – 4]	0 [0 – 20]
no. ecoregions ≥75% exposed	0 [0 – 0]	0 [0 – 0]	0 [0 – 1]	0 [0 – 2]	0 [0 – 2]	0 [0 – 2]	0 [0 – 9]
mean change % ecoregion exposed		1.3 [0.8 – 2.1]	1.4 [0.7 – 2.7]	1.6 [0.7 – 3.1]	3.6 [1.4 – 6.6]	1.9 [0.8 – 3.2]	4.8 [1.7 – 8.3]
**Multiple events**							
% ecoregion exposed 2 EE types	0.6 [0.1 – 1.6]	7.6 [5.3 – 9.8]	9.3 [5.5 – 14.1]	14.2 [11.4 – 18.1]	37.3 [27.7 – 46.9]	17.5 [13.5 – 23.0]	45.2 [33.2 – 57.8]
no. ecoregions ≥25% exposed to 2 EE types	0 [0 – 15]	50 [44 – 118]	75 [47 – 172]	161 [127 – 235]	495 [310 – 524]	223 [157 – 285]	573 [377 – 614]
no. ecoregions ≥50% exposed to 2 EE types	0 [0 – 2]	5 [10 – 32]	7 [16 – 55]	22 [42 – 89]	236 [183 – 361]	44 [62 – 129]	346 [245 – 481]
no. ecoregions ≥75% exposed to 2 EE types	0 [0 – 0]	0 [1 – 9]	1 [1 – 12]	2 [9 – 28]	78 [106 – 219]	4 [18 – 59]	147 [137 – 317]

% ecoregion exposed 3 EE types	0.1 [0.0 – 0.5]	0.4 [0.2 – 0.5]	0.6 [0.3 – 0.9]	1.4 [0.8 – 2.3]	7.9 [4.7 – 12.8]	2.1 [0.8 – 3.5]	11.9 [7.5 – 17.5]
no. ecoregions ≥25% exposed to 3 EE types	0 [0 – 5]	0 [0 – 2]	1 [1 – 6]	1 [4 – 28]	80 [44 – 138]	8 [3 – 39]	129 [82 – 191]
no. ecoregions ≥50% exposed to 3 EE types	0 [0 – 1]	0 [0 – 1]	0 [0 – 2]	0 [1 – 10]	18 [14 – 72]	0 [0 – 14]	46 [30 – 106]
no. ecoregions ≥75% exposed to 3 EE types	0 [0 – 0]	0 [0 – 0]	0 [0 – 1]	0 [0 – 3]	2 [5 – 37]	0 [0 – 7]	10 [8 – 61]

Supplementary Table 8 | Projected exposure of species to combinations of extreme events. Proportion of area within species' range exposed to all possible combinations of extreme events, averaged across all species by taxon for three emission scenarios (SSP1-2.6, SSP3-7.0, SSP5-8.5) and three years (2000, 2050, 2085). For each grid cell, we identified event types with high frequencies (≥0.33 yr^{-1}, corresponding to events occurring at least every third year) and determined which combinations of event types occurred. Values represent the percentage of each species' range exposed to each specific combination of events, then averaged across all species per taxon (7,605 amphibian species, 10,562 bird species, 5,476 mammal species, 10,293 reptile species). Values are plotted in Supplementary Figure 4.

		SSP1-2.6		SSP3-7.0		SSP5-8.5	
	2000	2050	2085	2050	2085	2050	2085
**Amphibian**							
Heatwave – Wildfire	1.2 [0.1 – 5.4]	6.7 [2.6 – 10]	6.8 [3.1 – 9.1]	11.6 [6.5 – 15.6]	30.4 [19.6 – 35.4]	16.3 [10 – 20.9]	38.5 [33.3 – 43.5]
Heatwave – Drought	0.5 [0 – 2.7]	1.3 [0.6 – 2.6]	1.2 [0.7 – 2.1]	3.9 [1.2 – 9.2]	15 [6.3 – 21.8]	5.5 [1.5 – 10]	20.2 [11.2 – 27.6]
Heatwave – Flood	0 [0 – 0]	0.1 [0 – 0.1]	0.1 [0 – 0.3]	0.2 [0 – 0.6]	2.3 [0.1 – 5.3]	0.3 [0 – 1]	3.8 [0.2 – 8.4]
Wildfire – Drought	0.6 [0 – 2.6]	0.9 [0.2 – 2.4]	0.7 [0.3 – 1.9]	2.6 [0.4 – 6.4]	11.4 [4.1 – 17.2]	3.8 [0.7 – 7.7]	16.7 [8.9 – 22.4]
Wildfire – Flood	0 [0 – 0]	0 [0 – 0]	0 [0 – 0]	0 [0 – 0.1]	0.3 [0 – 0.8]	0 [0 – 0.1]	0.5 [0 – 1.2]
Drought – Flood	0 [0 – 0]	0 [0 – 0]	0 [0 – 0]	0 [0 – 0]	0.1 [0 – 0.5]	0 [0 – 0]	0.2 [0 – 0.5]
Heatwave – Wildfire – Drought	0.5 [0 – 2.4]	0.9 [0.2 – 2.4]	0.7 [0.2 – 1.8]	2.5 [0.4 – 6.2]	11.3 [3.9 – 17.2]	3.8 [0.6 – 7.5]	16.7 [8.8 – 22.3]
Heatwave – Wildfire – Flood	0 [0 – 0]	0 [0 – 0]	0 [0 – 0]	0 [0 – 0]	0.3 [0 – 0.8]	0 [0 – 0]	0.5 [0 – 1.2]
Wildfire – Drought – Flood	0 [0 – 0]	0 [0 – 0]	0 [0 – 0]	0 [0 – 0]	0.1 [0 – 0.3]	0 [0 – 0]	0.1 [0 – 0.3]
**Bird**							
Heatwave – Wildfire	0.8 [0.1 – 2.9]	7.1 [3.6 – 9.3]	7.5 [4.4 – 10.3]	11.9 [8.2 – 14.9]	29.5 [21.7 – 35.5]	15.4 [10.9 – 18]	36.1 [31.8 – 42.7]
Heatwave – Drought	0.4 [0 – 1.6]	1.3 [0.5 – 1.9]	1.5 [0.8 – 2.5]	3.6 [1.2 – 8.1]	13 [5.6 – 18.7]	5.2 [1.7 – 9.1]	16.7 [9.6 – 21.9]
Heatwave – Flood	0 [0 – 0]	0.1 [0 – 0.3]	0.3 [0 – 0.8]	0.5 [0 – 1.5]	3.4 [0.4 – 7.5]	0.7 [0.1 – 1.8]	5.3 [0.4 – 10.9]
Wildfire – Drought	0.3 [0 – 1.5]	0.7 [0.1 – 1.5]	0.6 [0.3 – 1.1]	2.3 [0.4 – 5.8]	9.6 [3.3 – 14.6]	3.4 [0.7 – 6.8]	13.2 [7.3 – 17.5]
Wildfire – Flood	0 [0 – 0]	0 [0 – 0]	0 [0 – 0]	0 [0 – 0.1]	0.6 [0.1 – 1.3]	0.1 [0 – 0.1]	1 [0.1 – 2]
Drought – Flood	0 [0 – 0]	0 [0 – 0]	0 [0 – 0]	0 [0 – 0]	0.2 [0 – 0.5]	0 [0 – 0]	0.3 [0 – 0.7]
Heatwave – Wildfire – Drought	0.3 [0 – 1.3]	0.7 [0.1 – 1.4]	0.6 [0.3 – 1]	2.2 [0.3 – 5.6]	9.5 [3.2 – 14.5]	3.3 [0.6 – 6.7]	13.1 [7.2 – 17.4]
Heatwave – Wildfire – Flood	0 [0 – 0]	0 [0 – 0]	0 [0 – 0]	0 [0 – 0.1]	0.6 [0.1 – 1.3]	0.1 [0 – 0.1]	1 [0.1 – 2]
Wildfire – Drought – Flood	0 [0 – 0]	0 [0 – 0]	0 [0 – 0]	0 [0 – 0]	0.1 [0 – 0.3]	0 [0 – 0]	0.2 [0 – 0.5]
**Mammal**							
Heatwave – Wildfire	0.7 [0.1 – 2.6]	7.4 [4.4 – 9.2]	7.9 [4.8 – 10.6]	12.6 [10.2 – 13.8]	30.3 [24.1 – 36]	15.9 [11.8 – 17.9]	36.7 [31.1 – 43.6]
Heatwave – Drought	0.3 [0 – 1.3]	1.2 [0.6 – 1.7]	1.4 [0.9 – 2.1]	3.3 [1.6 – 6.7]	11.9 [5.9 – 16.9]	4.6 [1.9 – 7.4]	15.4 [9.5 – 20.3]
Heatwave – Flood	0 [0 – 0]	0.1 [0 – 0.3]	0.2 [0 – 0.7]	0.5 [0 – 1.5]	3.4 [0.3 – 7.8]	0.7 [0.1 – 1.9]	5.3 [0.4 – 10.8]
Wildfire – Drought	0.3 [0 – 1.3]	0.6 [0.2 – 1.3]	0.6 [0.3 – 1]	2.1 [0.7 – 4.7]	8.7 [3.7 – 12.4]	3 [0.8 – 5.4]	11.9 [7 – 16]
Wildfire – Flood	0 [0 – 0]	0 [0 – 0]	0 [0 – 0]	0.1 [0 – 0.1]	0.6 [0.1 – 1]	0.1 [0 – 0.1]	1 [0.1 – 2.1]
Drought – Flood	0 [0 – 0]	0 [0 – 0]	0 [0 – 0]	0 [0 – 0]	0.1 [0 – 0.4]	0 [0 – 0]	0.3 [0 – 0.7]
Heatwave – Wildfire – Drought	0.2 [0 – 1]	0.6 [0.1 – 1.2]	0.5 [0.3 – 1]	2 [0.6 – 4.5]	8.6 [3.6 – 12.4]	2.9 [0.7 – 5.3]	11.9 [6.9 – 16]
Heatwave – Wildfire – Flood	0 [0 – 0]	0 [0 – 0]	0 [0 – 0]	0 [0 – 0.1]	0.5 [0.1 – 1]	0.1 [0 – 0.1]	1 [0.1 – 2.1]
Wildfire – Drought – Flood	0 [0 – 0]	0 [0 – 0]	0 [0 – 0]	0 [0 – 0]	0.1 [0 – 0.3]	0 [0 – 0]	0.2 [0 – 0.5]
**Reptile**							
Heatwave – Wildfire	0.8 [0 – 2.8]	7.3 [5 – 9.3]	8.4 [5.7 – 10.6]	12.8 [10.5 – 14.1]	31 [25 – 34.9]	15.9 [12.1 – 18.4]	37.7 [30.6 – 44.1]

Heatwave – Drought	0.4 [0 – 1.4]	1.2 [1 – 1.5]	1.5 [1 – 1.9]	3 [1.7 – 5.3]	11.5 [6.5 – 15.4]	4.3 [2.2 – 6.1]	15 [9.2 – 18.9]
Heatwave – Flood	0 [0 – 0]	0.1 [0 – 0.2]	0.1 [0 – 0.4]	0.3 [0 – 1.1]	2.4 [0.3 – 5.3]	0.4 [0 – 1.3]	3.8 [0.4 – 8]
Wildfire – Drought	0.3 [0 – 1.3]	0.5 [0.3 – 1.2]	0.6 [0.2 – 1.2]	1.8 [0.8 – 3.3]	8.4 [4 – 11.5]	2.6 [1.1 – 3.8]	11.8 [6.7 – 15.8]
Wildfire – Flood	0 [0 – 0]	0 [0 – 0.1]	0 [0 – 0]	0 [0 – 0.1]	0.4 [0 – 0.8]	0 [0 – 0.1]	0.8 [0.1 – 2.2]
Drought – Flood	0 [0 – 0]	0 [0 – 0]	0 [0 – 0]	0 [0 – 0]	0.1 [0 – 0.2]	0 [0 – 0]	0.2 [0 – 0.5]
Heatwave – Wildfire – Drought	0.2 [0 – 1.2]	0.5 [0.2 – 1.1]	0.6 [0.2 – 1.2]	1.7 [0.7 – 3.2]	8.3 [3.9 – 11.5]	2.5 [1 – 3.7]	11.7 [6.6 – 15.8]
Heatwave – Wildfire – Flood	0 [0 – 0]	0 [0 – 0.1]	0 [0 – 0]	0 [0 – 0.1]	0.4 [0 – 0.8]	0 [0 – 0.1]	0.8 [0.1 – 2.2]
Wildfire – Drought – Flood	0 [0 – 0]	0 [0 – 0]	0 [0 – 0]	0 [0 – 0]	0.1 [0 – 0.2]	0 [0 – 0]	0.1 [0 – 0.4]

Supplementary Table 9. | Documented examples of species' sensitivity to different extreme event types for terrestrial vertebrates.

Extreme event	Amphibian	Bird	Mammal	Reptile
Heatwave	positive:<br>• reduced infection with heat sensitive chytrid fungi (Beukema *et al* 2021)<br>negative:<br>• thermal stress (Rollins-Smith and Le Sage 2023)<br>• increased evaporative water loss (Rollins-Smith and Le Sage 2023)<br>• reduction in diversity of gut and skin microbiome (Rollins-Smith and Le Sage 2023)<br>• reduced survival of tadpoles (Weerathunga and Rajapaksa 2020), decline in fecundity (Maxwell *et al* 2019)	negative:<br>• dehydration and hyperthermia, leading to decline in physical condition or mortality (Nelson *et al* 2024, Sharpe *et al* 2019)<br>• nest abandonment (Sharpe *et al* 2019), decline in sperm quality (Hurley *et al* 2018) and reproductive success (Regan and Sheldon 2023)<br>• population decline (Maxwell *et al* 2019, Ding *et al* 2024)<br>• reduction in foraging activity and foraging closer to water sources (Funghi *et al* 2019)	negative:<br>• heat stress and dehydration, leading to mortality (Mo *et al* 2021)<br>• abandonment of offspring (Mo *et al* 2021)<br>• decline in body condition (Trondrud *et al* 2023) and fecundity (Maxwell *et al* 2019)<br>• population decline (Maxwell *et al* 2019)<br>• alteration of activity patterns (Stiegler *et al* 2023), reduction in foraging time (Trondrud *et al* 2023)	ambiguous:<br>• change in female-male ratio of embryos (Breitenbach *et al* 2020)<br>negative:<br>• reduced survival of eggs (Hall and Warner 2018), hatchlings (Dayananda and Webb 2017), and adults (Zhang *et al* 2023)<br>• oxidative stress (Zhang *et al* 2023)<br>• lower growth rate (Zhang *et al* 2023)
Wildfire	positive:<br>• habitat creation for species adapted to disturbance (Hossack and Pilliod 2011)<br>negative:<br>• injury and death by flames, smoke and heat (Tomas *et al* 2021)<br>• increase in predation risk after fire (Beranek *et al* 2023)<br>• reduction in water quality leading to slower development and smaller size of tadpoles (McDonald *et al* 2018)<br>• increased breeding dispersal (Barrile *et al* 2022)<br>• decrease in body growth (Barrile *et al* 2022)	positive:<br>• creation of new habitat for species using dead trees, e.g., for nesting cavities (Stillman *et al* 2019)<br>negative:<br>• injury and death by flames, smoke and heat (Tomas *et al* 2021)<br>• smoke leading to reduced body mass and activity (Nihei *et al* 2024)<br>• disruption of migration, e.g. due to dense smoke (Overton *et al* 2022)<br>• loss of nesting sites, e.g., tree collapse (Stojanovic *et al* 2016)	positive:<br>• reduced predation in burned areas (Sokos *et al* 2016)<br>• reduction in vegetation cover and increase in insects can improve foraging (Low *et al* 2024)<br>negative:<br>• injury and death by flames, smoke and heat (Tomas *et al* 2021)<br>• change in foraging behaviour and increase in predation risk (Morandini *et al* 2023)<br>• reduction in food availability (Ancillotto *et al* 2021)<br>• decline in body condition (Ancillotto *et al* 2021)<br>• decline in fecundity (Ancillotto *et al* 2021)	positive:<br>• reduction in vegetation cover can benefit species preferring open habitat (Santos *et al* 2025)<br>• increase in introduced species (Linley *et al* 2024)<br>negative:<br>• injury and death by flames, smoke and heat (Tomas *et al* 2021)<br>• reduction in food sources and shelter leading to lower survival (Santos *et al* 2022)<br>• habitat fragmentation hindering dispersal (Santos *et al* 2022)<br>• destruction of eggs and offspring reducing reproductive success (Santos *et al* 2022)

Drought	<u>positive</u>:<br>• increased persistence and colonization rates for certain species (Davis *et al* 2017)<br>• improved body condition (Maxwell *et al* 2019)<br><u>negative</u>:<br>• increased susceptibility to fungal infection (McDevitt-Galles *et al* 2022)<br>• reduced body condition (Maxwell *et al* 2019, Macdonald *et al* 2023)<br>• loss of breeding sites (Scheele *et al* 2012)<br>• decline in larval density (McDevitt-Galles *et al* 2022)<br>• reduction in fecundity and survival, leading to population decline (Maxwell *et al* 2019, Cayuela *et al* 2016)<br>• (temporal) decline in no. of occupied sites (Scheele *et al* 2012, McDevitt-Galles *et al* 2022)	<u>positive:</u><br>• in wetter regions, increased nesting success under drought (Maxwell *et al* 2019, Nelson *et al* 2024)<br>• increase in abundance (Maxwell *et al* 2019, Prugh *et al* 2018)<br><u>ambiguous</u>:<br>• distribute to other areas to avoid poor conditions (Dobson *et al* 2024)<br><u>negative</u>:<br>• (temporal) reduction in habitat for wetland birds (Barbaree *et al* 2020)<br>• decline in nest success (Maxwell *et al* 2019, Nelson *et al* 2024)<br>• increased mortality (Maxwell *et al* 2019, Nelson *et al* 2024)<br>• population decline (Maxwell *et al* 2019, Nelson *et al* 2024)<br>• increased use of artificial water sources can lead to spread of lethal disease (Rogers *et al* 2018)	<u>positive:</u><br>• population increase (Maxwell *et al* 2019, Prugh *et al* 2018)<br><u>ambiguous</u>:<br>• move to more suitable areas (Maxwell *et al* 2019, Abraham *et al* 2019)<br><u>negative</u>:<br>• decline in body condition (Maxwell *et al* 2019, Watter *et al* 2019, Wells *et al* 2022)<br>• decline in reproductive success (Maxwell *et al* 2019, Wells *et al* 2022)<br>• increased mortality (Maxwell *et al* 2019, Watter *et al* 2019)<br>• (temporal) population decline (Maxwell *et al* 2019, Prugh *et al* 2018, Gandiwa *et al* 2016)	<u>positive</u>:<br>• increase in abundance (Prugh *et al* 2018)<br><u>ambiguous:</u><br>• change in species assemblages (Maxwell *et al* 2019, Dundas *et al* 2021)<br><u>negative</u>:<br>• reduced prey availability (Smith *et al* 2019, Martín *et al* 2023)<br>• reduction in number of offsprings (Smith *et al* 2019)<br>• reduced body condition (Macdonald *et al* 2023, Sperry and Weatherhead 2008)<br>• decline in abundance (Maxwell *et al* 2019, Prugh *et al* 2018)<br>• increased mortality and local (temporal) extirpation (Maxwell *et al* 2019, Westphal *et al* 2016)
River flood	<u>negative</u>:<br>• habitat destruction, esp. species that live under ground (Dayrell *et al* 2024)<br>• increased mortality through higher predation or sometimes drowning (Blaustein *et al* 2010)<br>• disruption of breeding due to mortality of eggs and tadpoles, leading to population declines (Maxwell *et al* 2019)	<u>positive</u>:<br>• population increase and increase in fecundity, e.g., through creation of new (temporary) habitats (e.g., wetlands, marshes) and feeding opportunities (aquatic prey) (Maxwell *et al* 2019)<br><u>negative</u>:<br>• decrease in body condition (Wilson and Peach 2006) and fecundity (Maxwell *et al* 2019)<br>• destruction of nests leading to loss of eggs and nestlings (Elas *et al* 2023)	<u>positive</u>:<br>• population increase for aquatic species, e.g. due to higher prey abundance (Bodmer *et al* 2018)<br><u>negative</u>:<br>• injury and drowning (Bodmer *et al* 2018)<br>• displacement leading to stress and crowding in refuge areas resulting in higher competition and predation (Bodmer *et al* 2018)<br>• increase prevalence of water-borne diseases, e.g.,	<u>positive</u>:<br>• increase in prey availability, e.g., arthropods (Grimm-Seyfarth *et al* 2018)<br><u>negative</u>:<br>• mortality (Crowley and Preece 2019)<br>• reduced prey availability, e.g., mammal (Ujvari *et al* 2016)<br>• displacement (Chowdhury *et al* 2022)<br>• habitat destruction (Chowdhury *et al* 2022)

	• local extirpation and population decline (Maxwell *et al* 2019, Wilson and Peach 2006)	leptospirosis (Ashraf *et al* 2017)<br>• local extirpation and population decline (Maxwell *et al* 2019)	

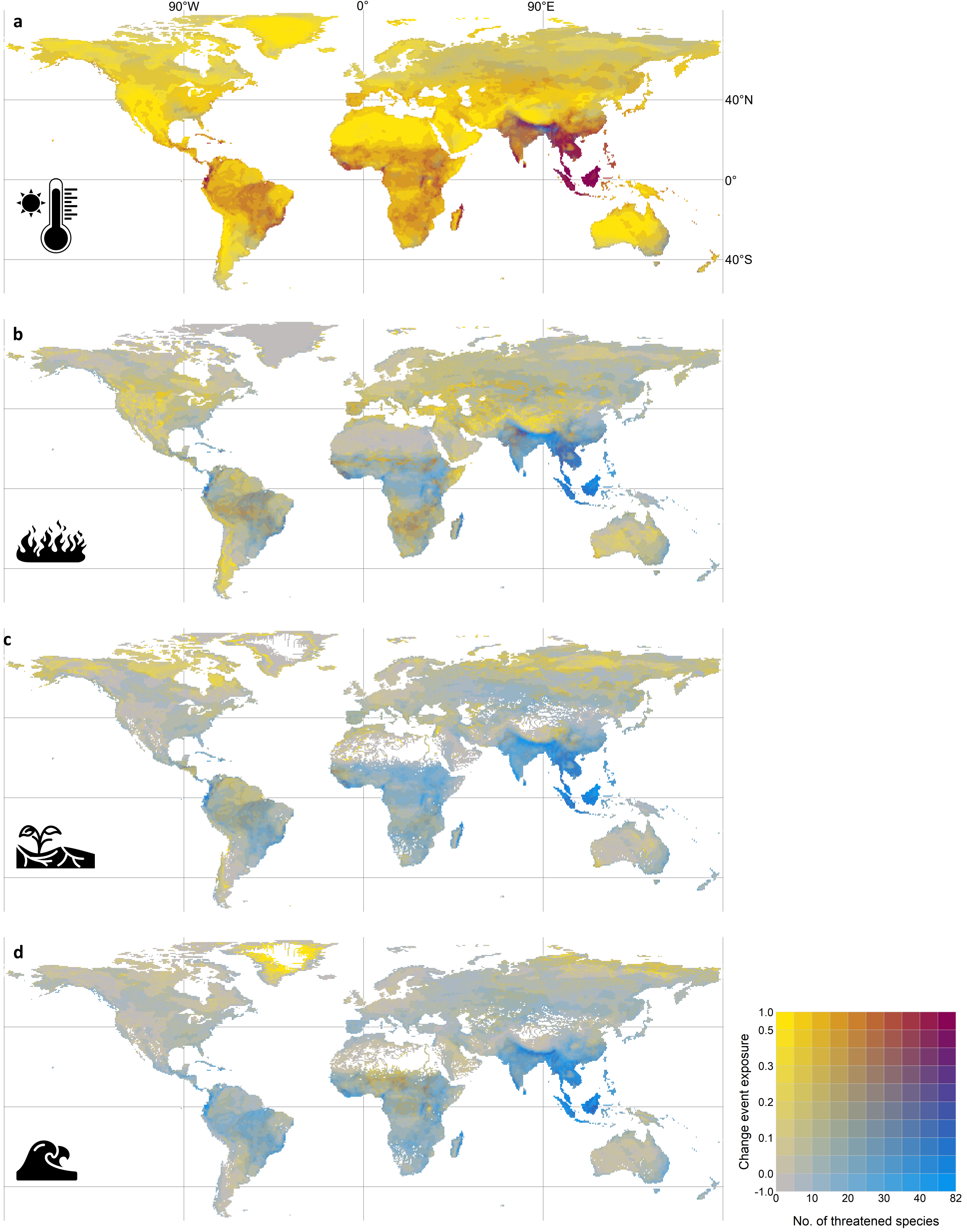

Supplementary Fig. 1 | Exposure of combined species richness for threatened species to projected change in extreme event occurrences. Change in annual frequency of extreme event from 2000 to 2050 for SSP3−7.0 for (a) heatwave, (b) wildfire, (c) drought, (d) river flood for all four taxa combined based on species richness data from the IUCN Red List of Threatened Species.

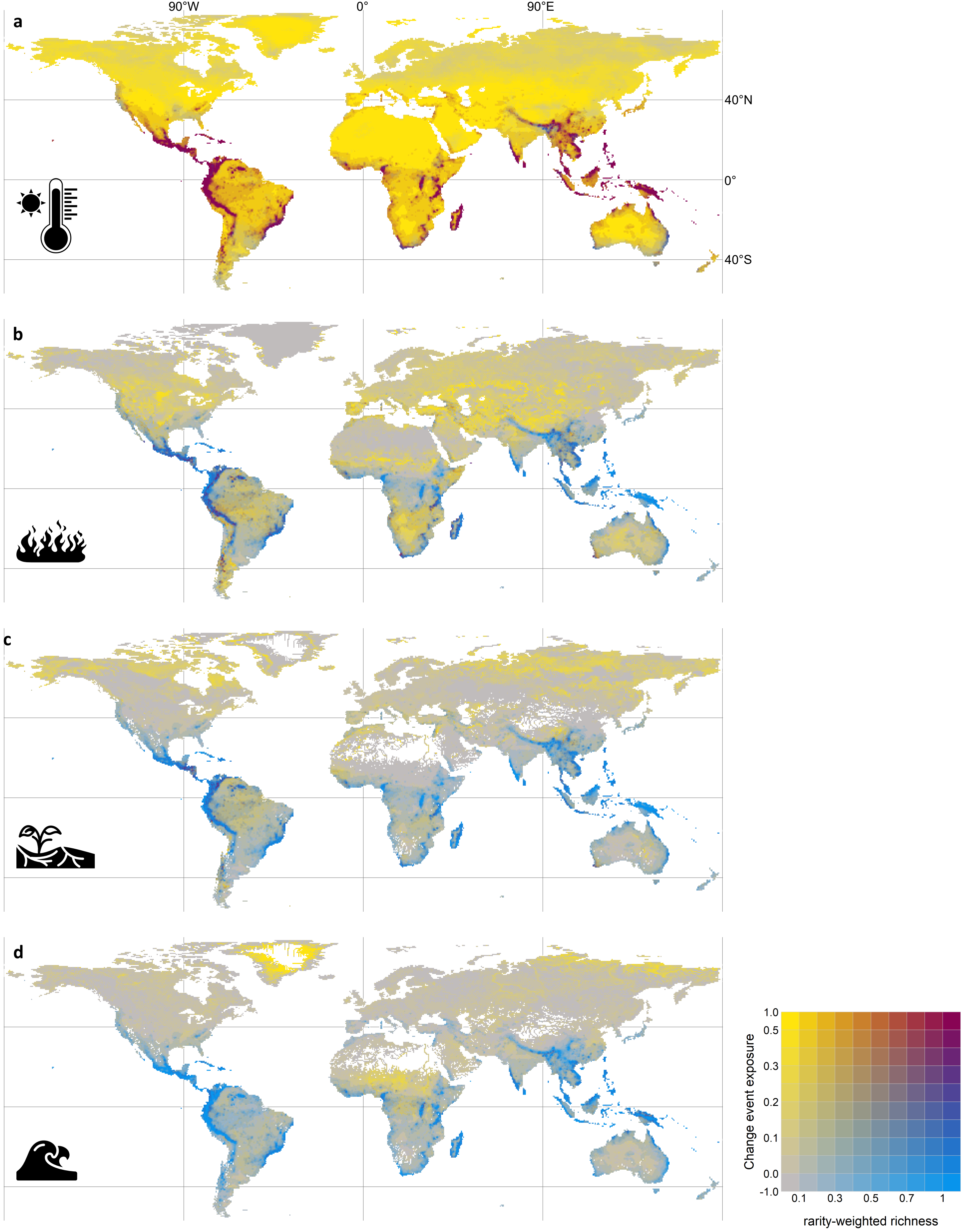

Supplementary Fig. 2 | Exposure to projected change in extreme event occurrences for rarity-weighted richness. Change in annual frequency of extreme event from 2000 to 2050 for SSP3−7.0 for (a) heatwave, (b) wildfire, (c) drought, (d) river flood for all four taxa combined for rarity-weighted species richness data from the IUCN Red List of Threatened Species.

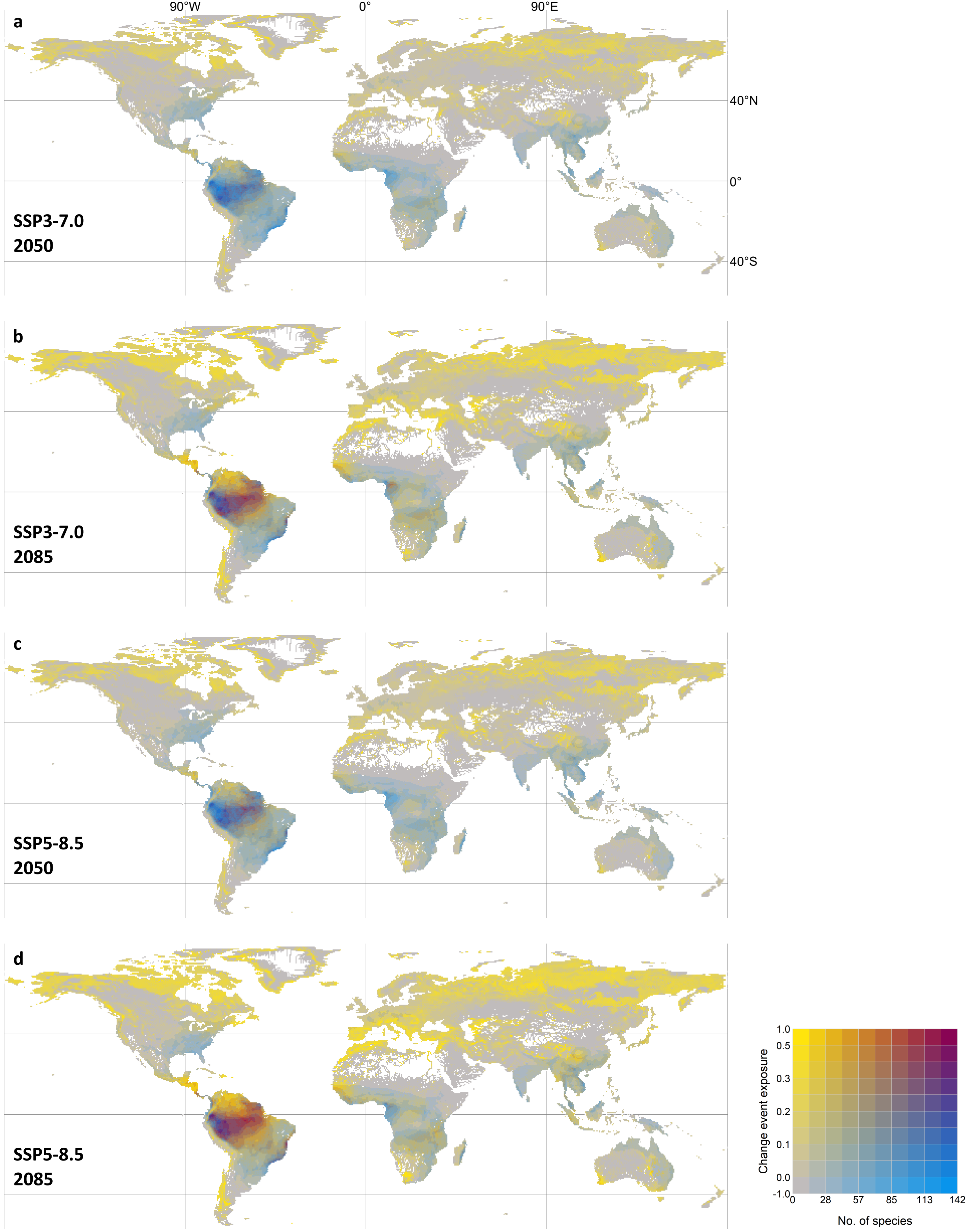

Supplementary Fig. 3 | Exposure to projected change in drought occurrences for amphibian species richness. Change in annual frequency of droughts from 2000 to (a) 2050 for SSP3−7.0, (b) 2085 for SSP3−7.0, (c) 2050 for SSP5−8.5, and (d) 2085 for SSP5−8.5

.

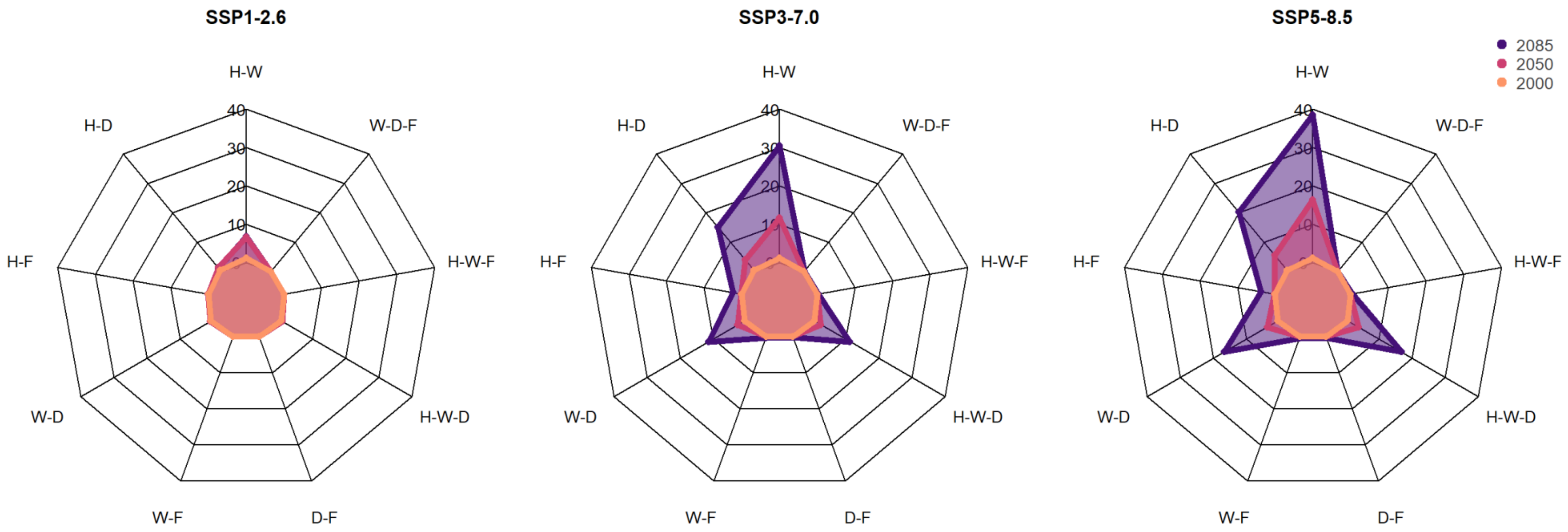

Supplementary Fig. 4a | Projected exposure of amphibians to combinations of extreme events. Proportion of area within species' range exposed to all possible combinations of extreme events, averaged across all amphibian species for three emission scenarios (SSP1-2.6, SSP3-7.0, SSP5-8.5) and three years (2000, 2050, 2085). For each grid cell, we identified event types with high frequencies (≥0.33 yr^{-1}, corresponding to events occurring at least every third year) and determined which combinations of event types occurred at this high frequency. Values represent the percentage of each species' range exposed to each specific combination of events, then averaged across all amphibian species (n=7,605). D: drought, F: river flood, H: heatwave, W: wildfire, where H-W stands for the combination heatwave-wildfire, etc. Values used for plotting are in Supplementary Table 8.

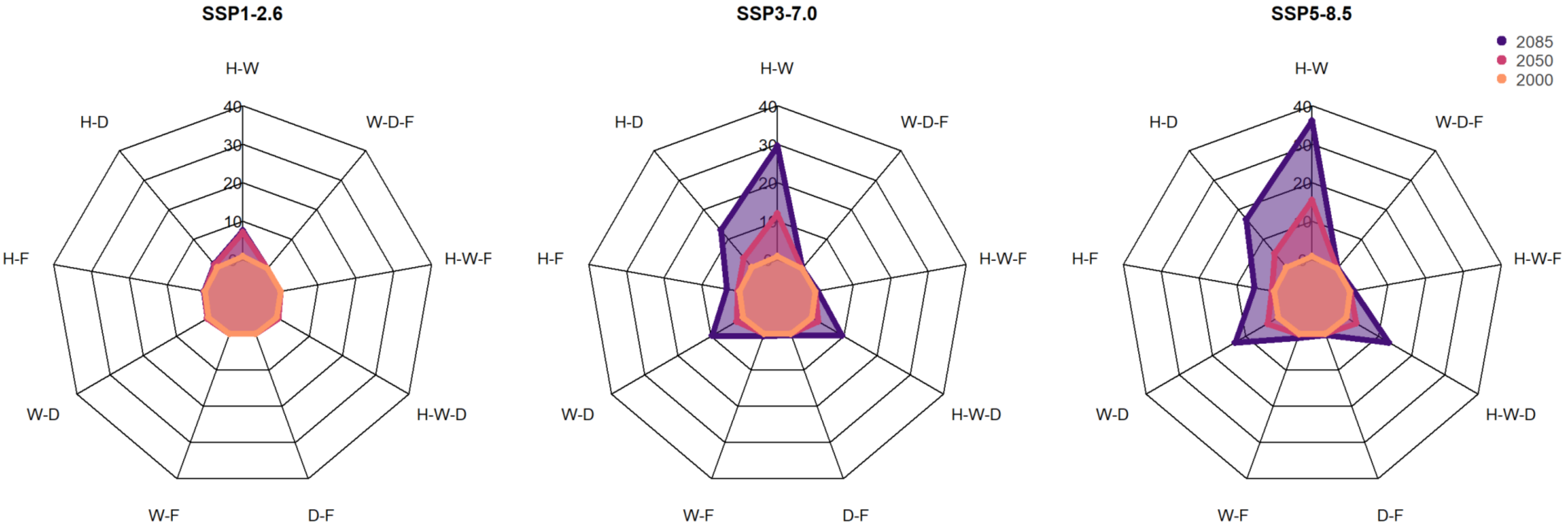

Supplementary Fig. 4b | Projected exposure of birds (n=10,562 bird species) to combinations of extreme events.

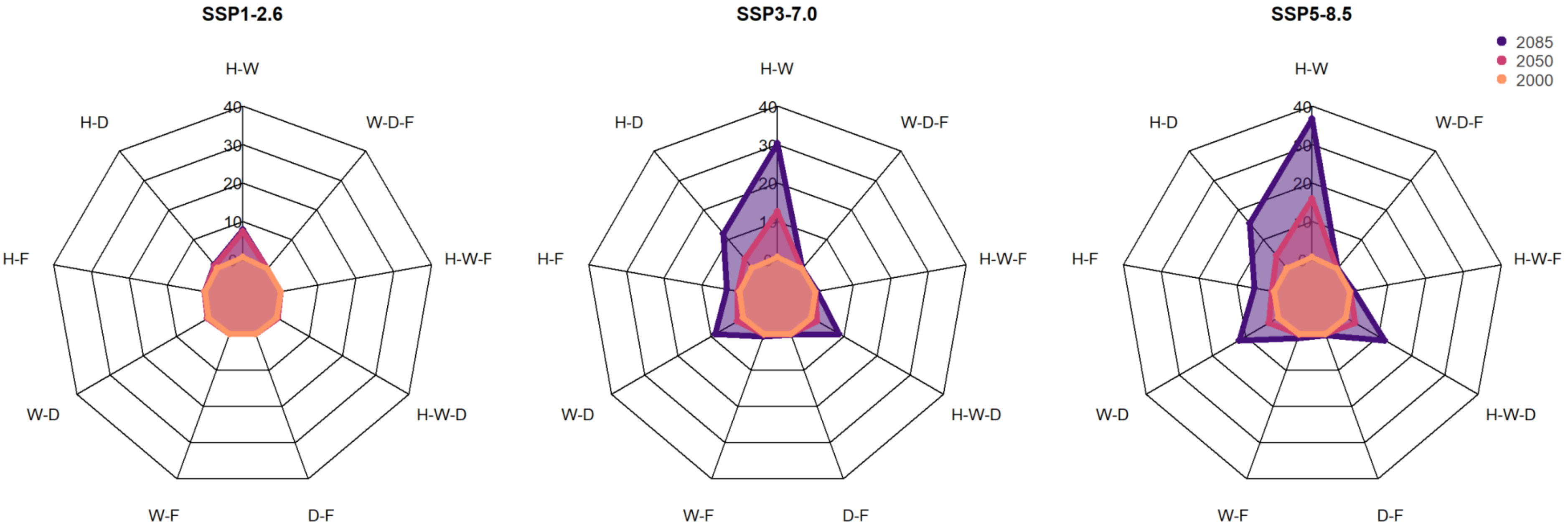

Supplementary Fig. 4c | Projected exposure of mammals (n=5,476 mammal species) to combinations of extreme events.

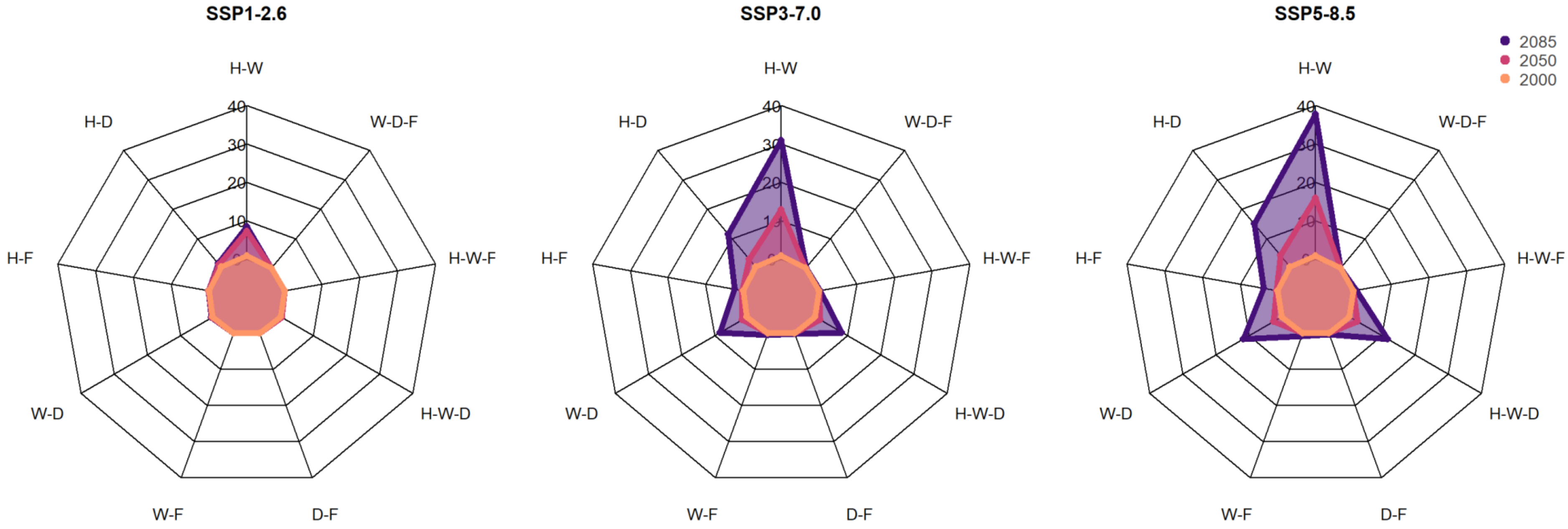

Supplementary Fig. 4d | Projected exposure of reptiles (n=10,293 reptile species) to combinations of extreme events.

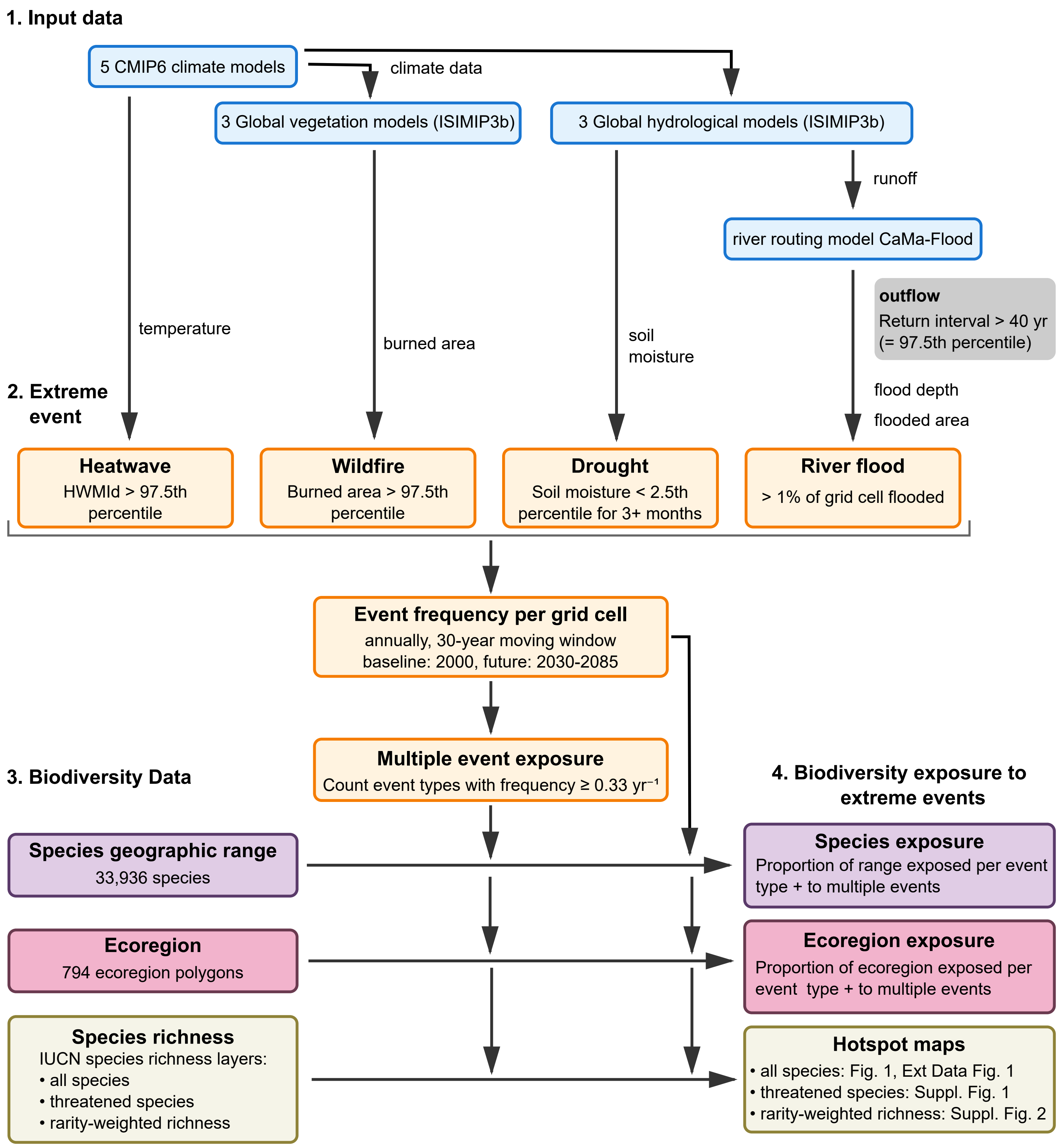

Supplementary Fig. 5 | Conceptual framework of the analytical workflow. Schematic overview showing the main steps of the analysis, from models (blue boxes) and the output variables, through extreme event definition (orange boxes) to calculation of species and ecoregion exposure metrics (purple boxes).

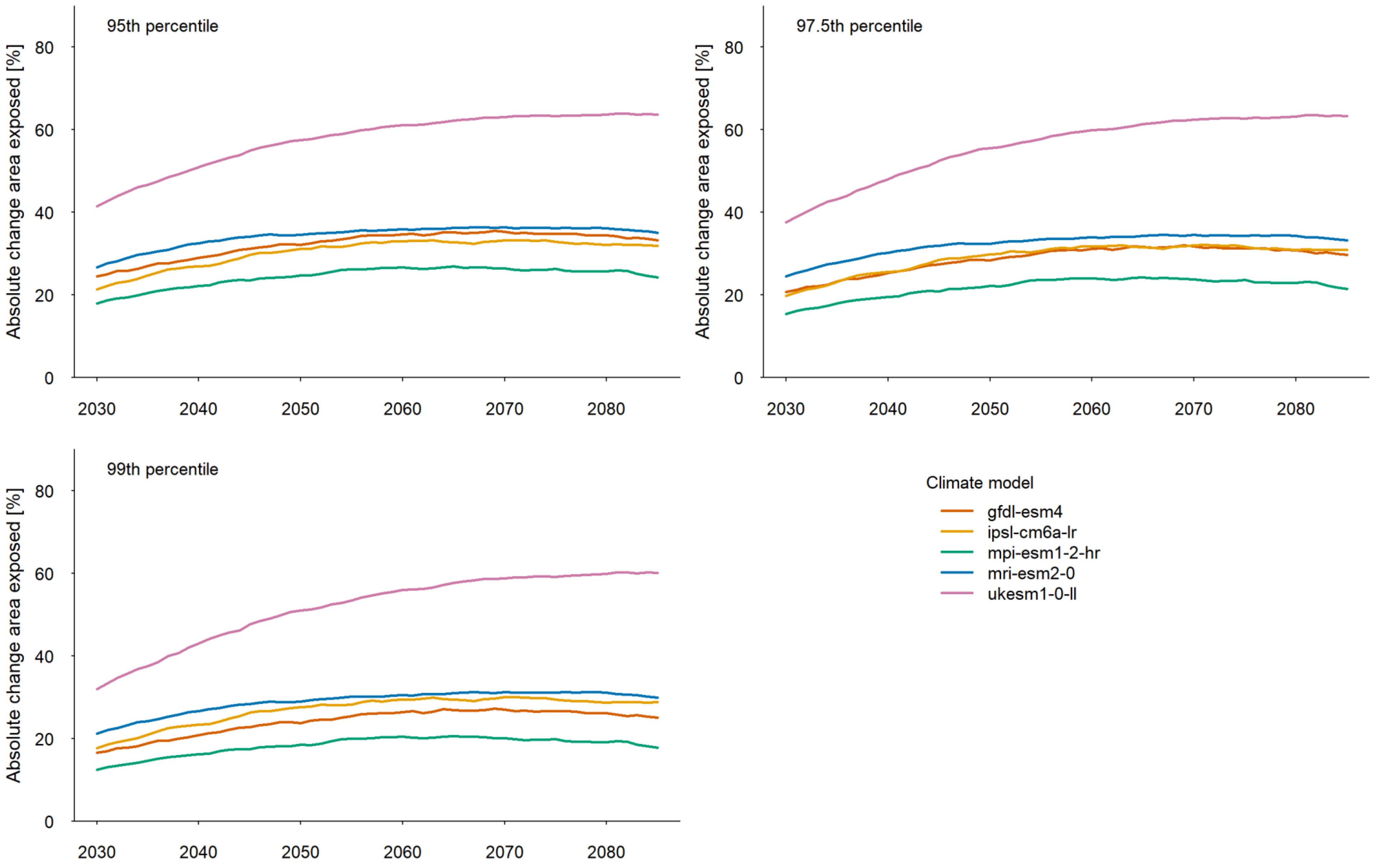

Supplementary Fig. 6a | Sensitivity analysis for threshold used to define heatwaves for SSP1-2.6. In the main analysis, we used a threshold of 97.5th percentile to define heatwaves. Plotted is the proportion of area globally exposed to heatwaves using three different thresholds: 95th, 97.5th and 99th percentile. The figure shows that the variability due to climate model is much larger than the variability due to percentile threshold.

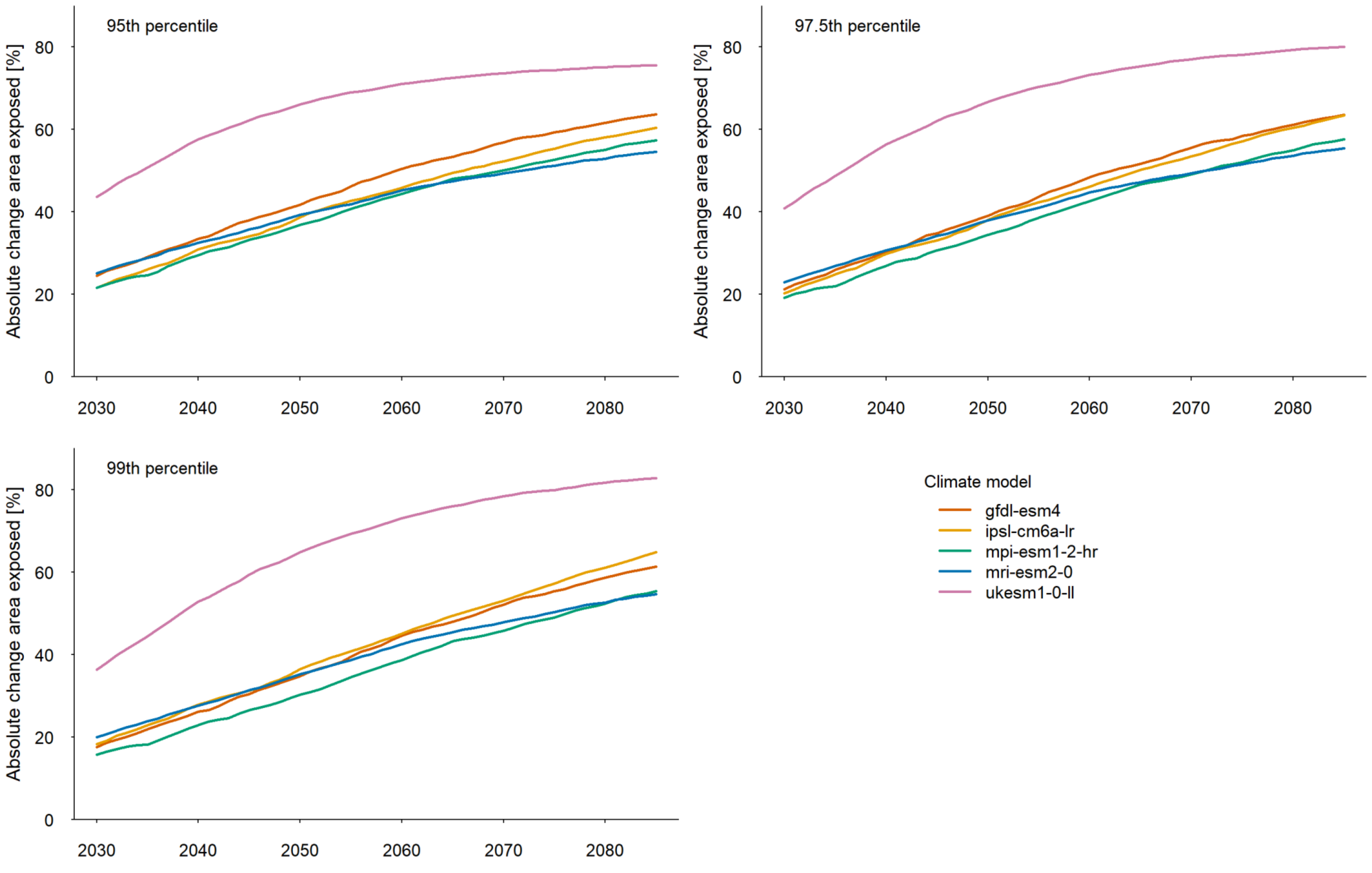

Supplementary Fig. 6b | Sensitivity analysis for threshold used to define heatwaves for SSP3-7.0.

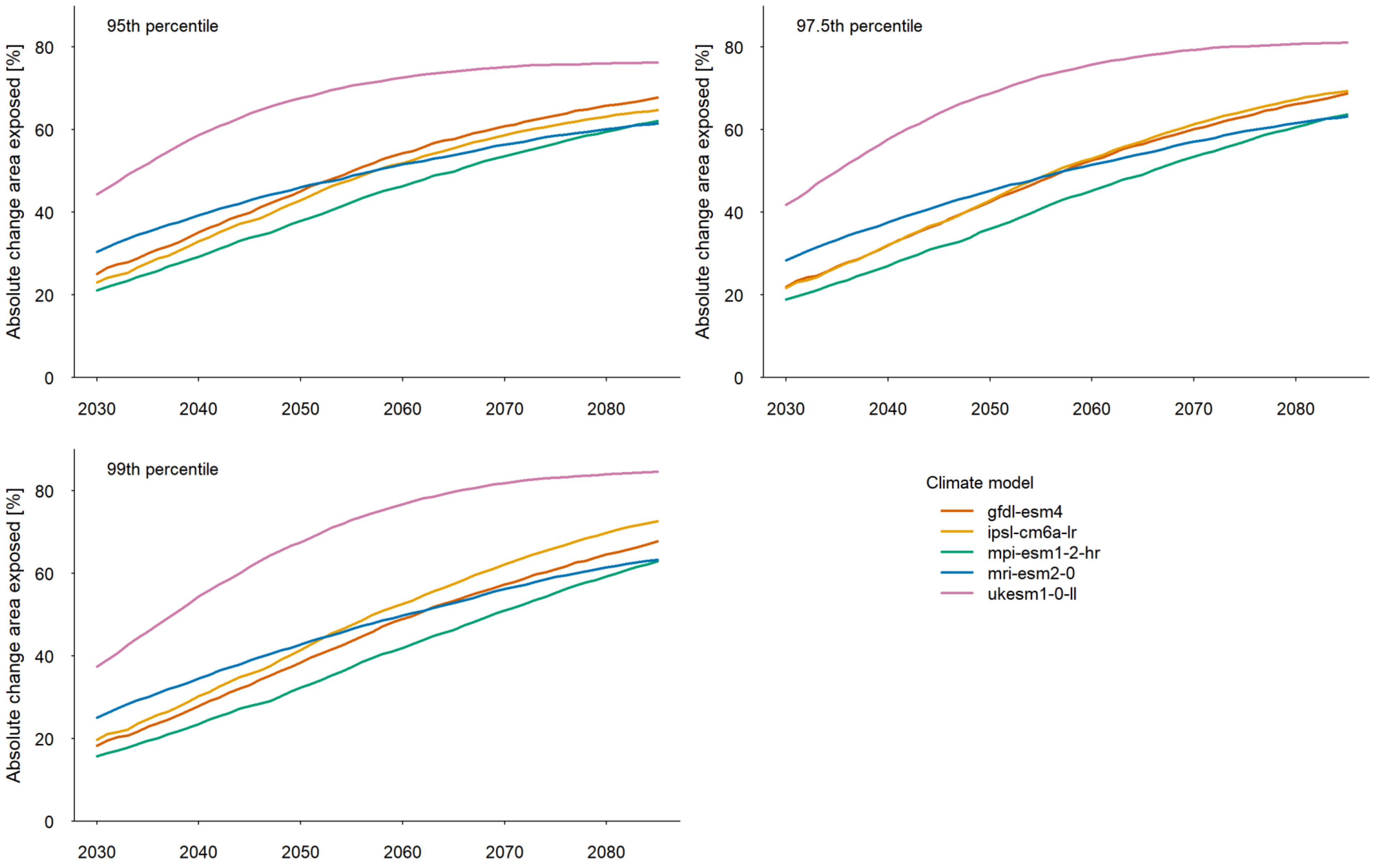

Supplementary Fig. 6c | Sensitivity analysis for threshold used to define heatwaves for SSP5-8.5.

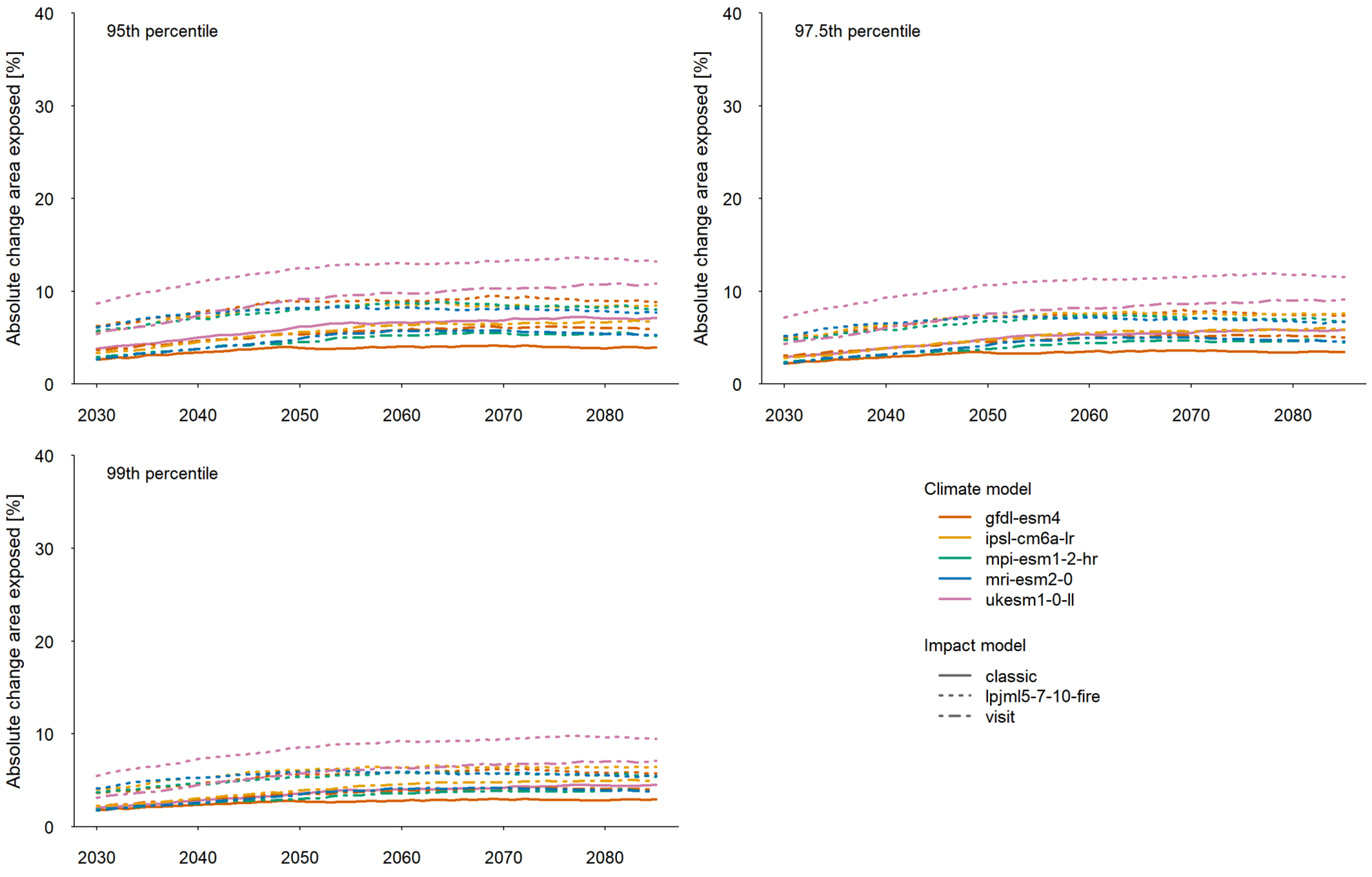

Supplementary Fig. 7a | Sensitivity analysis for threshold used to define wildfires for SSP1-2.6. In the main analysis, we used a threshold of 97.5th percentile to define wildfires. Plotted is the proportion of area globally exposed to wildfires using three different thresholds: 95th, 97.5th and 99th percentile. The figure shows that the variability due to climate model and impact model is much larger than the variability due to percentile threshold.

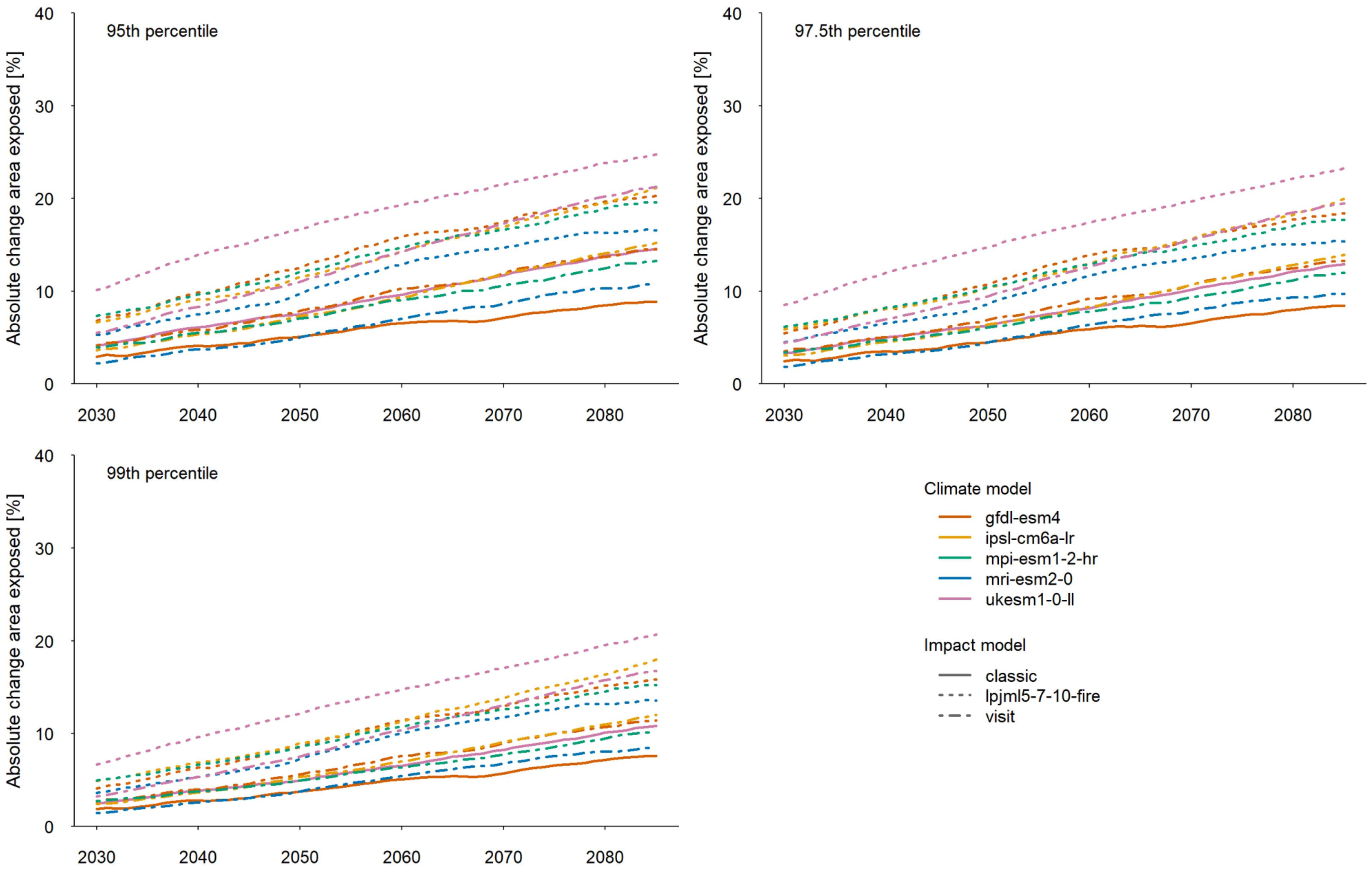

Supplementary Fig. 7b | Sensitivity analysis for threshold used to define wildfires for SSP3-7.0.

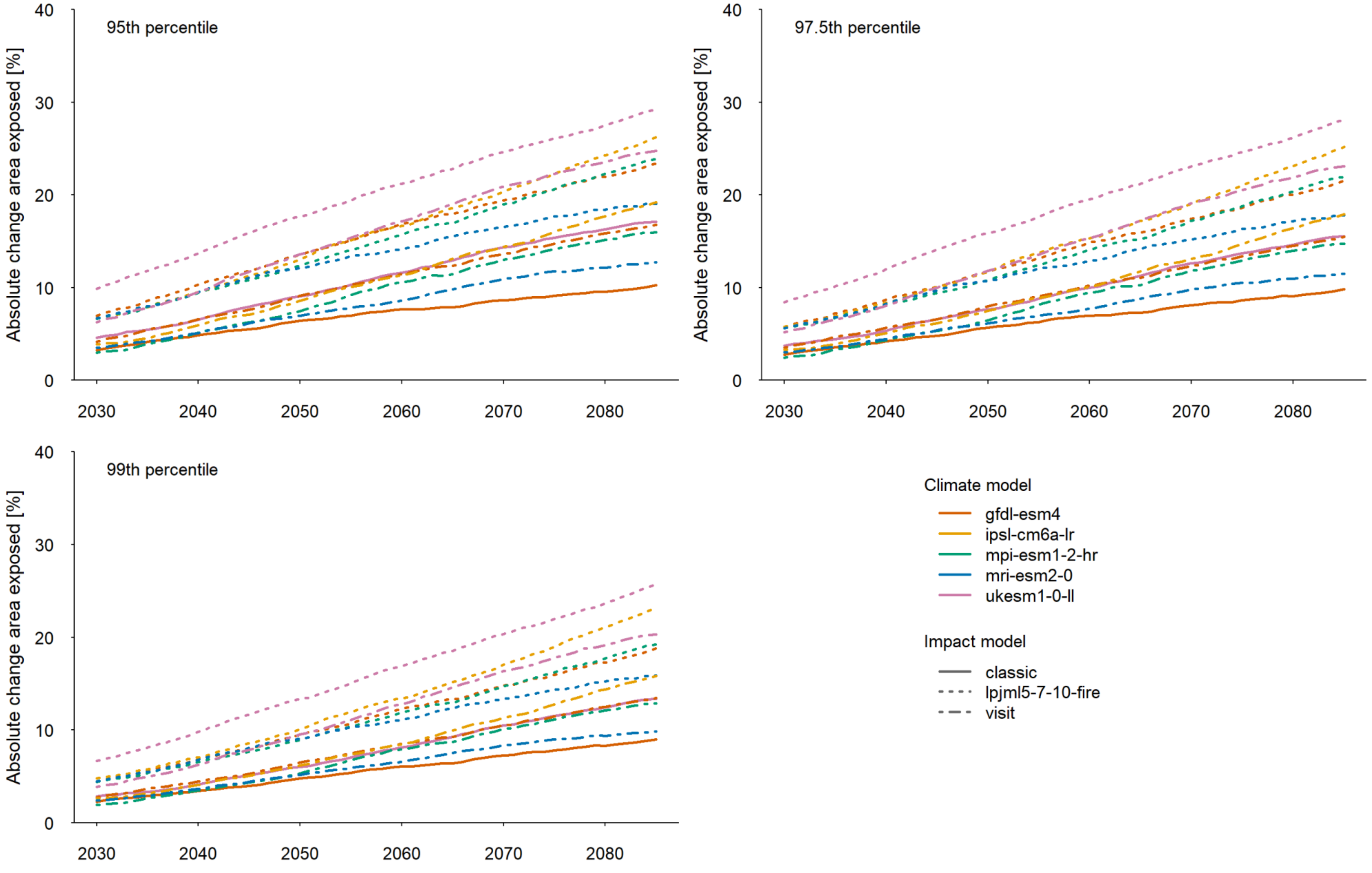

Supplementary Fig. 7c | Sensitivity analysis for threshold used to define wildfires for SSP5-8.5.

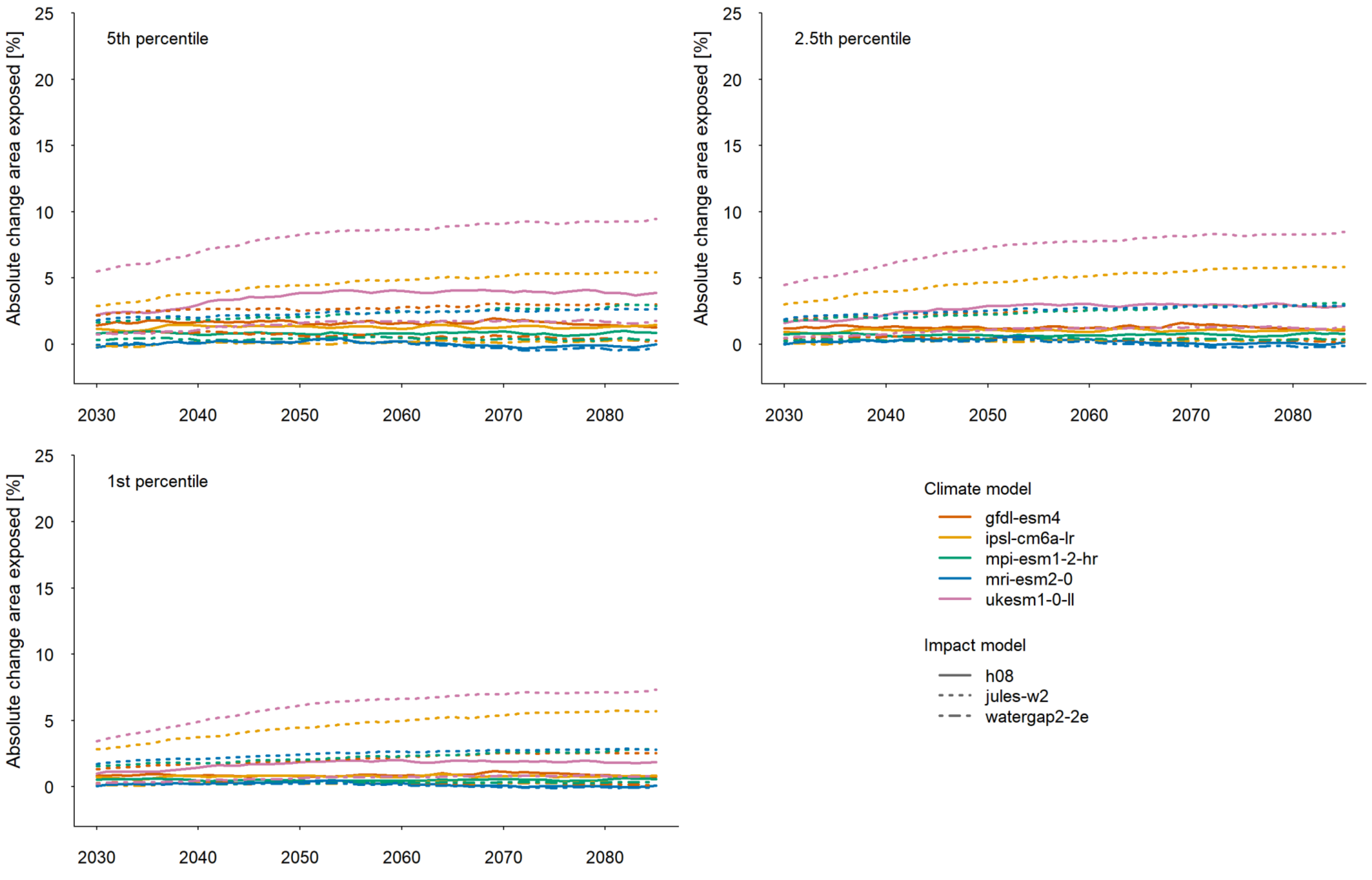

Supplementary Fig. 8a | Sensitivity analysis for threshold used to define droughts for SSP1-2.6. In the main analysis, we used a threshold of 2.5th percentile to define droughts. Plotted is the proportion of area globally exposed to droughts using three different thresholds: 5th, 2.5th and 1st percentile. The figure shows that the variability due to climate model and impact model is much larger than the variability due to percentile threshold.

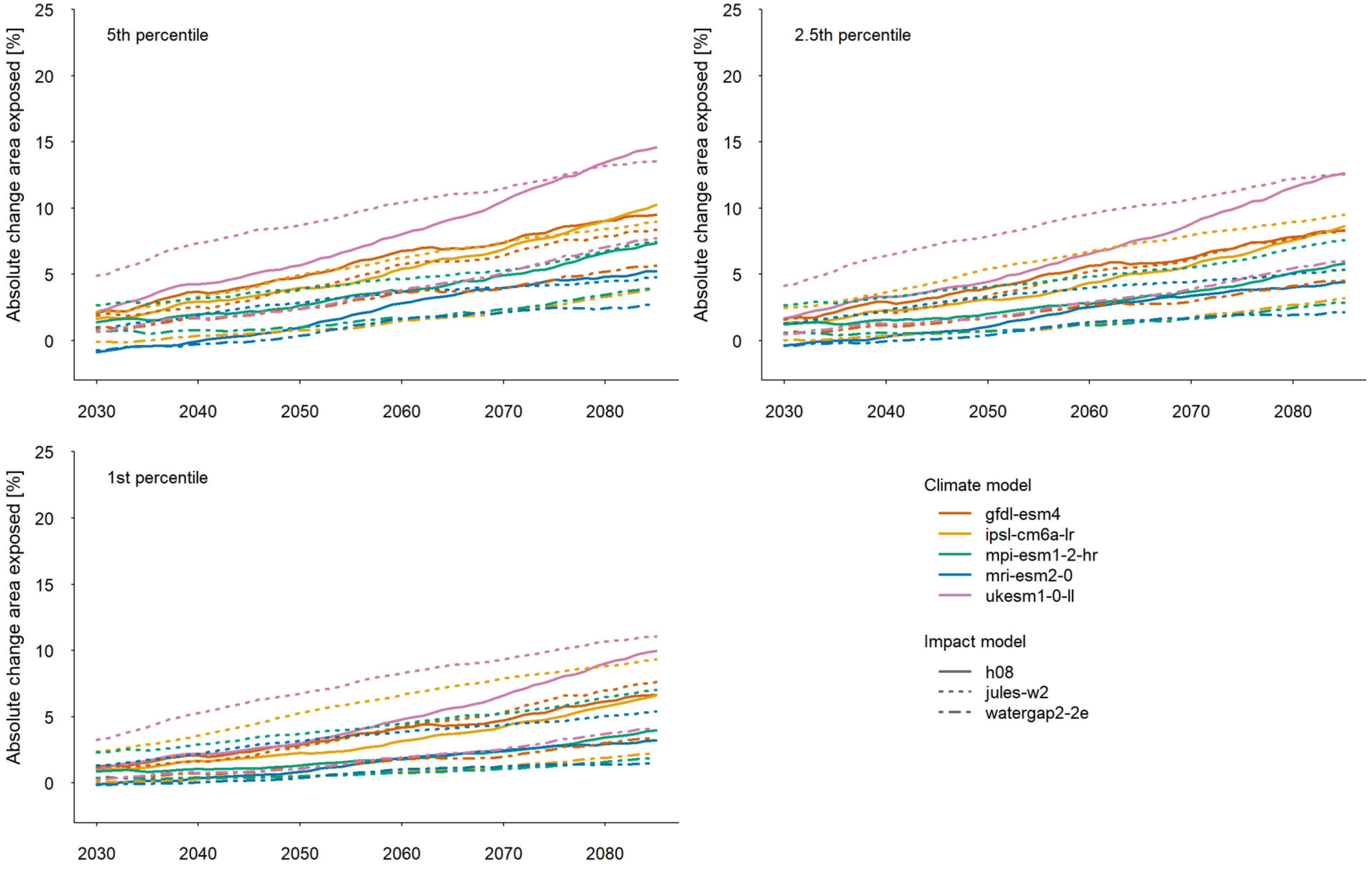

Supplementary Fig. 8b | Sensitivity analysis for threshold used to define droughts for SSP3-7.0.

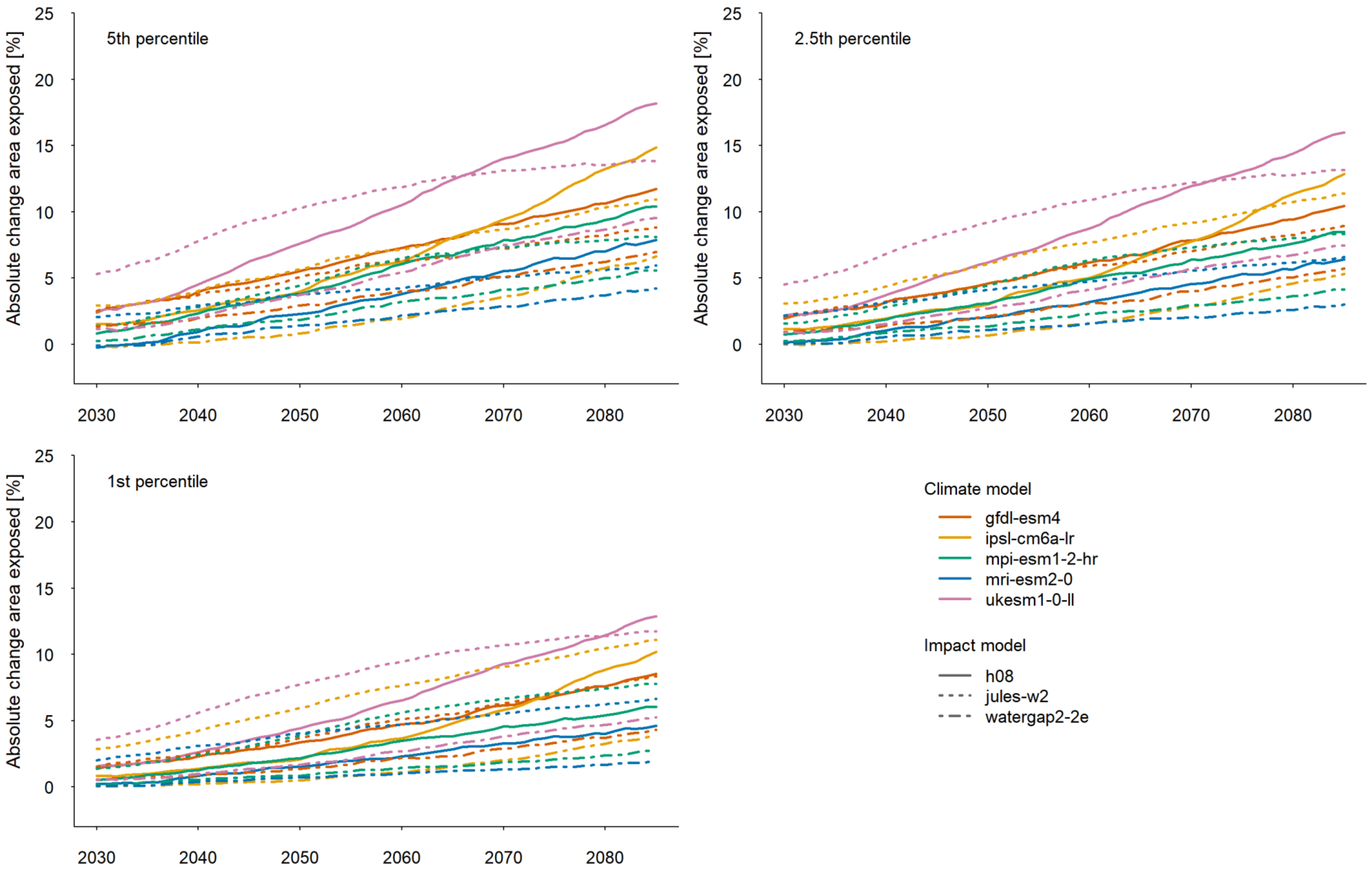

Supplementary Fig. 8c | Sensitivity analysis for threshold used to define droughts for SSP5-8.5.

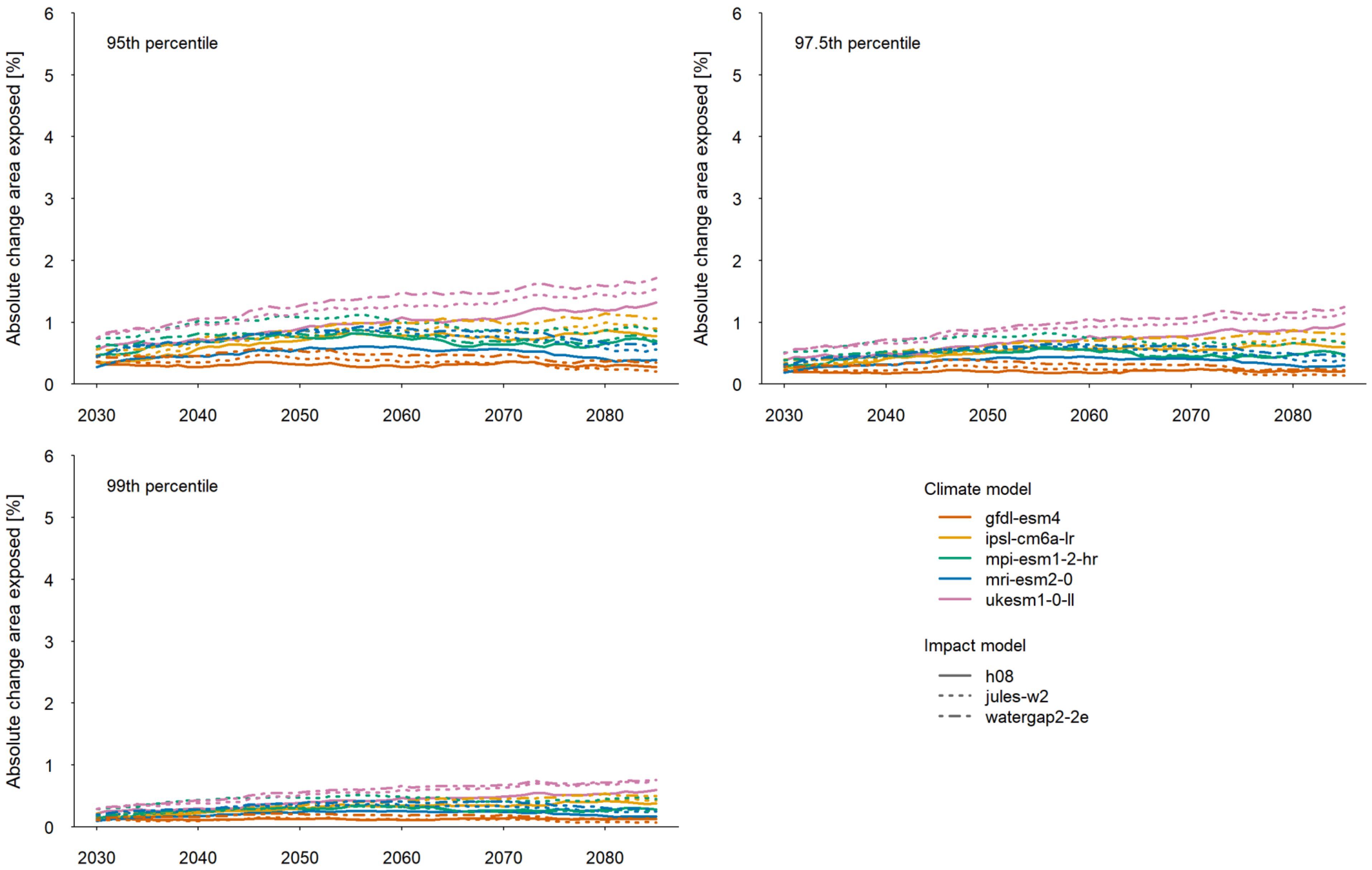

Supplementary Fig. 9a | Sensitivity analysis for threshold used to define floods for SSP1-2.6. In the main analysis, we used a threshold of 40-year return interval which corresponds to the 97.5th percentile to define floods. Plotted is the proportion of area globally exposed to floods using three different thresholds: 95th (20-year return interval), 97.5th (40-year return interval) and 99th (100-year return interval) percentile. The figure shows that the variability due to climate model and impact model is much larger than the variability due to percentile threshold.

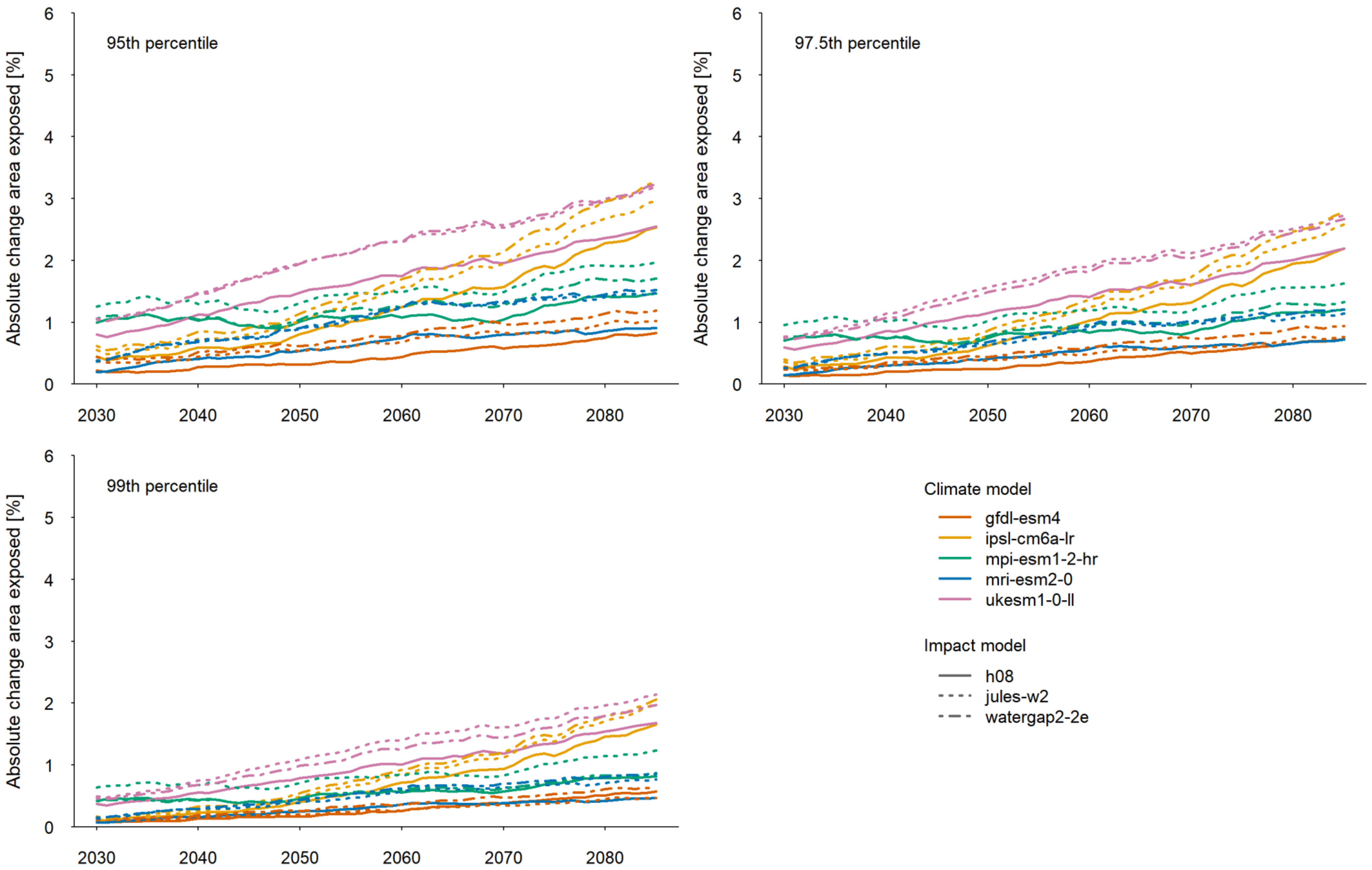

Supplementary Fig. 9b | Sensitivity analysis for threshold used to define floods for SSP3-7.0.

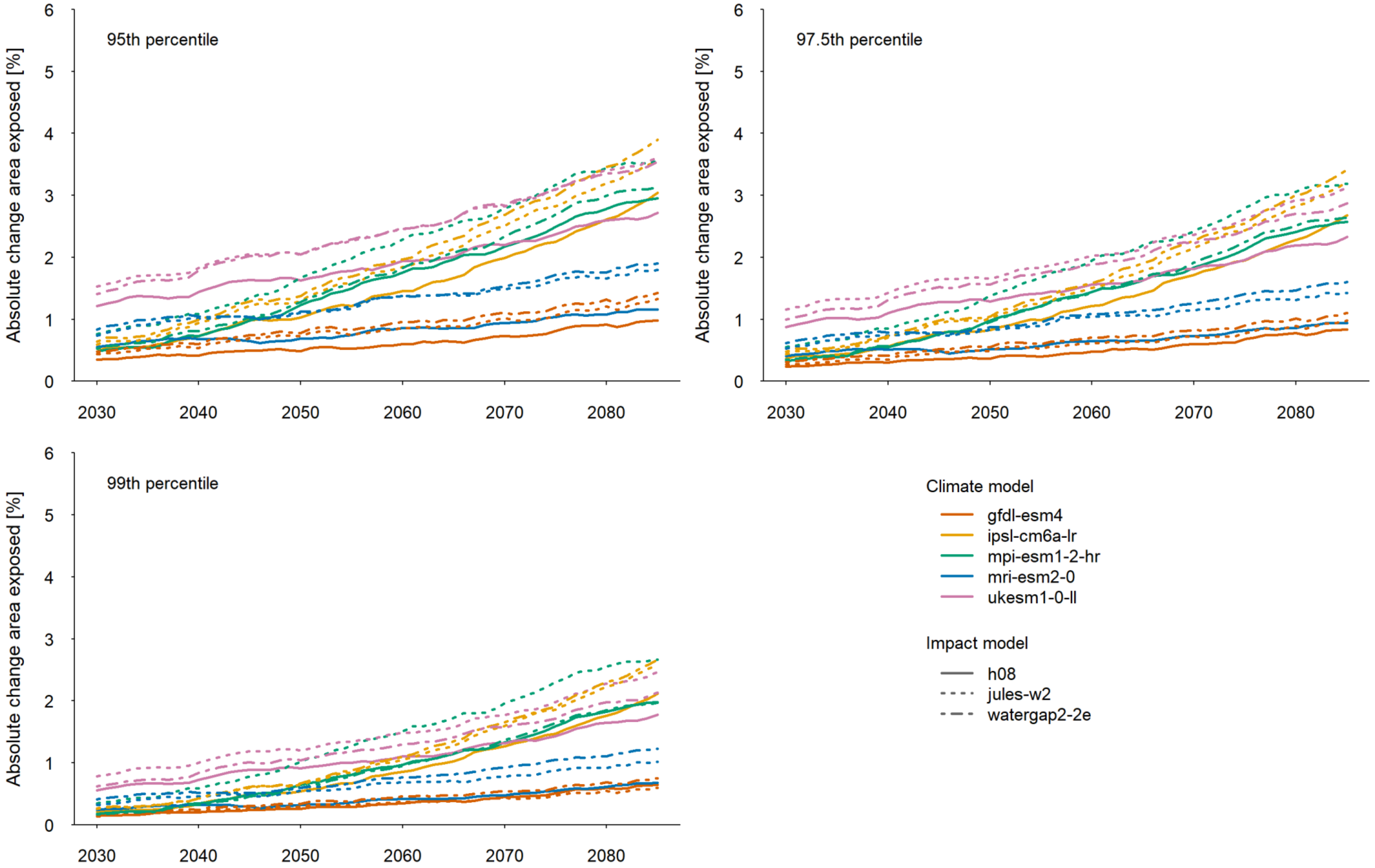

Supplementary Fig. 9c | Sensitivity analysis for threshold used to define floods for SSP5-8.5.

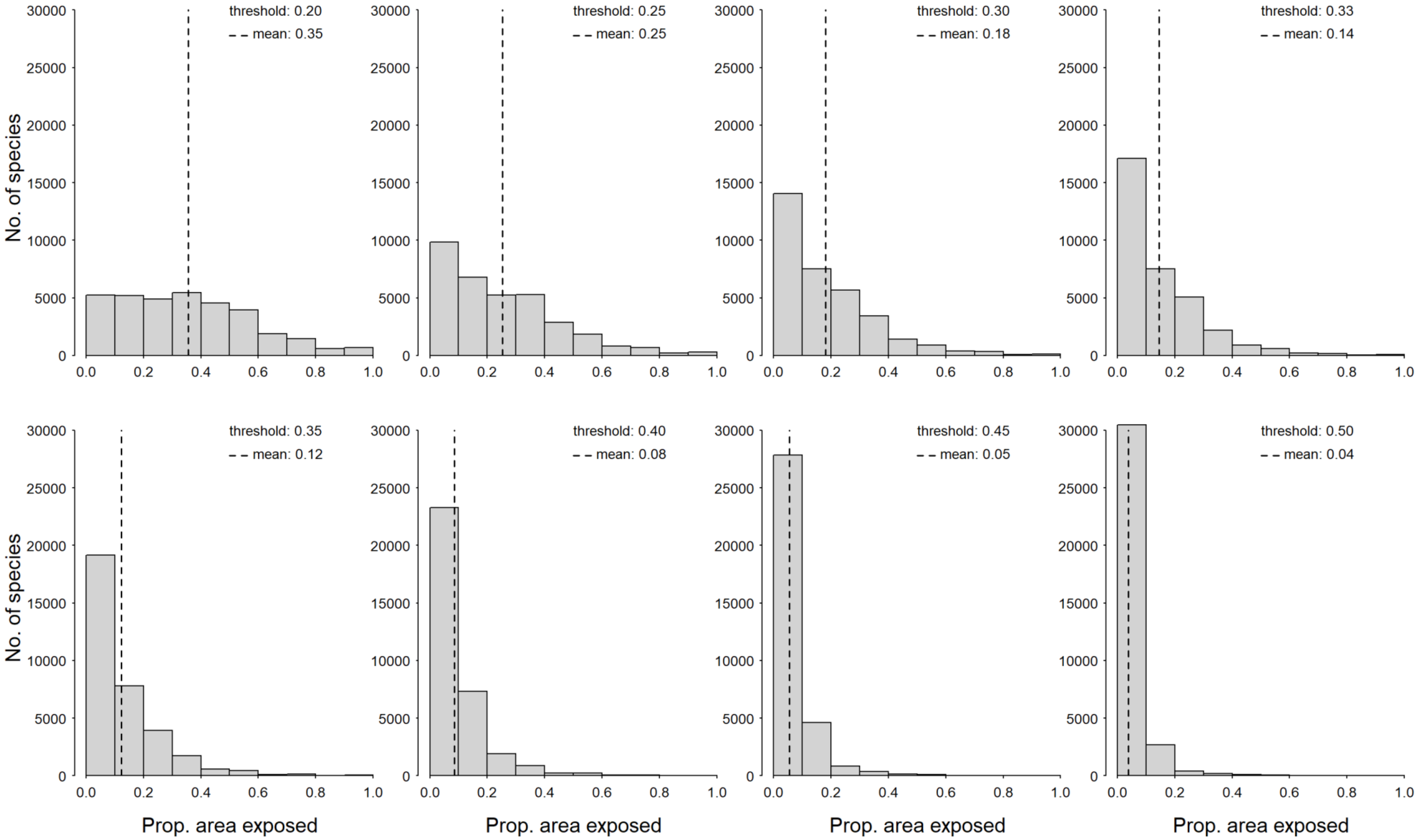

Supplementary Fig. 10a | Sensitivity analysis for threshold used to define multiple events for the year 2050 for SSP3−7.0 for all species. In the main analysis, we used a threshold of 0.33 to identify grid cells with a high frequency for each event type. The figure shows the proportion of geographic range exposed to two or more types of extreme events when different thresholds ranging from 0.2 to 0.5 are applied. The 'mean' is exposure to at least two types of extreme events averaged across all terrestrial vertebrate species (n=33,936 species).

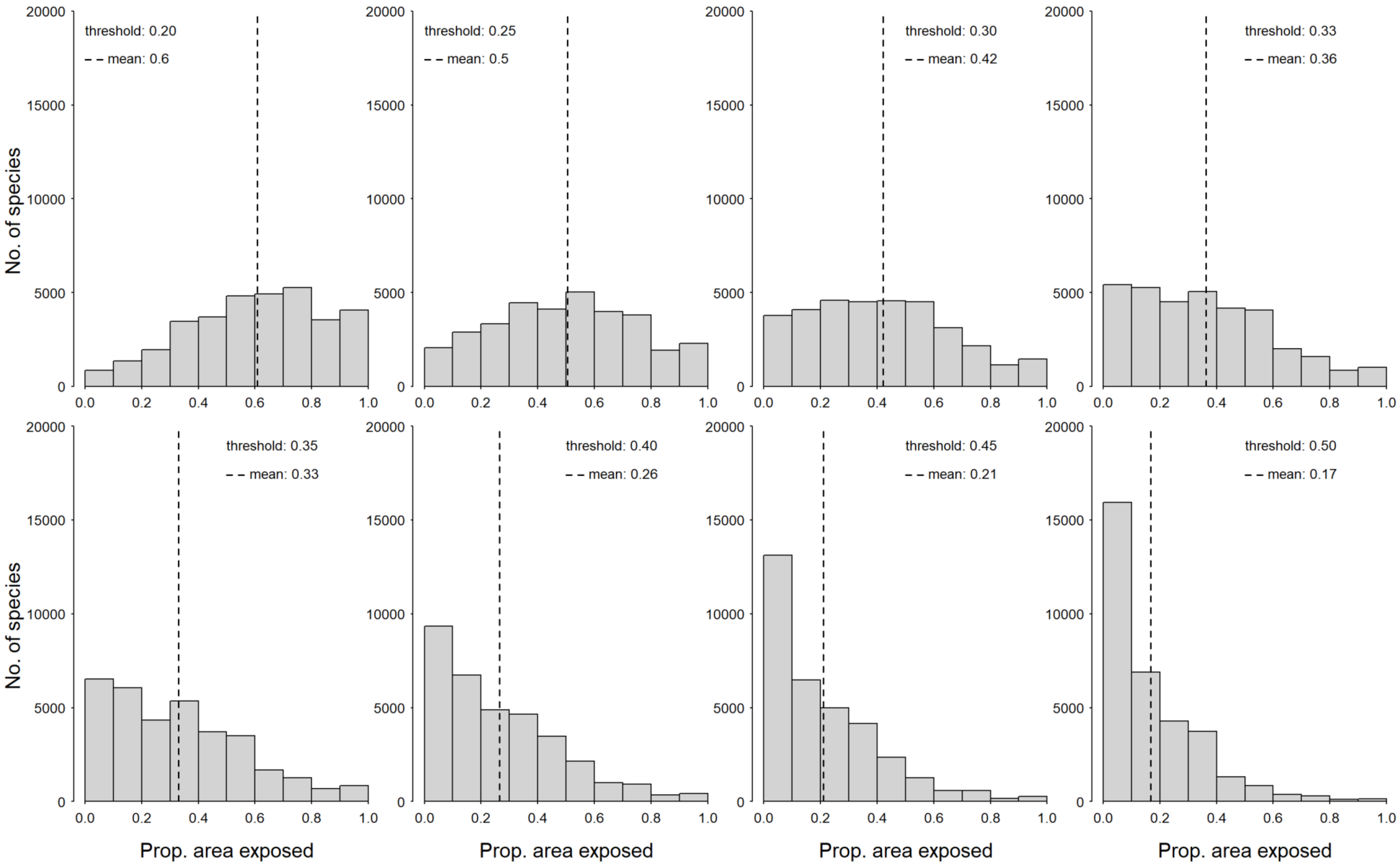

Supplementary Fig. 10b | Sensitivity analysis for threshold used to define multiple events for the year 2085 for SSP3−7.0 for all species.

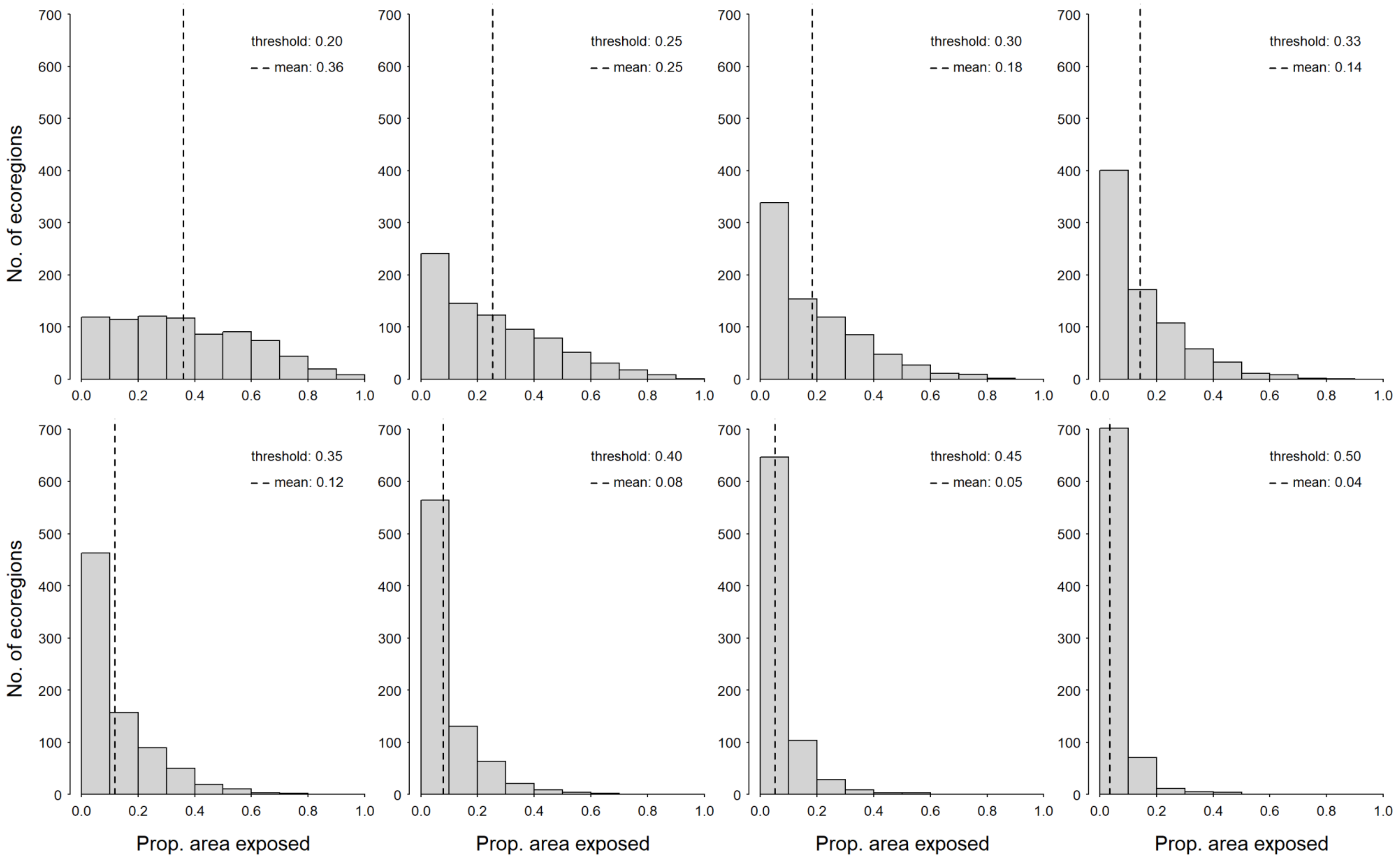

Supplementary Fig. 11a | Sensitivity analysis for threshold used to define multiple events for the year 2050 for SSP3−7.0 for all ecoregions. In the main analysis, we used a threshold of 0.33 to identify grid cells with a high frequency for each event type. The figure shows the proportion of ecoregion exposed to two or more types of extreme events when different thresholds ranging from 0.2 to 0.5 are applied. The 'mean' is exposure to at least two types of extreme events averaged across all ecoregions (n=794).

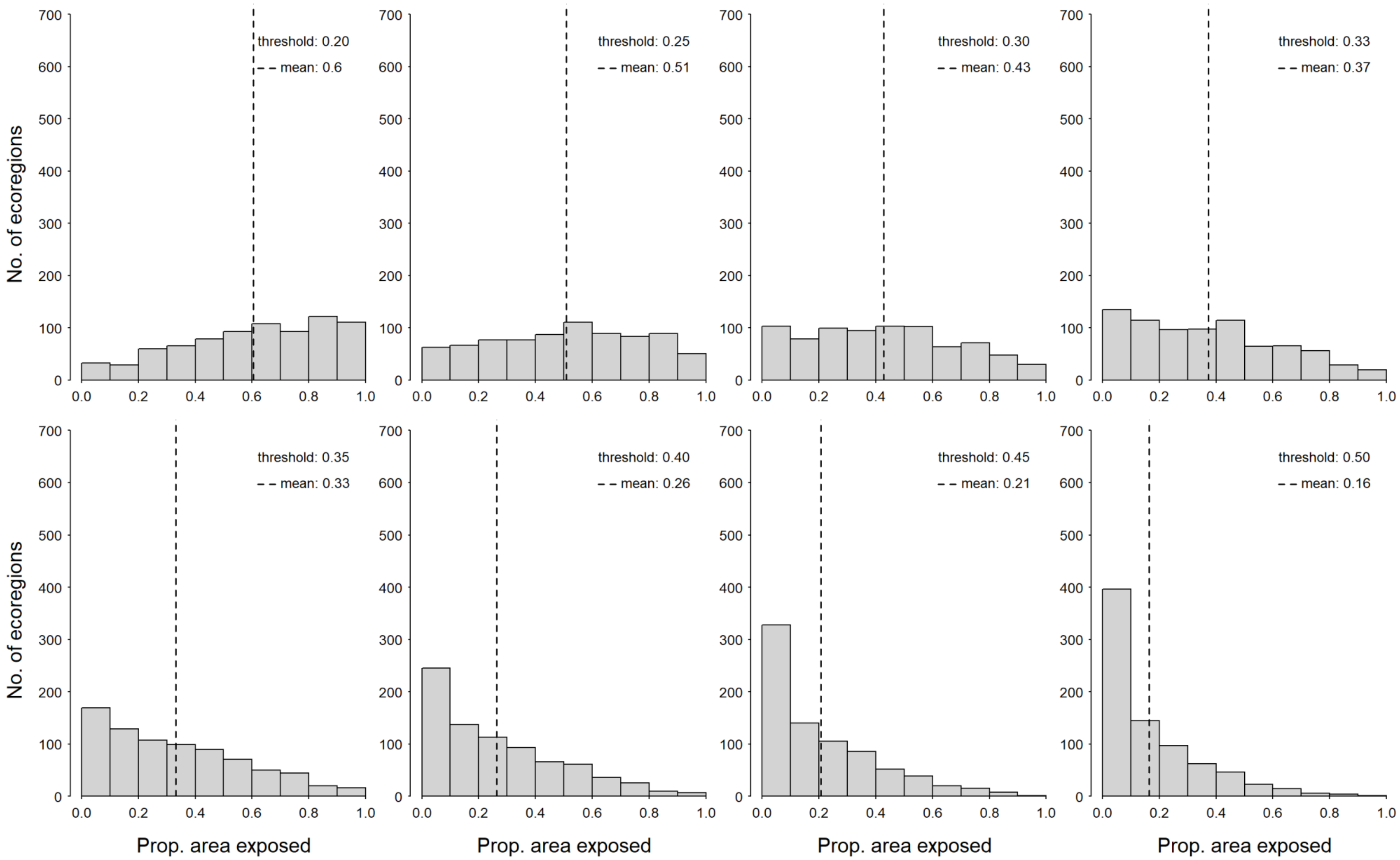

Supplementary Fig. 11b | Sensitivity analysis for threshold used to define multiple events for the year 2085 for SSP3−7.0 for all ecoregions.

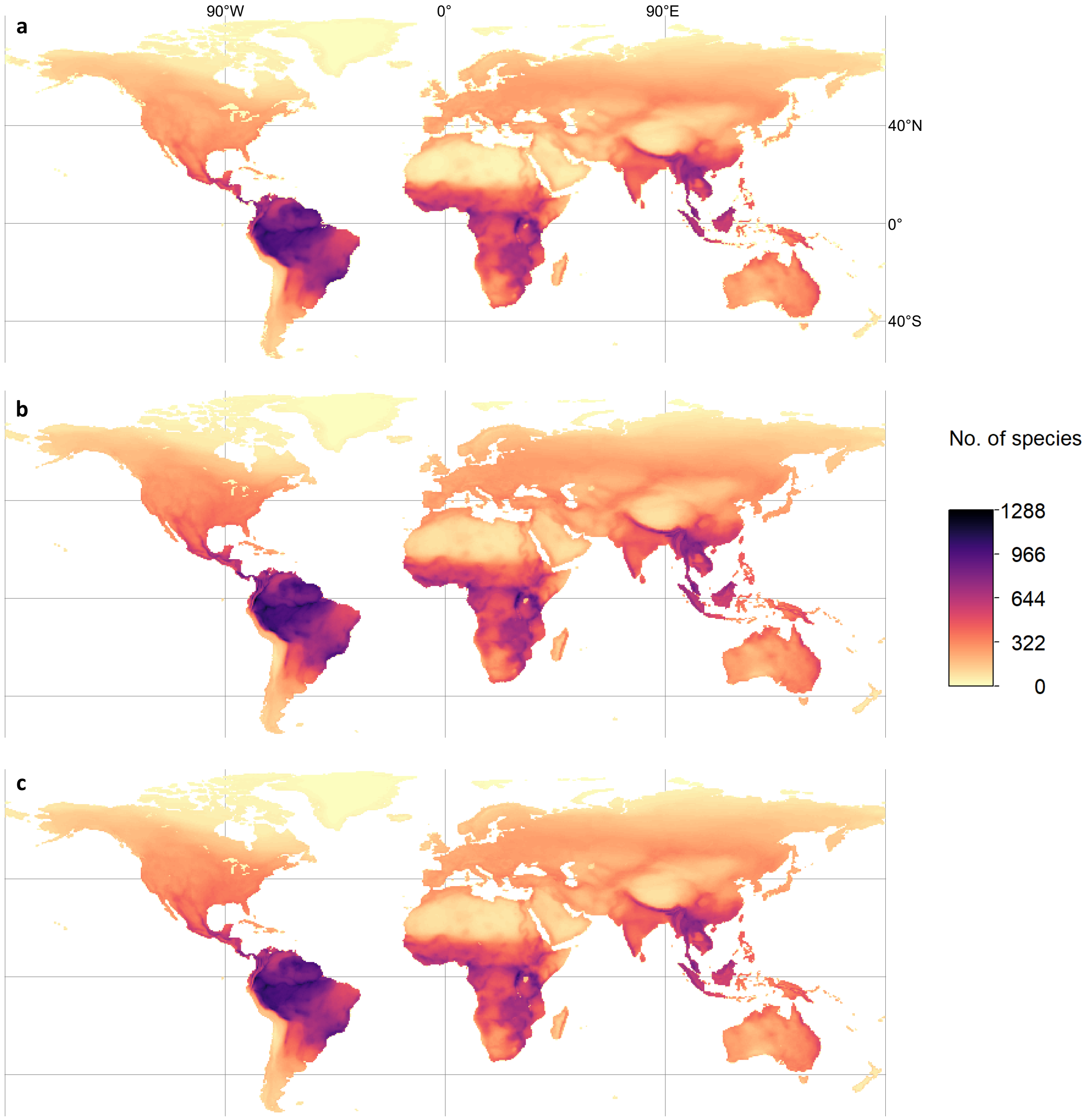

Supplementary Fig. 12 | Species richness patterns derived from different data sources. Species richness derived from **(a)** IUCN Red List aggregated data (used in Fig. 1, Extended Data Fig. 1, Supplementary Fig. 1 and 2), **(b)** individual species range maps with species considered present when their range overlaps a grid cell by ≥10%, and **(c)** individual species range maps with ≥20% overlap threshold. All three approaches yield consistent spatial patterns of species richness.

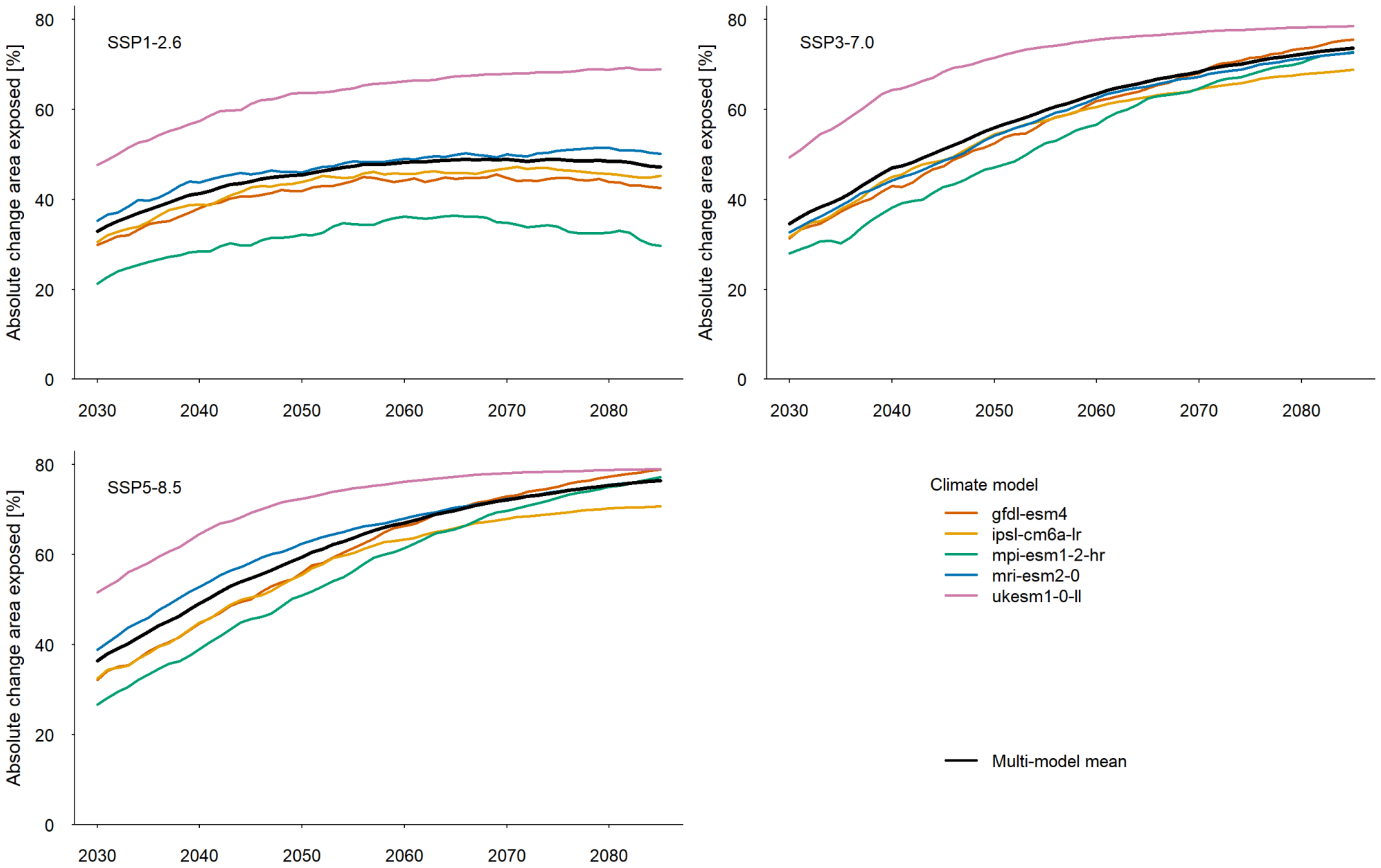

Supplementary Fig. 13a | Exposure of amphibians to projected change in heatwave occurrences. Change in proportion of area exposed relative to year 2000 averaged across all species (n=7,605) for the three scenarios: SSP1−2.6, SSP3−7.0 and SSP5−8.5. Coloured lines are individual climate model – impact model combinations. Black solid line is the multi-model mean.

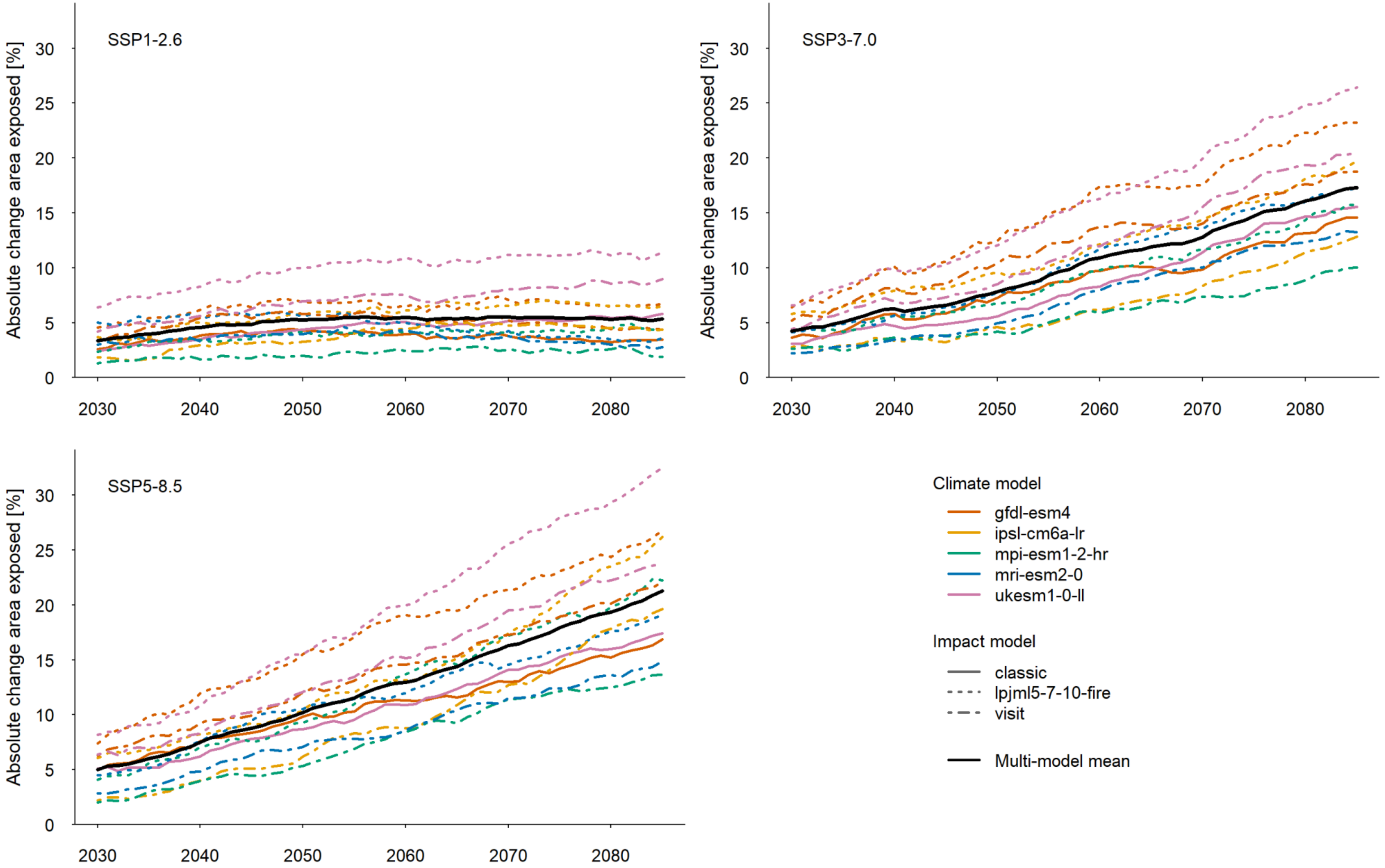

Supplementary Fig. 13b | Exposure of amphibians to projected change in wildfire occurrences.

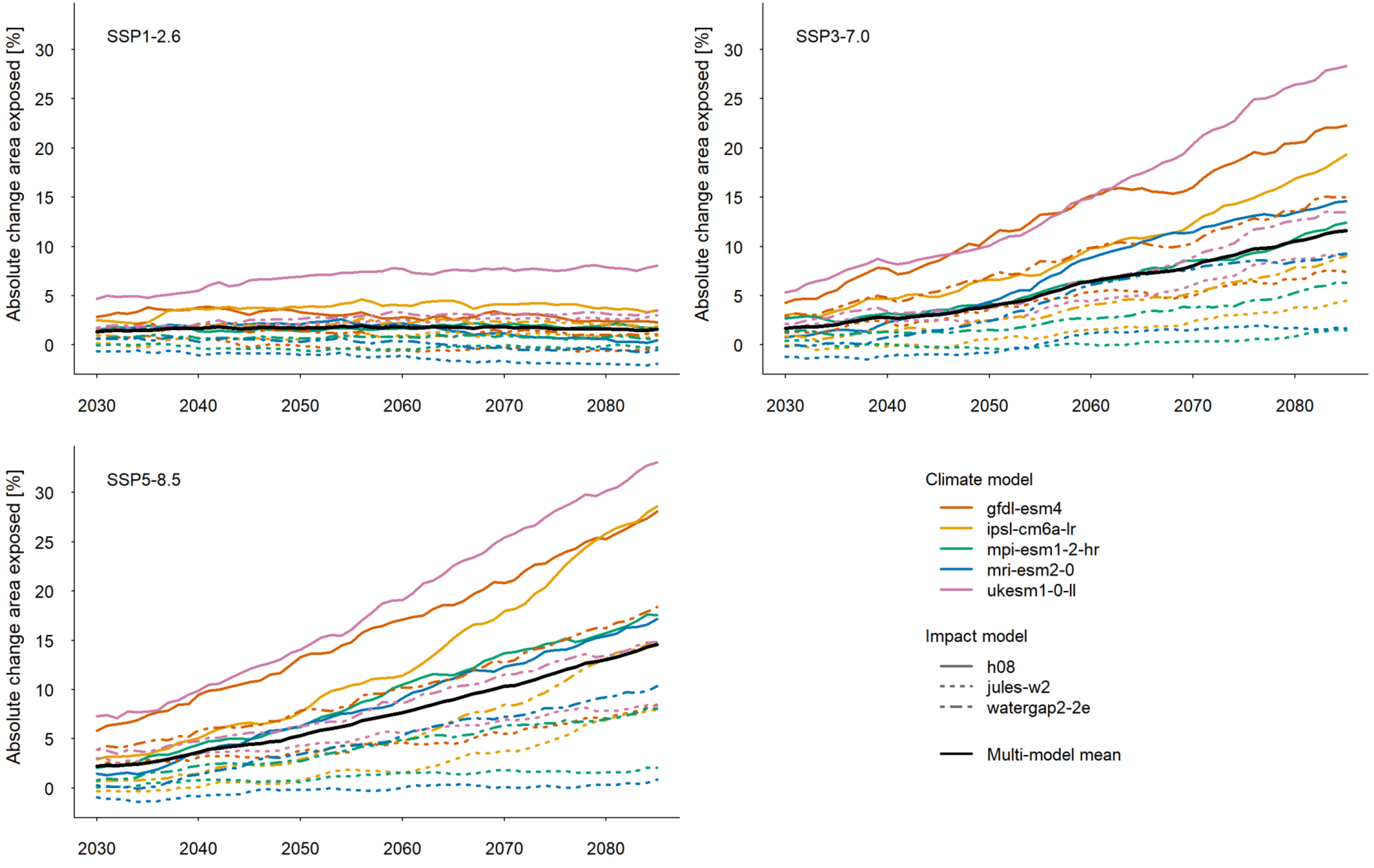

Supplementary Fig. 13c | Exposure of amphibians to projected change in drought occurrences.

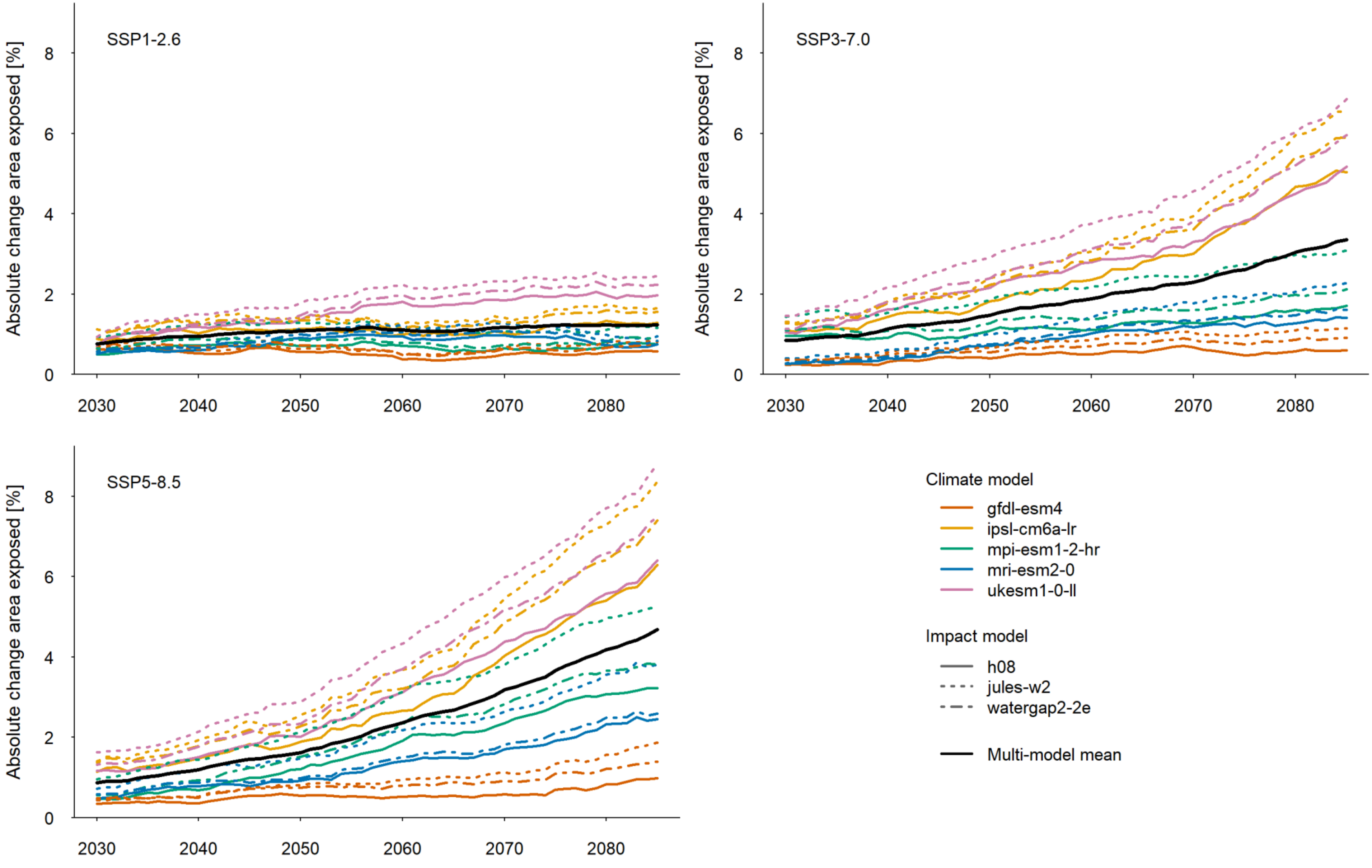

Supplementary Fig. 13d | Exposure of amphibians to projected change in river flood occurrences.

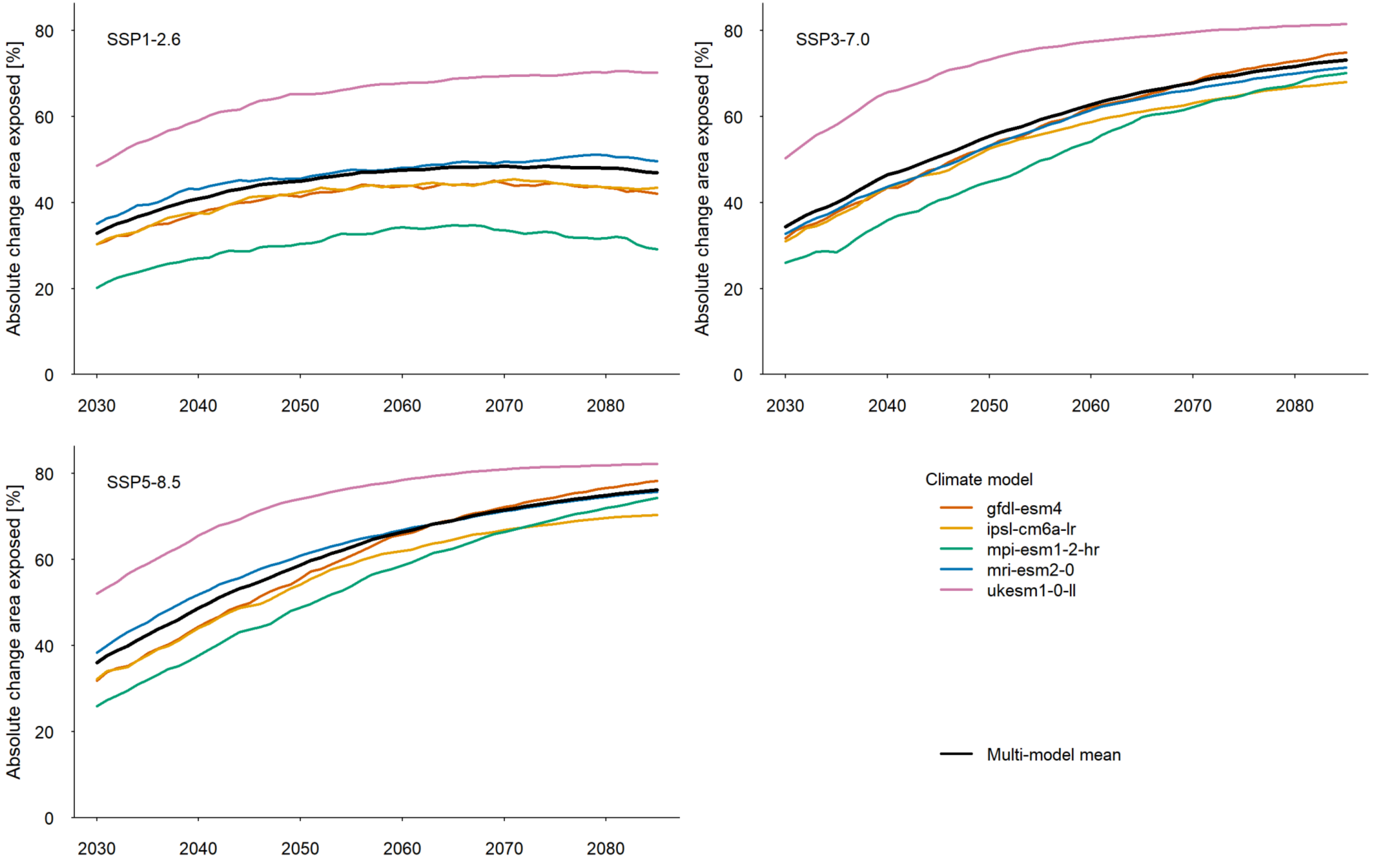

Supplementary Fig. 14a | Exposure of birds to projected change in heatwave occurrences. Change in proportion of area exposed relative to year 2000 averaged across all species (n=10,562) for the three scenarios: SSP1−2.6, SSP3−7.0 and SSP5−8.5. Coloured lines are individual climate model – impact model combinations. Black solid line is the multi-model mean.

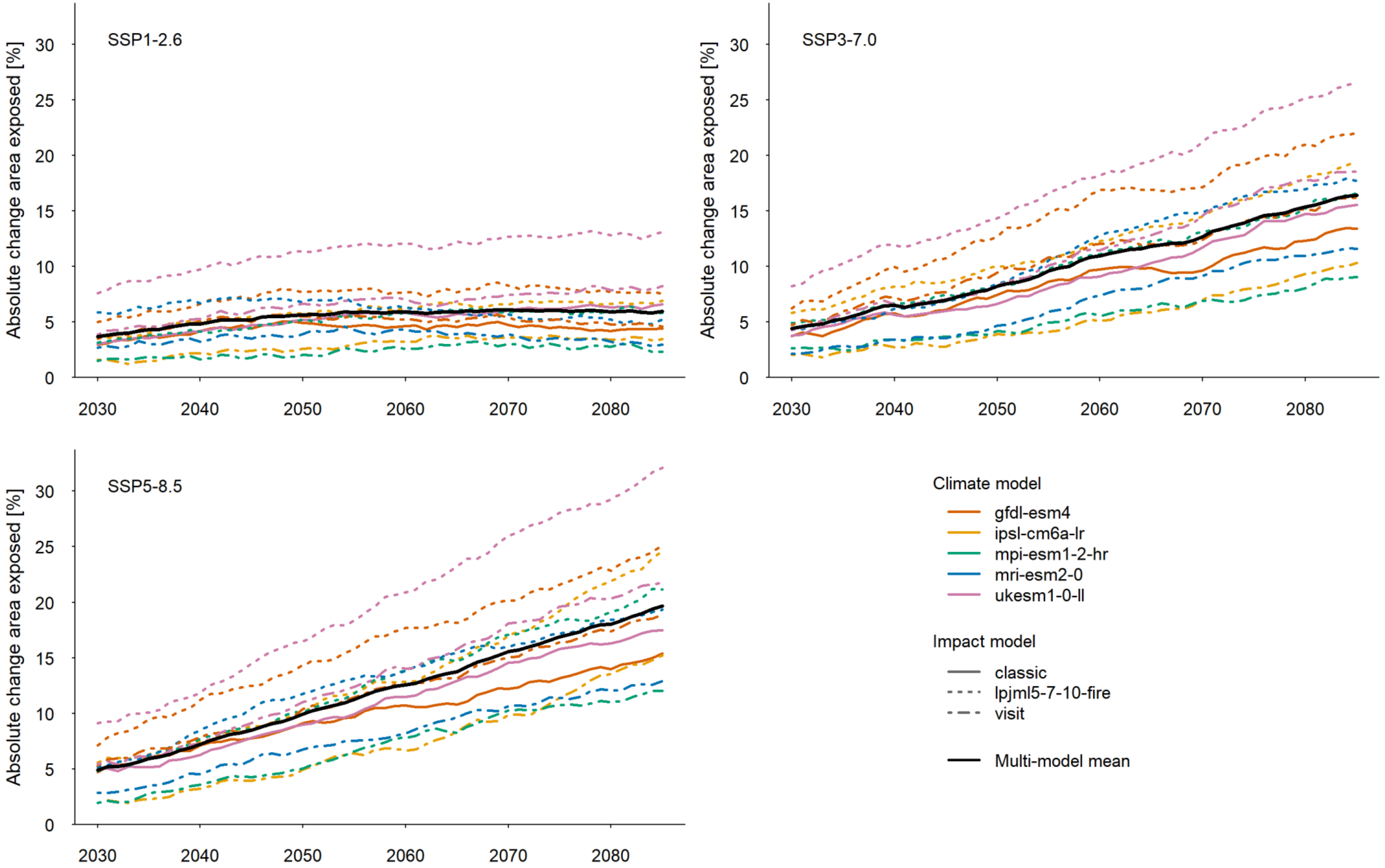

Supplementary Fig. 14b | Exposure of birds to projected change in wildfire occurrences.

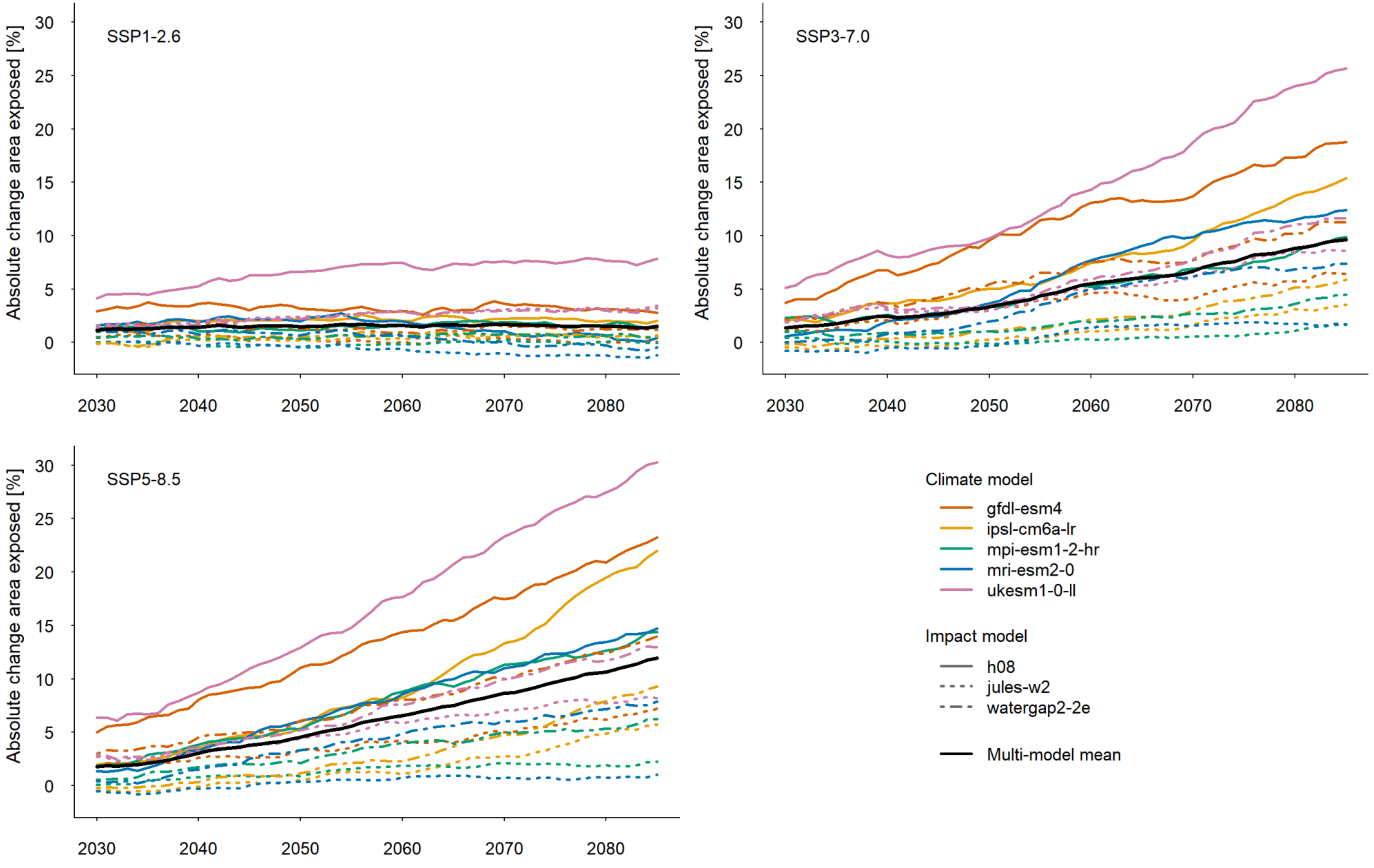

Supplementary Fig. 14c | Exposure of birds to projected change in drought occurrences.

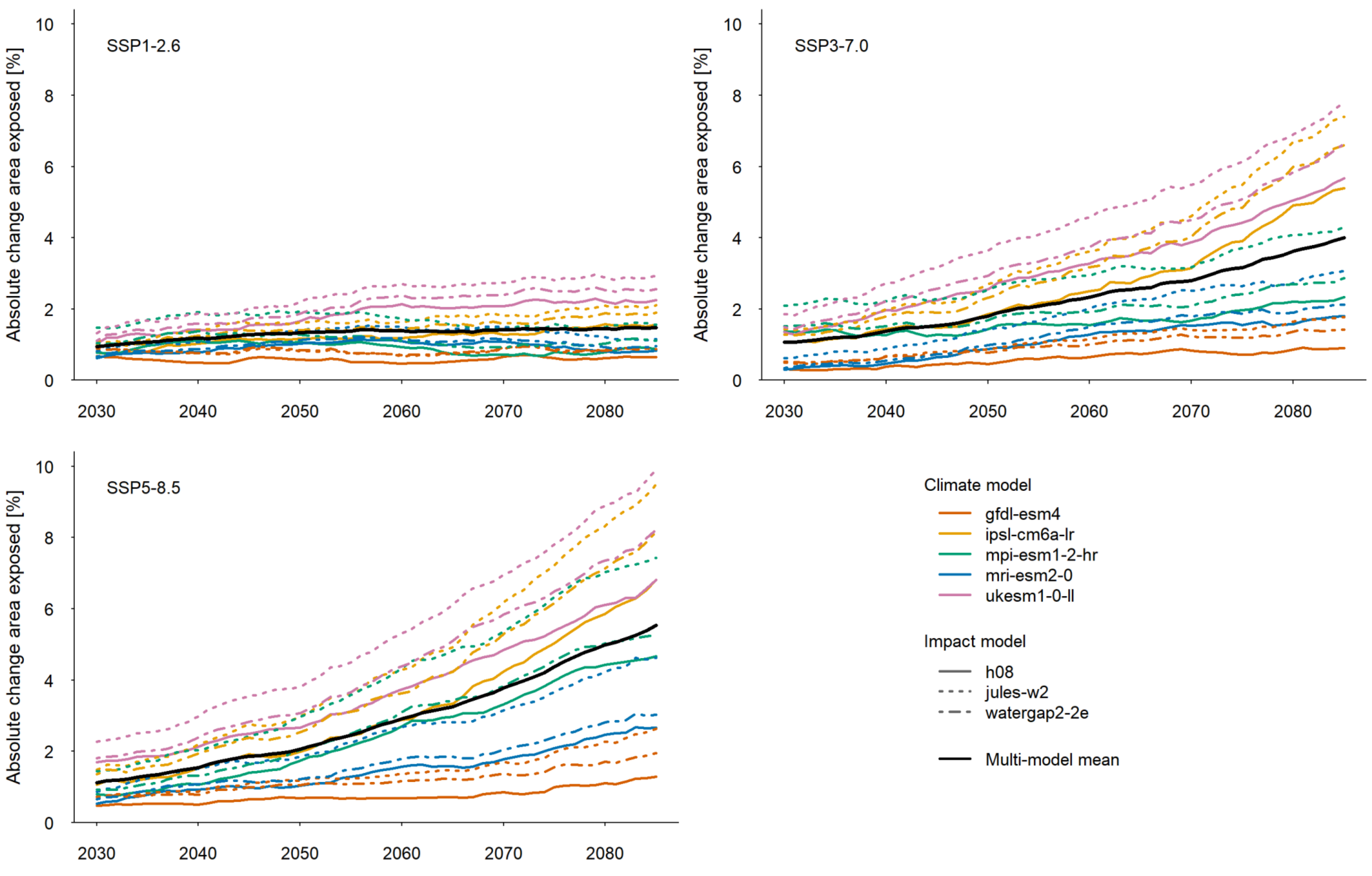

Supplementary Fig. 14d | Exposure of birds to projected change in river flood occurrences.

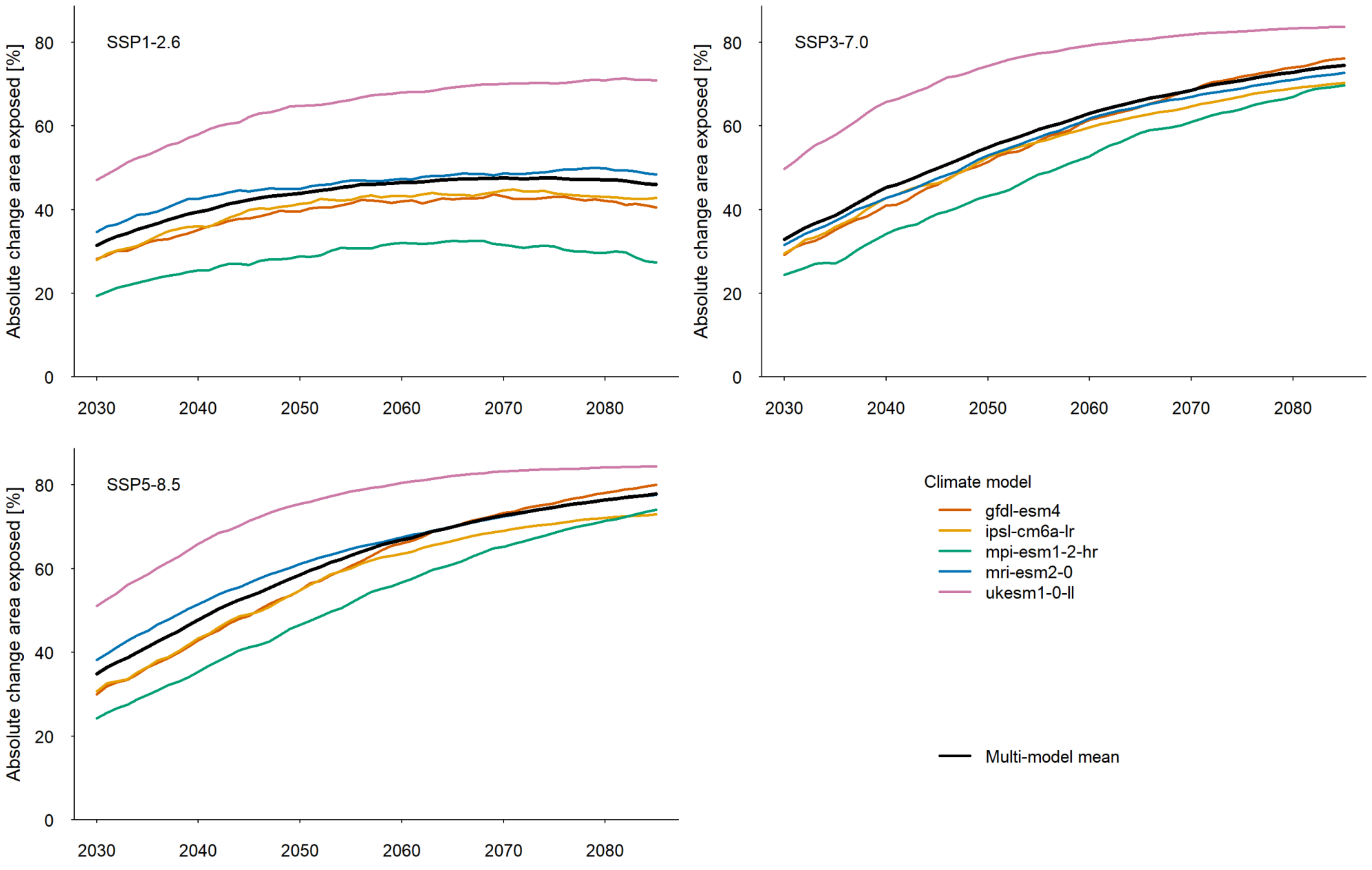

Supplementary Fig. 15a | Exposure of mammals to projected change in heatwave occurrences. Change in proportion of area exposed relative to year 2000 averaged across all species (n=5,476) for the three scenarios: SSP1−2.6, SSP3−7.0 and SSP5−8.5. Coloured lines are individual climate model – impact model combinations. Black solid line is the multi-model mean.

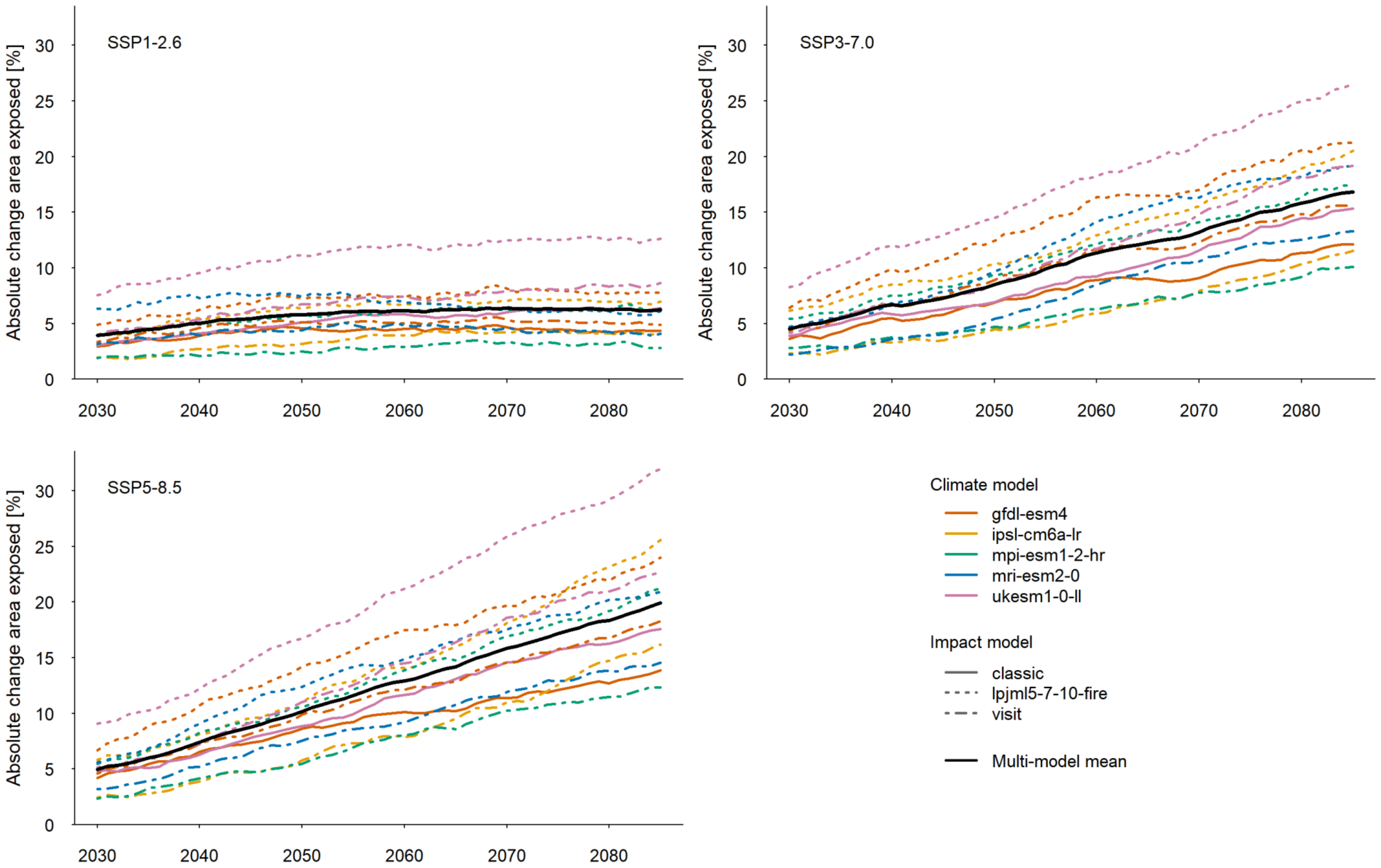

Supplementary Fig. 15b | Exposure of mammals to projected change in wildfire occurrences.

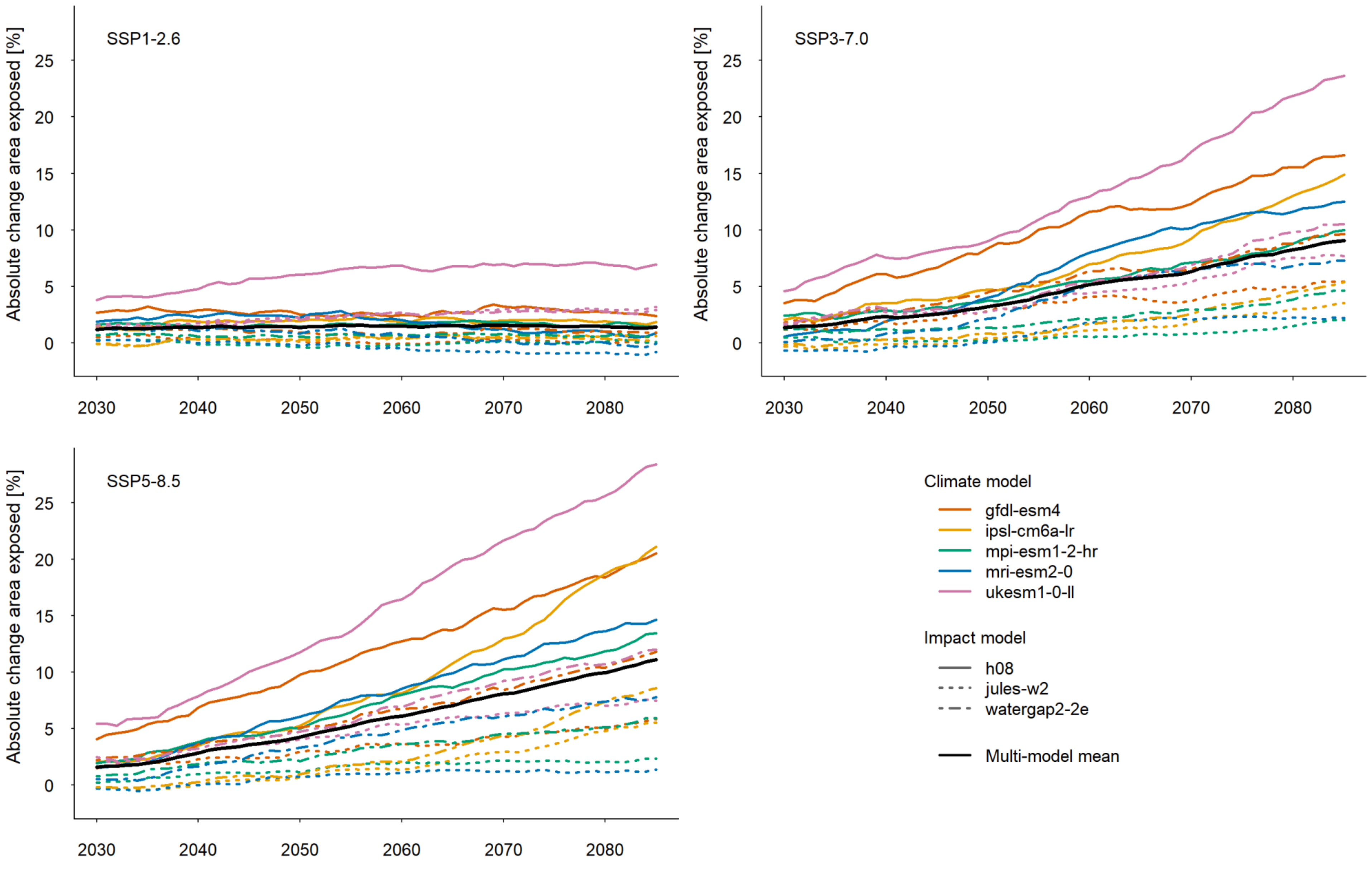

Supplementary Fig. 15c | Exposure of mammals to projected change in drought occurrences.

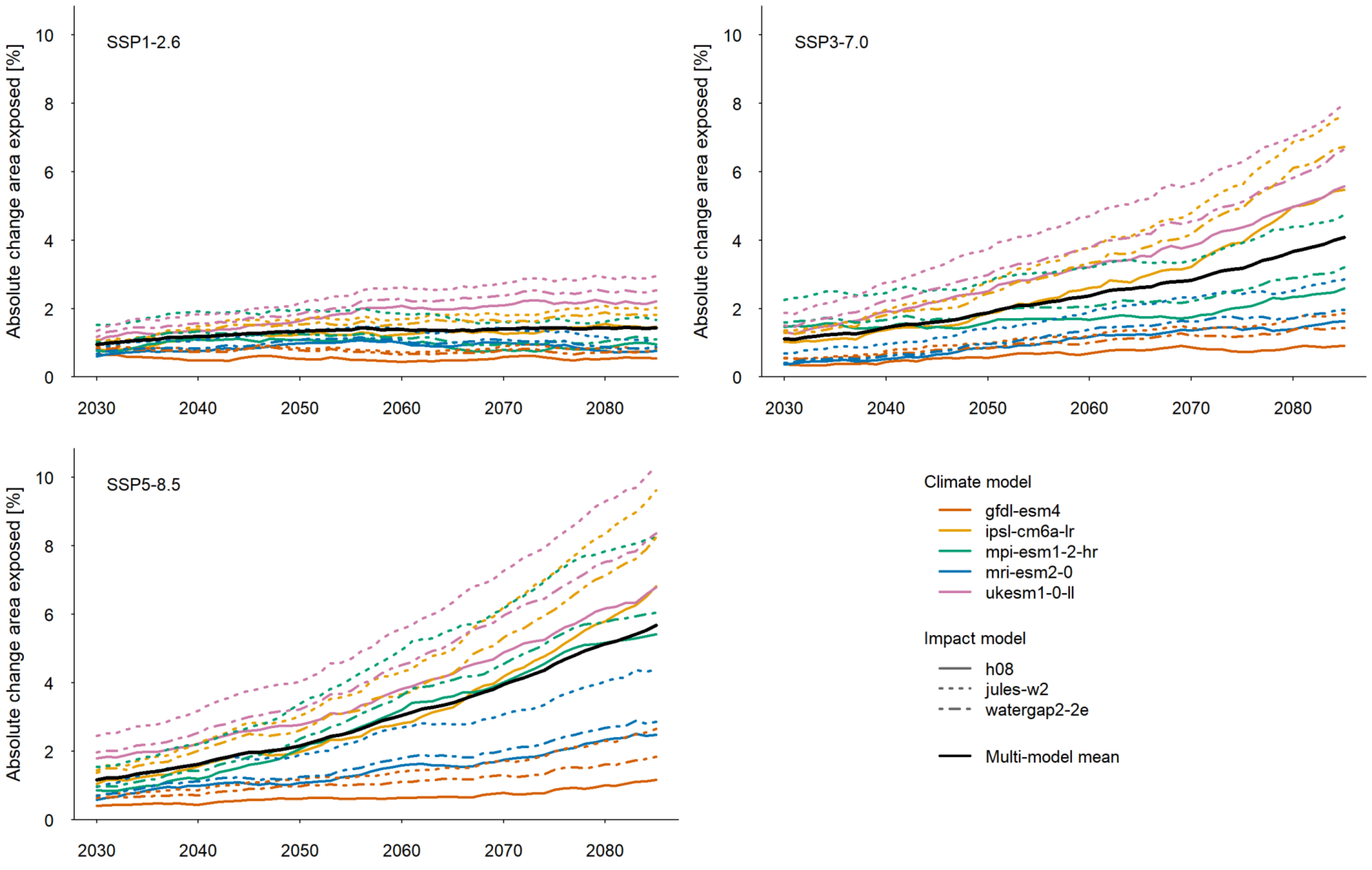

Supplementary Fig. 15d | Exposure of mammals to projected change in river flood occurrences.

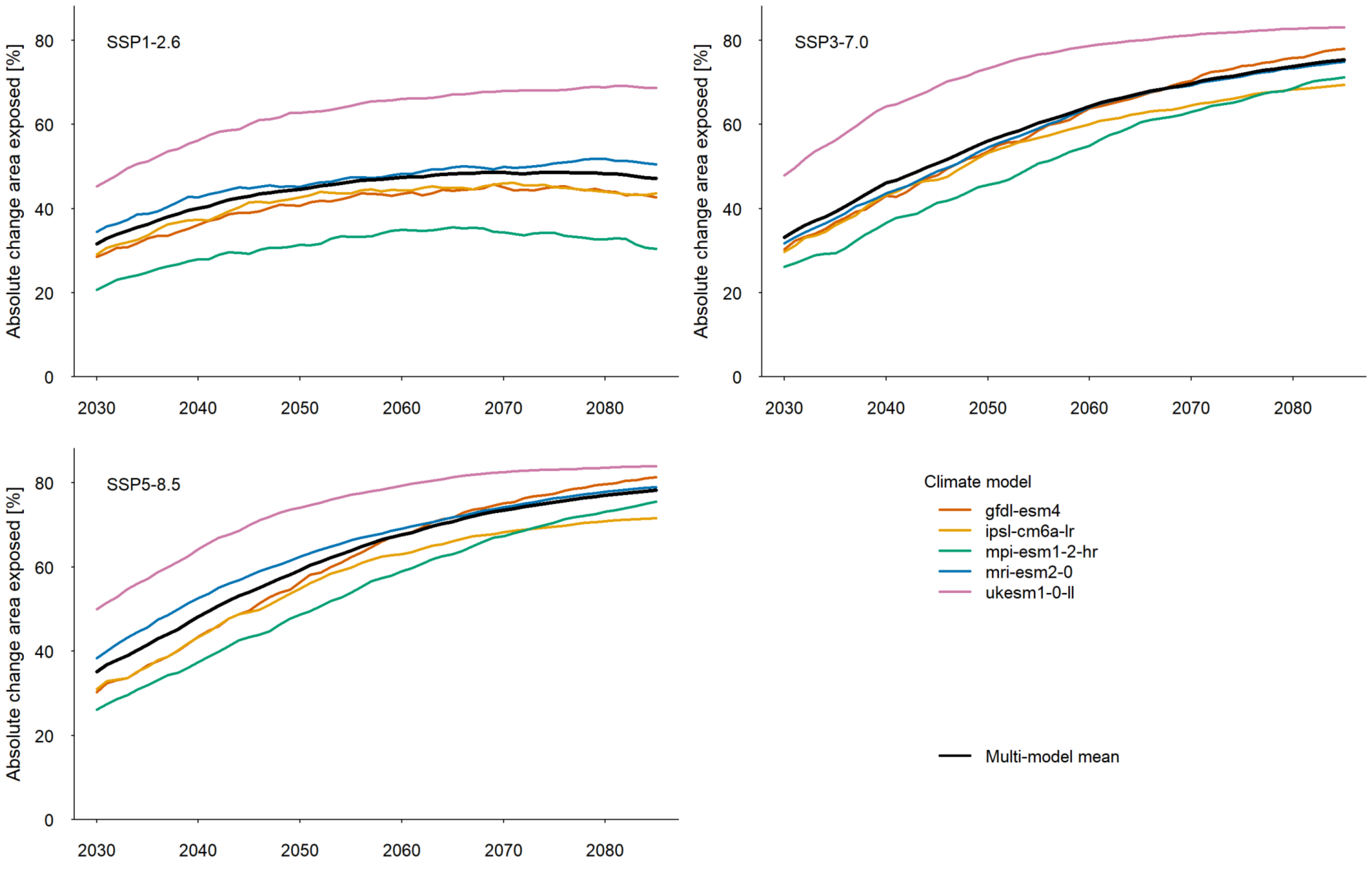

Supplementary Fig. 16a | Exposure of reptiles to projected change in heatwave occurrences. Change in proportion of area exposed relative to year 2000 averaged across all species (n=10,293) for the three scenarios: SSP1−2.6, SSP3−7.0 and SSP5−8.5. Coloured lines are individual climate model – impact model combinations. Black solid line is the multi-model mean.

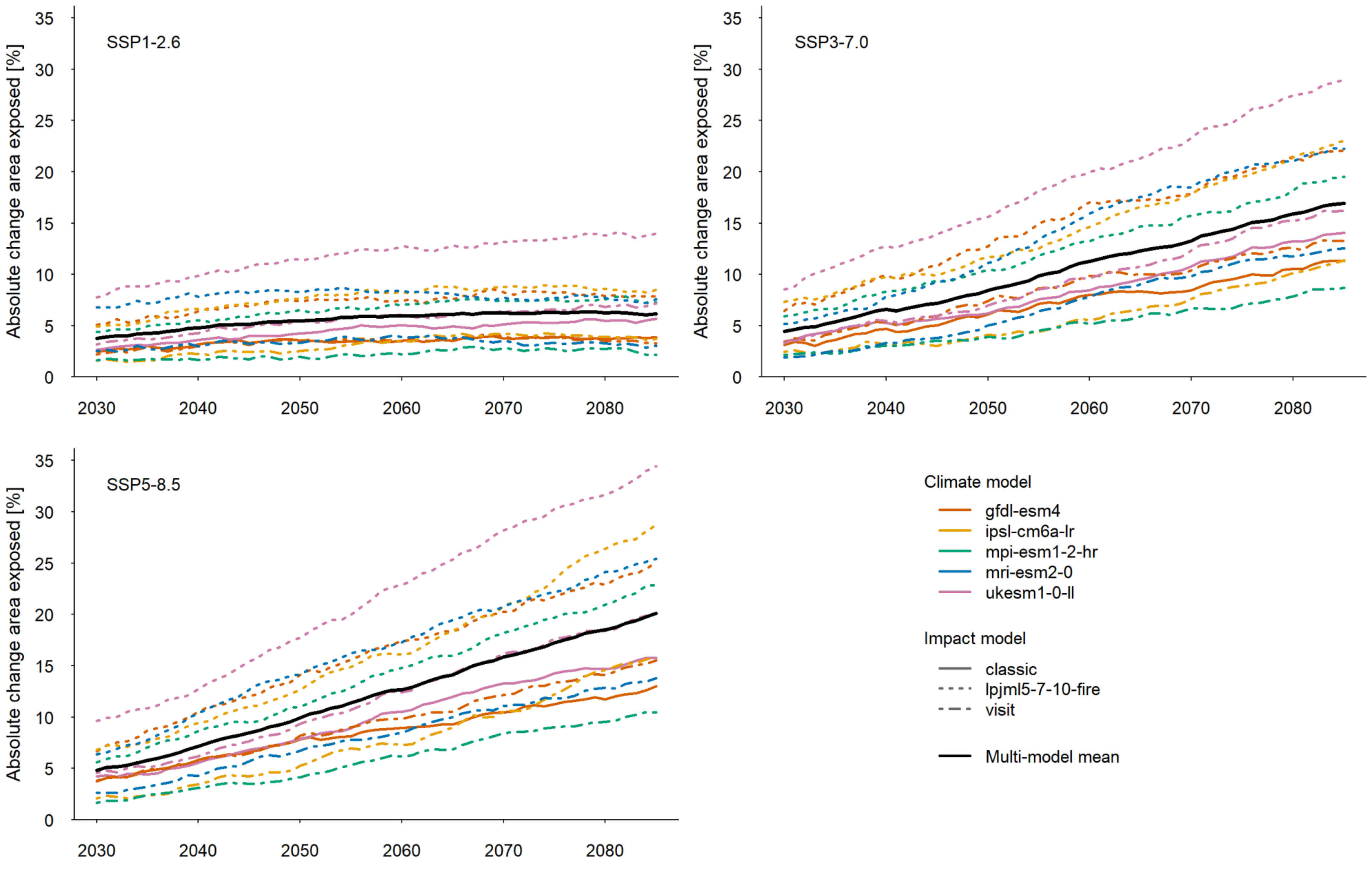

Supplementary Fig. 16b | Exposure of reptiles to projected change in wildfire occurrences.

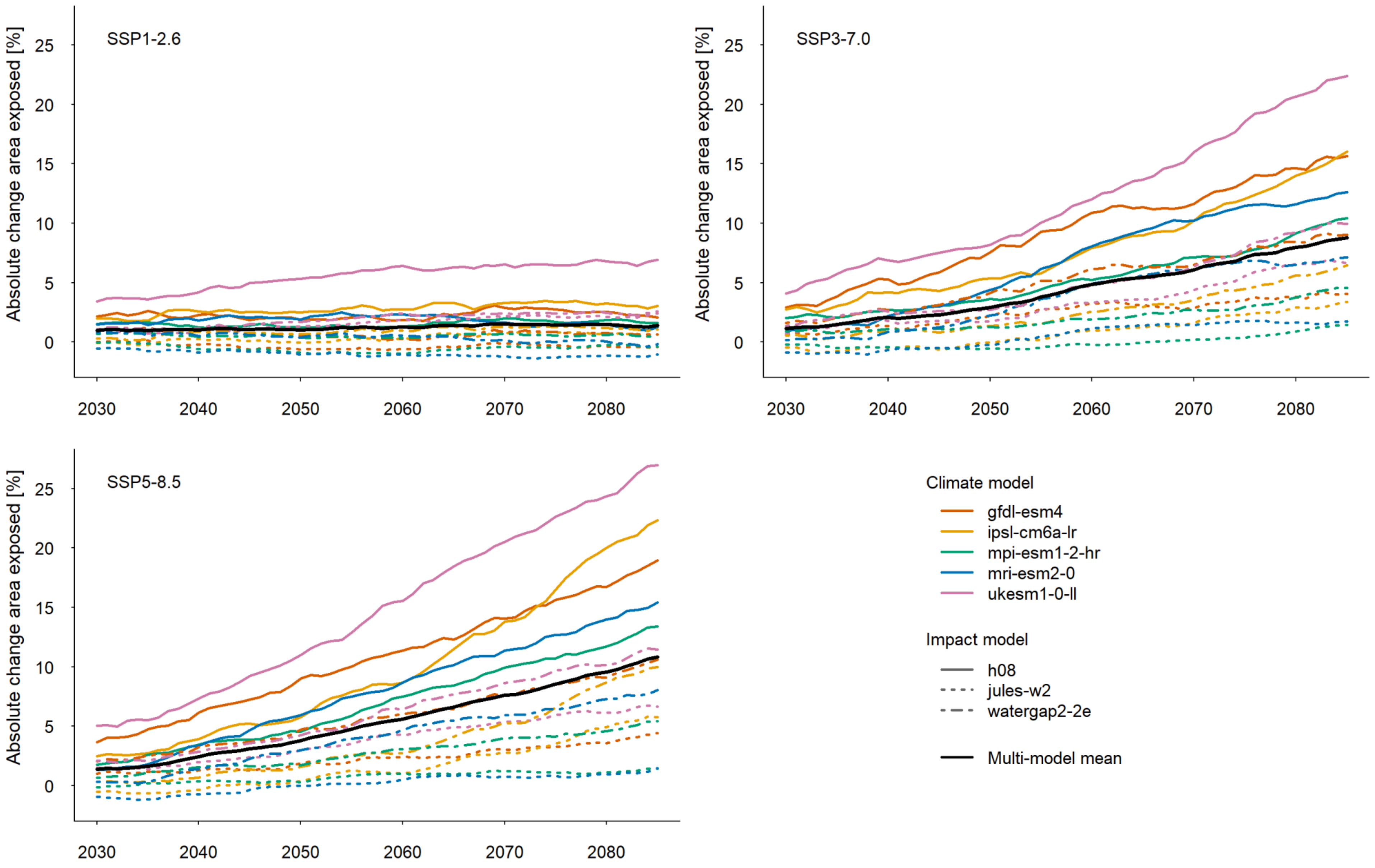

Supplementary Fig. 16c | Exposure of reptiles to projected change in drought occurrences.

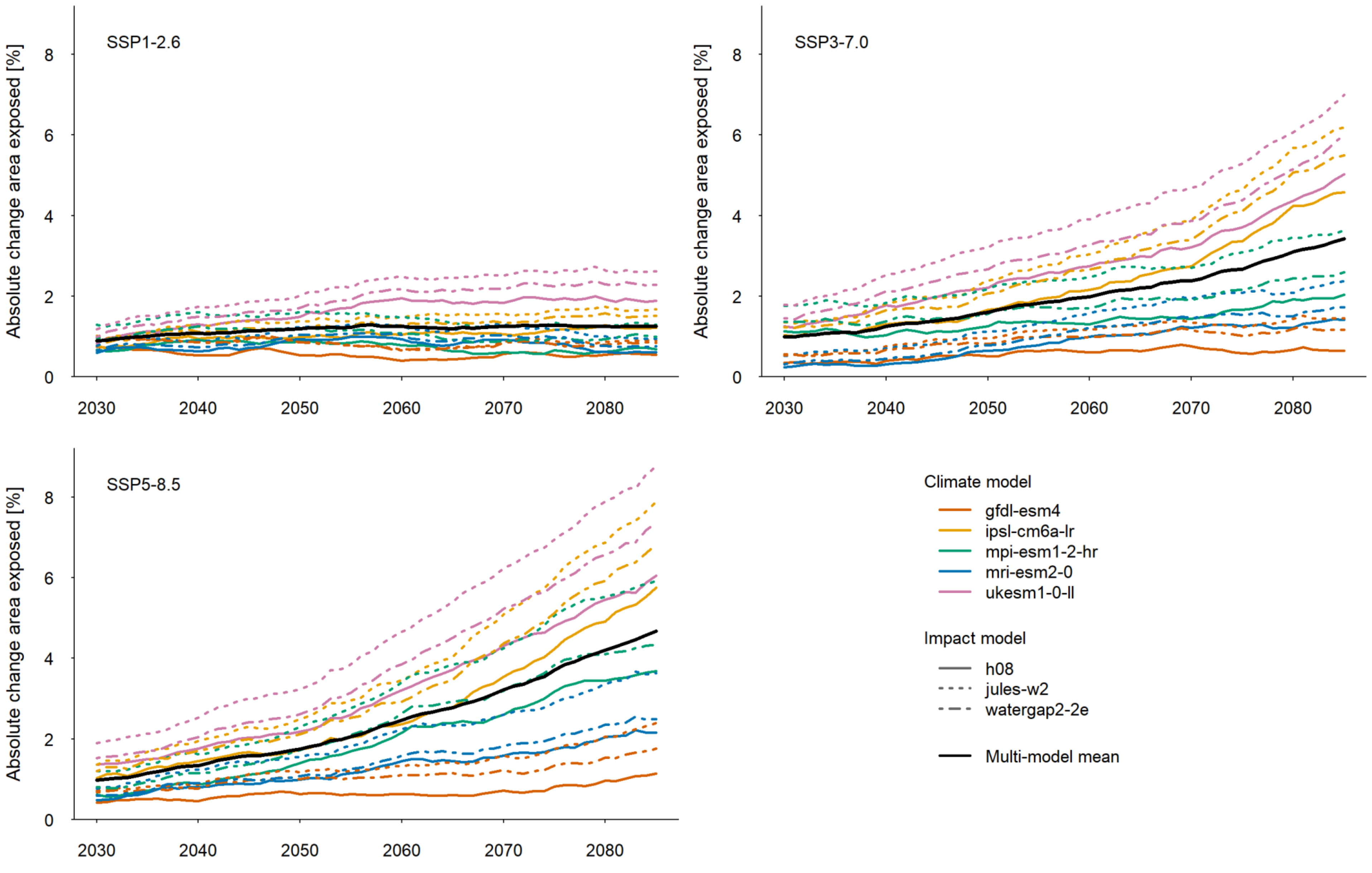

Supplementary Fig. 16d | Exposure of reptiles to projected change in river flood occurrences.

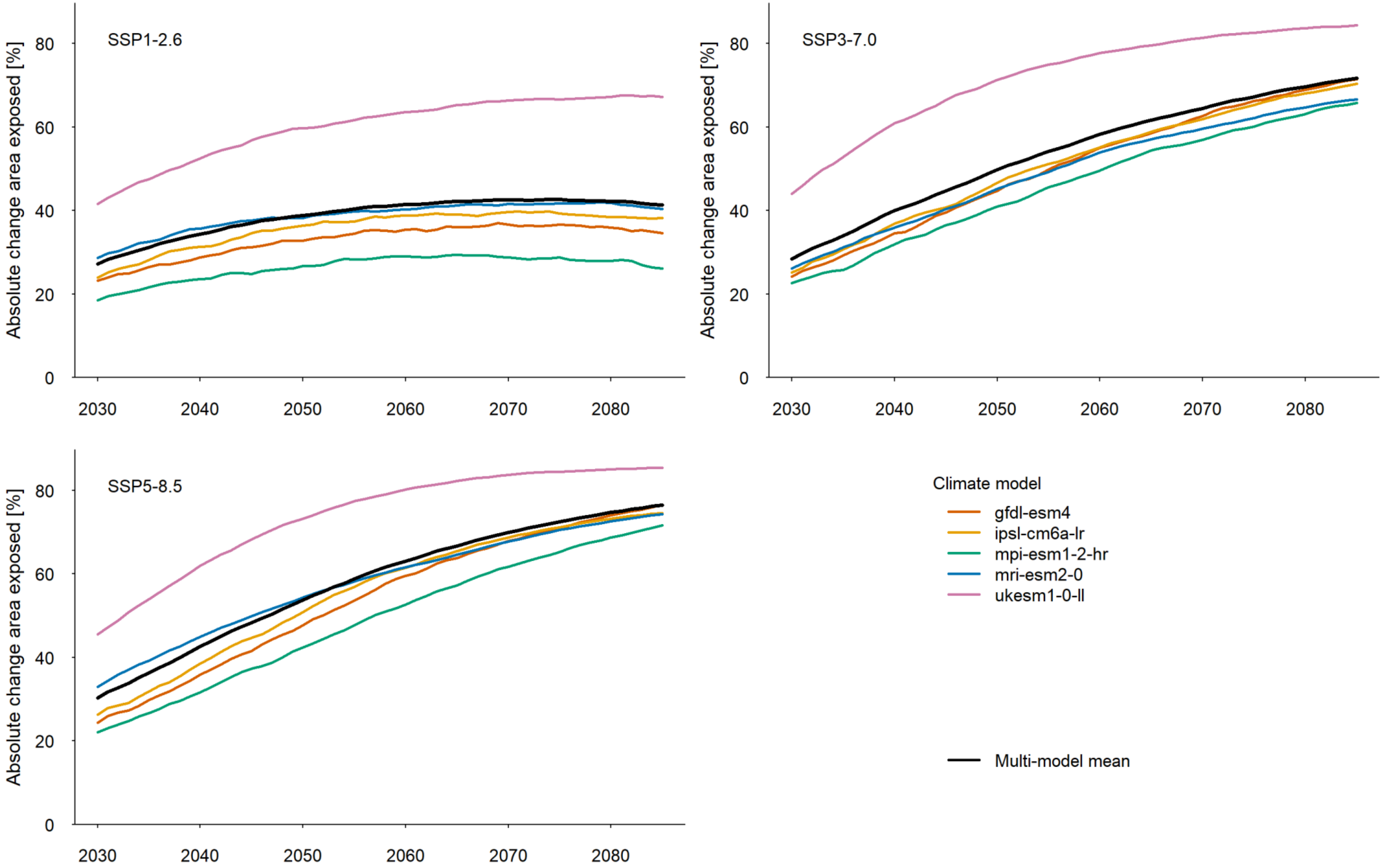

Supplementary Fig. 17a | Exposure of ecoregions to projected change in heatwave occurrences. Change in proportion of area exposed relative to year 2000 averaged across all ecoregions (n=794) for the three scenarios: SSP1−2.6, SSP3−7.0 and SSP5−8.5. Coloured lines are individual climate model – impact model combinations. Black solid line is the multi-model mean.

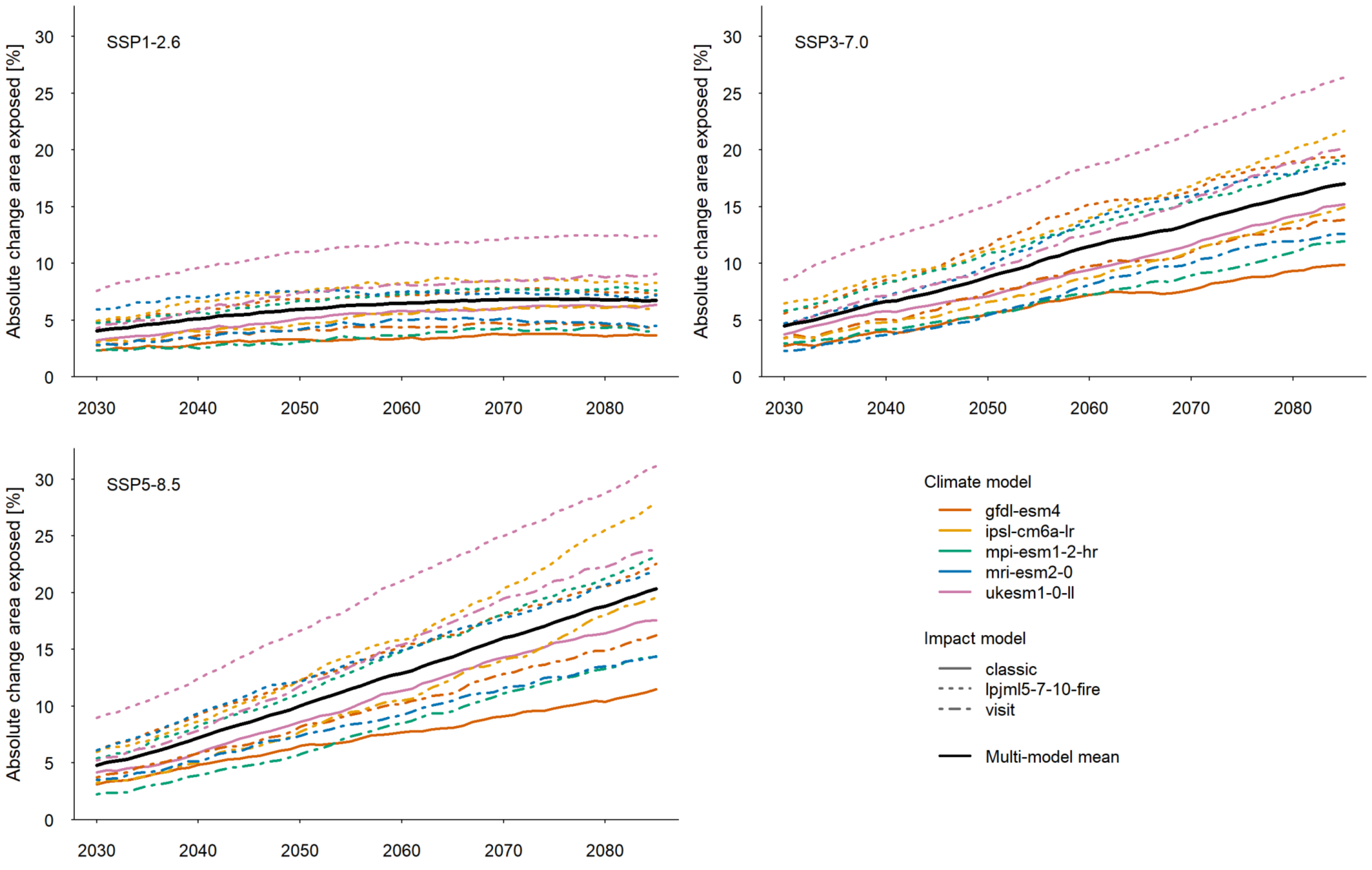

Supplementary Fig. 17b | Exposure of ecoregions to projected change in wildfire occurrences.

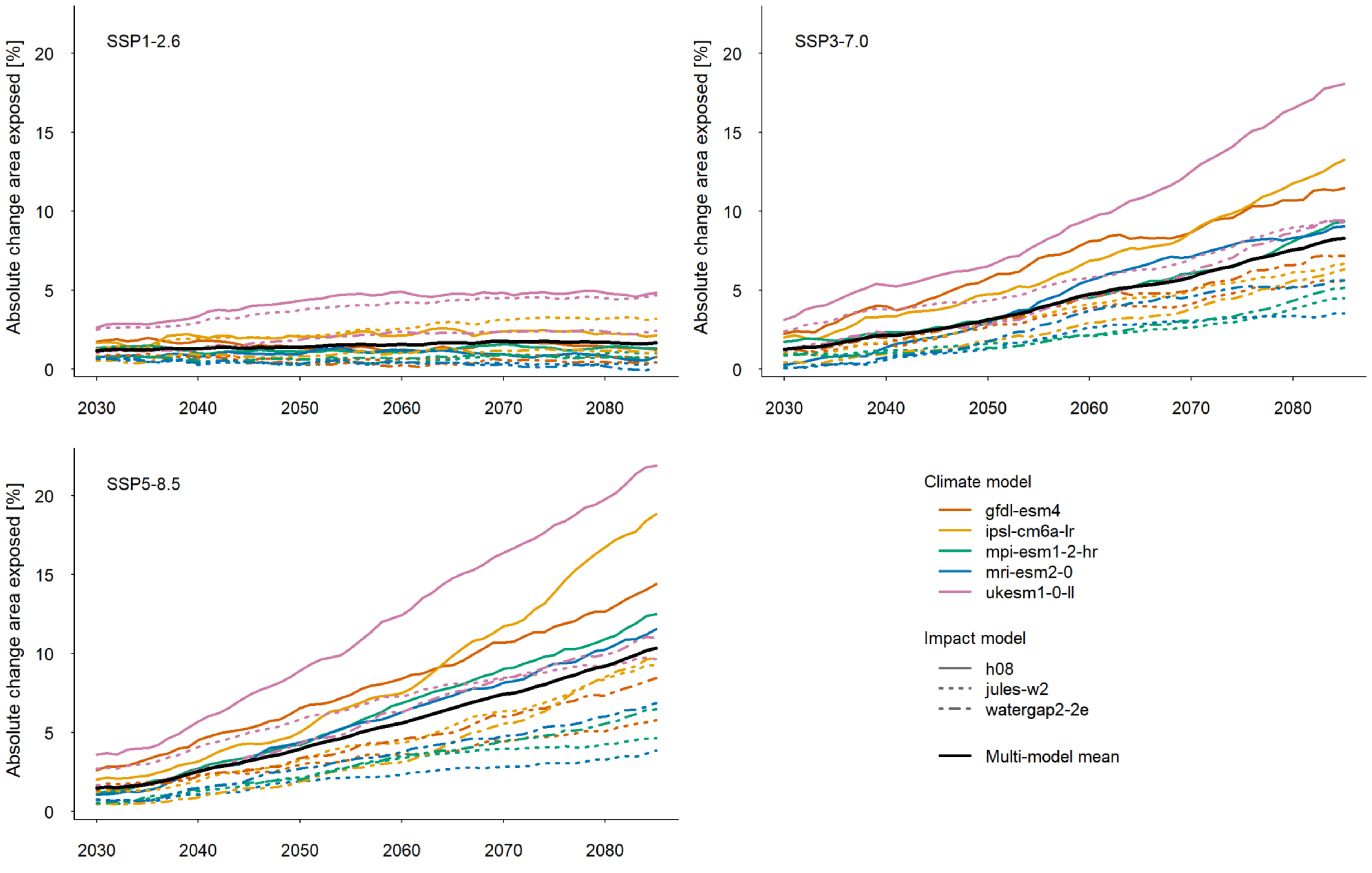

Supplementary Fig. 17c | Exposure of ecoregions to projected change in drought occurrences.

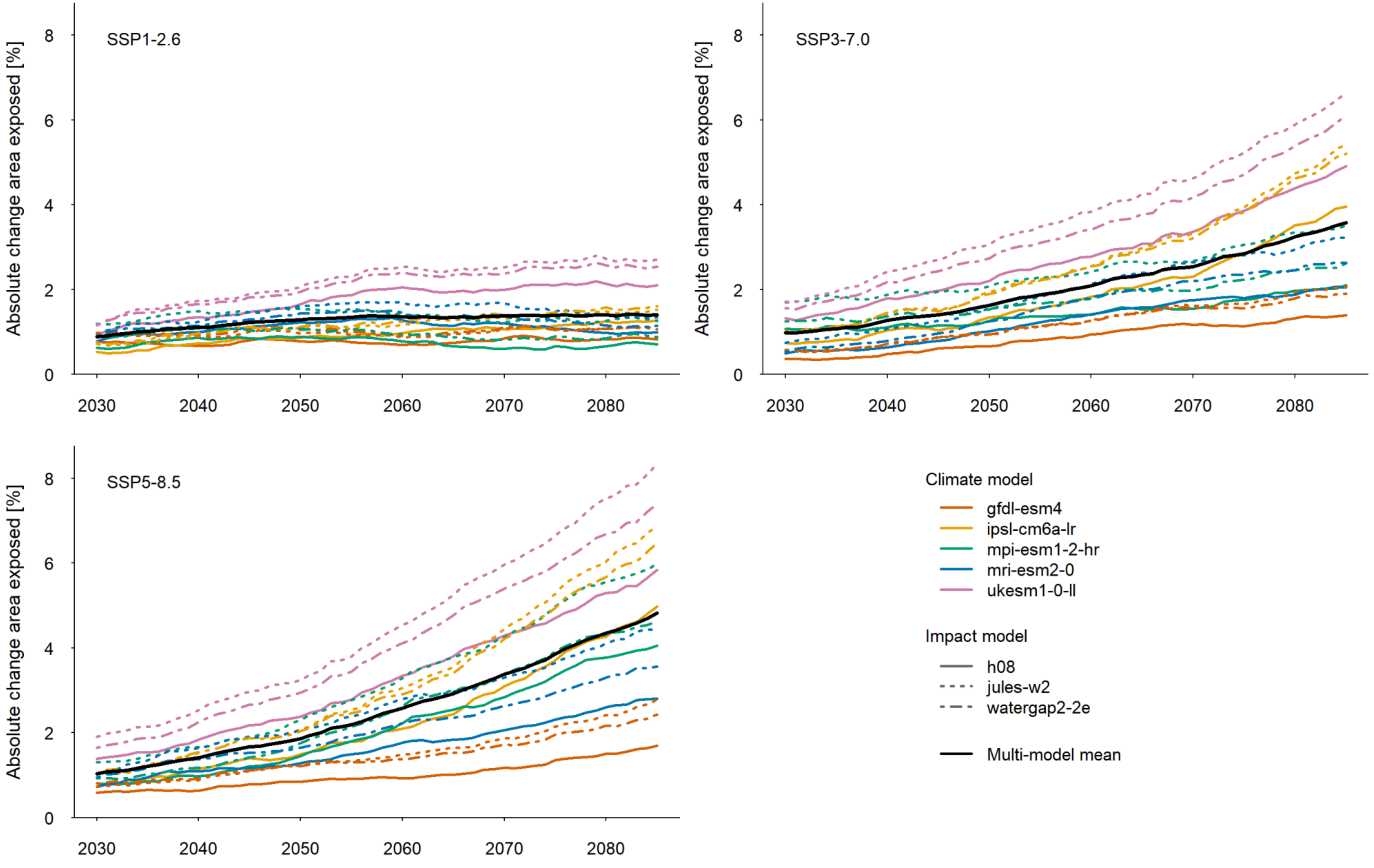

Supplementary Fig. 17d | Exposure of ecoregions to projected change in river flood occurrences.

References

Abraham J O, Hempson G P and Staver A C 2019 Drought-response strategies of savanna herbivores *Ecology and Evolution* **9** 7047–56

Ancillotto L, Bosso L, Conti P and Russo D 2021 Resilient responses by bats to a severe wildfire: conservation implications *Animal Conservation* **24** 470–81

Ashraf A, Darzi M M, Wani B M, Shah S A, Shabir M and Shafi M 2017 Climate change and infectious diseases of animals: A review *Journal of Entomology and Zoology Studies* **5** 1470–7

Barbaree B A, Reiter M E, Hickey C M, Strum K M, Isola J E, Jennings S, Tarjan L M, Strong C M, Stenzel L E and Shuford W D 2020 Effects of drought on the abundance and distribution of non-breeding shorebirds in central California, USA *PLOS ONE* **15** e0240931

Barrile G M, Chalfoun A D, Estes-Zumpf W A and Walters A W 2022 Wildfire influences individual growth and breeding dispersal, but not survival and recruitment in a montane amphibian *Ecosphere* **13** e4212

Beranek C T, Hamer A J, Mahony S V, Stauber A, Ryan S A, Gould J, Wallace S, Stock S, Kelly O, Parkin T, Weigner R, Daly G, Callen A, Rowley J J L, Klop-Toker K and Mahony M 2023 Severe wildfires promoted by climate change negatively impact forest amphibian metacommunities *Diversity and Distributions* **29** 785–800

Best M J, Pryor M, Clark D B, Rooney G G, Essery R L H, Ménard C B, Edwards J M, Hendry M A, Porson A, Gedney N, Mercado L M, Sitch S, Blyth E, Boucher O, Cox P M, Grimmond C S B and Harding R J 2011 The Joint UK Land Environment Simulator (JULES), model description – Part 1: Energy and water fluxes *Geoscientific Model Development* **4** 677–99

Beukema W, Pasmans F, Van Praet S, Ferri-Yáñez F, Kelly M, Laking A E, Erens J, Speybroeck J, Verheyen K, Lens L and Martel A 2021 Microclimate limits thermal behaviour favourable to disease control in a nocturnal amphibian *Ecology Letters* **24** 27–37

Blaustein A R, Walls S C, Bancroft B A, Lawler J J, Searle C L and Gervasi S S 2010 Direct and Indirect Effects of Climate Change on Amphibian Populations *Diversity* **2** 281–313

Bodmer R, Mayor P, Antunez M, Chota K, Fang T, Puertas P, Pittet M, Kirkland M, Walkey M, Rios C, Perez-Peña P, Henderson P, Bodmer W, Bicerra A, Zegarra J and Docherty E 2018 Major shifts in Amazon wildlife populations from recent intensification of floods and drought *Conservation Biology* **32** 333–44

Breitenbach A T, Carter A W, Paitz R T and Bowden R M 2020 Using naturalistic incubation temperatures to demonstrate how variation in the timing and continuity of heat wave exposure influences phenotype *Proceedings of the Royal Society B: Biological Sciences* **287** 20200992

Cayuela H, Arsovski D, Bonnaire E, Duguet R, Joly P and Besnard A 2016 The impact of severe drought on survival, fecundity, and population persistence in an endangered amphibian *Ecosphere* **7** e01246

Chowdhury M A W, Varela S, Roy S, Rahman M M, Noman M, Haidar I K A and Müller J 2022 Favourable climatic niche in low elevations outside the flood zone characterises the distribution pattern of venomous snakes in Bangladesh *Journal of Tropical Ecology* **38** 437–50

Crowley G M and Preece N D 2019 Does extreme flooding drive vegetation and faunal composition across the Gulf Plains of north-eastern Australia? *Austral Ecology* **44** 1256–70

Davis C L, Miller D A W, Walls S C, Barichivich W J, Riley J W and Brown M E 2017 Species interactions and the effects of climate variability on a wetland amphibian metacommunity *Ecological Applications* **27** 285–96

Dayananda B and Webb J K 2017 Incubation under climate warming affects learning ability and survival in hatchling lizards *Biology Letters* **13** 20170002

Dayrell J S, Fraga R de, Peres C A, Bobrowiec P E D, Magnusson W E and Lima A P 2024 Functional responses of amazonian frogs to flooding by a large hydroelectric dam *Biodivers Conserv* **33** 2055–70

Ding C, Newbold T and Ameca E I 2024 Assessing the global vulnerability of dryland birds to heatwaves *Global Change Biology* **30** e17136

Dobson R, Willis S G, Jennings S, Cheke R A, Challinor A J and Dallimer M 2024 Near-Term Forecasting of Terrestrial Mobile Species Distributions for Adaptive Management Under Extreme Weather Events *Global Change Biology* **30** e17579

Dundas S J, Ruthrof K X, Hardy G E St J and Fleming P A 2021 Some like it hot: Drought-induced forest die-off influences reptile assemblages *Acta Oecologica* **111** 103714

Elas M, Rosendal E and Meissner W 2023 The Effect of Floods on Nest Survival Probability of Common Sandpiper Actitis hypoleucos Breeding in the Riverbed of a Large Lowland European River *Diversity* **15** 90

Funghi C, McCowan L S C, Schuett W and Griffith S C 2019 High air temperatures induce temporal, spatial and social changes in the foraging behaviour of wild zebra finches *Animal Behaviour* **149** 33–43

Gandiwa E, Heitkönig I M A, Eilers P H C and Prins H H T 2016 Rainfall variability and its impact on large mammal populations in a complex of semi-arid African savanna protected areas *Tropical Ecology* **57** 163–80

Grimm-Seyfarth A, Mihoub J-B, Gruber B and Henle K 2018 Some like it hot: from individual to population responses of an arboreal arid-zone gecko to local and distant climate *Ecological Monographs* **88** 336–52

Hall J M and Warner D A 2018 Thermal spikes from the urban heat island increase mortality and alter physiology of lizard embryos *Journal of Experimental Biology* **221** jeb181552

Hanasaki N, Yoshikawa S, Pokhrel Y and Kanae S 2018 A global hydrological simulation to specify the sources of water used by humans *Hydrology and Earth System Sciences* **22** 789–817

Hossack B R and Pilliod D S 2011 Amphibian Responses to Wildfire in the Western United States: Emerging Patterns from Short-Term Studies *fire ecol* **7** 129–44

Hurley L L, McDiarmid C S, Friesen C R, Griffith S C and Rowe M 2018 Experimental heatwaves negatively impact sperm quality in the zebra finch *Proceedings of the Royal Society B: Biological Sciences* **285** 20172547

Ito A 2019 Disequilibrium of terrestrial ecosystem CO_2 budget caused by disturbance-induced emissions and non-CO_2 carbon export flows: a global model assessment *Earth System Dynamics* **10** 685–709

Linley G D, Jolly C J, Wooster E I F, Spencer E E, Cowan M A, Geary W L, de Laive A, Michael D R, Ritchie E G and Nimmo D G 2024 Widespread resilience of animal species, functional diversity, and predator–prey networks to an unprecedented gigafire *Journal of Applied Ecology* **61** 2959–70

Low E, Florko K, Mahoney H and Barclay R 2024 Effects of a severe wildfire on a bat community in the Canadian Rocky Mountains *Forest Ecology and Management* **563** 121983

Macdonald K J, Driscoll D A, Macdonald K J, Hradsky B and Doherty T S 2023 Meta-analysis reveals impacts of disturbance on reptile and amphibian body condition *Global Change Biology* **29** 4949–65

Martín J, Ortega J, García-Roa R, Rodríguez-Ruiz G, Pérez-Cembranos A and Pérez-Mellado V 2023 Coping with drought? Effects of extended drought conditions on soil invertebrate prey and diet selection by a fossorial amphisbaenian reptile *Current Zoology* **69** 367–76

Maxwell S L, Butt N, Maron M, McAlpine C A, Chapman S, Ullmann A, Segan D B and Watson J E M 2019 Conservation implications of ecological responses to extreme weather and climate events *Diversity and Distributions* **25** 613–25

McDevitt-Galles T, Moss W E, Calhoun D M, Briggs C J and Johnson P T J 2022 How extreme drought events, introduced species, and disease interact to influence threatened amphibian populations *Freshwater Science* Online: https://www.journals.uchicago.edu/doi/10.1086/722679

McDonald L A, Grayson K L, Lin H A and Vonesh J R 2018 Stage-specific effects of fire: Effects of prescribed burning on adult abundance, oviposition habitat selection, and larval performance of Cope's Gray Treefrog (*Hyla chrysoscelis*) *Forest Ecology and Management* **430** 394–402

Melton J R, Arora V K, Wisernig-Cojoc E, Seiler C, Fortier M, Chan E and Teckentrup L 2020 CLASSIC v1.0: the open-source community successor to the Canadian Land Surface Scheme (CLASS) and the Canadian Terrestrial Ecosystem Model (CTEM) – Part 1: Model framework and site-level performance *Geoscientific Model Development* **13** 2825–50

Mo M, Roache M, Davies J, Hopper J, Pitty H, Foster N, Guy S, Parry-Jones K, Francis G, Koosmen A, Colefax L, Costello C, Stokes J, Curran S, Smith M, Daly G, Simmons C-M, Hansen R, Prophet D, Judge S, Major F, Hogarth T, McGarry C-A, Pope L, Brend S, Coxon D, Baker K, Kaye K, Collins L, Wallis M, Brown R, Roberts L, Taylor S, Pearson T, Bishop T, Dunne P, Coutts-McClelland K, Oliver L, Dawe C, Welbergen J A, Mo M, Roache M, Davies J, Hopper J, Pitty H, Foster N, Guy S, Parry-Jones K, Francis G, Koosmen A, Colefax L, Costello C, Stokes J, Curran S, Smith M, Daly G, Simmons C-M, Hansen R, Prophet D, Judge S, Major F, Hogarth T, McGarry C-A, Pope L, Brend S, Coxon D, Baker K, Kaye K, Collins L, Wallis M, Brown R, Roberts L, Taylor S, Pearson T, Bishop T, Dunne P, Coutts-McClelland K, Oliver L, Dawe C and Welbergen J A 2021 Estimating flying-fox mortality associated with abandonments of pups and extreme heat events during the austral summer of 2019–20 *Pac. Conserv. Biol.* **28** 124–39

Morandini M, Mazzamuto M V and Koprowski J L 2023 Foraging Behavior Response of Small Mammals to Different Burn Severities *Fire* **6** 367

Müller Schmied H, Cáceres D, Eisner S, Flörke M, Herbert C, Niemann C, Peiris T A, Popat E, Portmann F T, Reinecke R, Schumacher M, Shadkam S, Telteu C-E, Trautmann T and Döll P 2021 The global water resources and use model WaterGAP v2.2d: model description and evaluation *Geoscientific Model Development* **14** 1037–79

Müller Schmied H, Trautmann T, Ackermann S, Cáceres D, Flörke M, Gerdener H, Kynast E, Peiris T A, Schiebener L, Schumacher M and Döll P 2024 The global water resources and use model WaterGAP v2.2e: description and evaluation of modifications and new features *Geoscientific Model Development* **17** 8817–52

Nelson S B M, Ribic C A, Niemuth N D, Bernath-Plaisted J and Zuckerberg B 2024 Sensitivity of North American grassland birds to weather and climate variability *Conservation Biology* **38** e14143

Nihei A, Sanderfoot O V, LaBarbera K and Tingley M W 2024 Wildfire smoke impacts the body condition and capture rates of birds in California *Ornithology* ukae023

Oberhagemann L, Billing M, von Bloh W, Drüke M, Forrest M, Bowring S P K, Hetzer J, Ribalaygua Batalla J and Thonicke K 2024 Sources of Uncertainty in the Global Fire Model SPITFIRE: Development of LPJmL-SPITFIRE1.9 and Directions for Future Improvements *EGUsphere* 1–44

Overton C T, Lorenz A A, James E P, Ahmadov R, Eadie J M, Mcduie F, Petrie M J, Nicolai C A, Weaver M L, Skalos D A, Skalos S M, Mott A L, Mackell D A, Kennedy A, Matchett E L and Casazza M L 2022 Megafires and thick smoke portend big problems for migratory birds *Ecology* **103** e03552

Prugh L R, Deguines N, Grinath J B, Suding K N, Bean W T, Stafford R and Brashares J S 2018 Ecological winners and losers of extreme drought in California *Nature Clim Change* **8** 819–24

Regan C E and Sheldon B C 2023 Phenotypic plasticity increases exposure to extreme climatic events that reduce individual fitness *Global Change Biology* **29** 2968–80

Rogers K H, Girard Y A, Woods L W and Johnson C K 2018 Avian trichomonosis mortality events in band-tailed pigeons (*Patagioenas fasciata*) in California during winter 2014–2015 *International Journal for Parasitology: Parasites and Wildlife* **7** 261–7

Rollins-Smith L A and Le Sage E H 2023 Heat stress and amphibian immunity in a time of climate change *Philosophical Transactions of the Royal Society B: Biological Sciences* **378** 20220132

Santos J L, Sitters H, Keith D A, Geary W L, Tingley R and Kelly L T 2022 A demographic framework for understanding fire-driven reptile declines in the 'land of the lizards' *Global Ecology and Biogeography* **31** 2105–19

Santos X, Chergui B, Belliure J, Moreira F and Pausas J G 2025 Reptile responses to fire across the western Mediterranean Basin *Conservation Biology* **39** e14326

Scheele B C, Driscoll D A, Fischer J and Hunter D A 2012 Decline of an endangered amphibian during an extreme climatic event *Ecosphere* **3** art101

Sharpe L, Cale B and Gardner J L 2019 Weighing the cost: the impact of serial heatwaves on body mass in a small Australian passerine *Journal of Avian Biology* **50** Online: https://onlinelibrary.wiley.com/doi/abs/10.1111/jav.02355

Smith C F, Schuett G W, Reiserer R S, Dana C E, Collyer M L and Davis M A 2019 Drought-induced Suppression of Female Fecundity in a Capital Breeder *Sci Rep* **9** 15499

Sokos C, Birtsas P, Papaspyropoulos K G, Tsachalidis E, Giannakopoulos A, Milis C, Spyrou V, Manolakou K, Valiakos G, Iakovakis C, Athanasiou L V, Sfougaris A and Billinis C 2016 Mammals and habitat disturbance: the case of brown hare and wildfire *Current Zoology* **62** 421–30

Sperry J H and Weatherhead P J 2008 Prey-Mediated Effects of Drought on Condition and Survival of a Terrestrial Snake *Ecology* **89** 2770–6

Stiegler J, Pahl J, Guillen R A, Ullmann W and Blaum N 2023 The heat is on: impacts of rising temperature on the activity of a common European mammal *Front. Ecol. Evol.* **11** Online: https://www.frontiersin.org/journals/ecology-and-evolution/articles/10.3389/fevo.2023.1193861/full

Stillman A N, Siegel R B, Wilkerson R L, Johnson M, Howell C A and Tingley M W 2019 Nest site selection and nest survival of Black-backed Woodpeckers after wildfire *The Condor* **121** duz039

Stojanovic D, Webb nee Voogdt J, Webb M, Cook H and Heinsohn R 2016 Loss of habitat for a secondary cavity nesting bird after wildfire *Forest Ecology and Management* **360** 235–41

Tomas W M, Berlinck C N, Chiaravalloti R M, Faggioni G P, Strüssmann C, Libonati R, Abrahão C R, do Valle Alvarenga G, de Faria Bacellar A E, de Queiroz Batista F R, Bornato T S, Camilo A R, Castedo J, Fernando A M E, de Freitas G O, Garcia C M, Gonçalves H S, de Freitas Guilherme M B, Layme V M G, Lustosa A P G, De Oliveira A C, da Rosa Oliveira M, de Matos Martins Pereira A, Rodrigues J A, Semedo T B F, de Souza R A D, Tortato F R, Viana D F P, Vicente-Silva L and Morato R 2021 Distance sampling surveys reveal 17 million vertebrates directly killed by the 2020's wildfires in the Pantanal, Brazil *Sci Rep* **11** 23547

Trondrud L M, Pigeon G, Król E, Albon S, Ropstad E, Kumpula J, Evans A L, Speakman J R and Loe L E 2023 A Summer Heat Wave Reduced Activity, Heart Rate, and Autumn Body Mass in a Cold-Adapted Ungulate *Physiological and Biochemical Zoology* **96** 282–93

Ujvari B, Brown G, Shine R and Madsen T 2016 Floods and famine: climate-induced collapse of a tropical predator-prey community *Functional Ecology* **30** 453–8

Watter K, Baxter G, Brennan M, Pople A and Murray P 2019 Decline in body condition and high drought mortality limit the spread of wild chital deer in north-east Queensland, Australia *Rangel. J.* **41** 293–9

Weerathunga W A M T and Rajapaksa G 2020 The impact of elevated temperature and CO2 on growth, physiological and immune responses of Polypedates cruciger (common hourglass tree frog) *Frontiers in Zoology* **17** 3

Wells C P, Barbier R, Nelson S, Kanaziz R and Aubry L M 2022 Life history consequences of climate change in hibernating mammals: a review *Ecography* **2022** e06056

Westphal M F, Stewart J A E, Tennant E N, Butterfield H S and Sinervo B 2016 Contemporary Drought and Future Effects of Climate Change on the Endangered Blunt-Nosed Leopard Lizard, Gambelia sila *PLOS ONE* **11** e0154838

Wilson J and Peach W 2006 Impact of an exceptional winter flood on the population dynamics of bearded tits (Panurus biarmicus) *Animal Conservation* **9** 463–73

Wirth S B, Braun J, Heinke J, Ostberg S, Rolinski S, Schaphoff S, Stenzel F, von Bloh W, Taube F and Müller C 2024 Biological nitrogen fixation of natural and agricultural vegetation simulated with LPJmL 5.7.9 *Geoscientific Model Development* **17** 7889–914

Zhang Q, Han X-Z, Burraco P, Wang X-F, Teng L-W, Liu Z-S and Du W-G 2023 Oxidative stress mediates the impact of heatwaves on survival, growth and immune status in a lizard *Proceedings of the Royal Society B: Biological Sciences* **290** 20231768
