# Supplementary material for: Land vertebrates increasingly exposed to multiple extreme events by 2085
Source: Nat Ecol Evol. 2026 Apr 24;10(5):854–63. doi: 10.1038/s41559-026-03050-0 (PMC13167462; doi:10.1038/s41559-026-03050-0)
Supplement: Supplementary file 2 — Reporting Summary [file 41559_2026_3050_MOESM2_ESM.pdf]

nature portfolio

Corresponding author(s): Stefanie Heinicke

Last updated by author(s): Mar 4, 2026

Reporting Summary

Nature Portfolio wishes to improve the reproducibility of the work that we publish. This form provides structure for consistency and transparency in reporting. For further information on Nature Portfolio policies, see our Editorial Policies and the Editorial Policy Checklist.

Statistics

For all statistical analyses, confirm that the following items are present in the figure legend, table legend, main text, or Methods section.

n/a	Confirmed	
☐	☒	The exact sample size (*n*) for each experimental group/condition, given as a discrete number and unit of measurement
☒	☐	A statement on whether measurements were taken from distinct samples or whether the same sample was measured repeatedly
☒	☐	The statistical test(s) used AND whether they are one- or two-sided<br>*Only common tests should be described solely by name; describe more complex techniques in the Methods section.*
☒	☐	A description of all covariates tested
☒	☐	A description of any assumptions or corrections, such as tests of normality and adjustment for multiple comparisons
☐	☒	A full description of the statistical parameters including central tendency (e.g. means) or other basic estimates (e.g. regression coefficient) AND variation (e.g. standard deviation) or associated estimates of uncertainty (e.g. confidence intervals)
☒	☐	For null hypothesis testing, the test statistic (e.g. *F*, *t*, *r*) with confidence intervals, effect sizes, degrees of freedom and *P* value noted<br>*Give P values as exact values whenever suitable.*
☒	☐	For Bayesian analysis, information on the choice of priors and Markov chain Monte Carlo settings
☒	☐	For hierarchical and complex designs, identification of the appropriate level for tests and full reporting of outcomes
☒	☐	Estimates of effect sizes (e.g. Cohen's *d*, Pearson's *r*), indicating how they were calculated

Our web collection on statistics for biologists contains articles on many of the points above.

Software and code

Policy information about availability of computer code

Data collection	No software or computer code were used for data collection.
Data analysis	Data were processed and analysed in R version 4.1.0 (2021-05-18) using the following packages: ncdf4 vers 1.19, sf vers 1.0.8, sp vers 1.5.0.<br>Figures were created in R vers 4.1.0 using the packages classInt vers 0.4.10, RColorBrewer vers 1.1.3, viridis vers 0.6.4<br>Custom code developed for this study is deposited on Zenodo: https://doi.org/10.5281/zenodo.18861352.

For manuscripts utilizing custom algorithms or software that are central to the research but not yet described in published literature, software must be made available to editors and reviewers. We strongly encourage code deposition in a community repository (e.g. GitHub). See the Nature Portfolio guidelines for submitting code & software for further information.

Data

Policy information about availability of data

All manuscripts must include a data availability statement. This statement should provide the following information, where applicable:

- Accession codes, unique identifiers, or web links for publicly available datasets
- A description of any restrictions on data availability
- For clinical datasets or third party data, please ensure that the statement adheres to our policy

Climate projections, climate impact projections and extreme event data are available from the ISIMIP Repository (https://data.isimip.org/), specifically heatwave and wildfire data can be downloaded from https://doi.org/10.48364/ISIMIP.920810, and drought and river flood data from https://doi.org/10.48364/

ISIMIP.792444. Species richness layers were downloaded from the IUCN Red List of Threatened Species (https://www.iucnredlist.org/). Species range data were downloaded from the IUCN Red List of Threatened Species (for terrestrial mammals and amphibians), from the Global Assessment of Reptile Distributions (GARD, http://www.gardinitiative.org/), and BirdLife International (http://datazone.birdlife.org). Ecoregions were downloaded from https://ecoregions.appspot.com/.

Research involving human participants, their data, or biological material

Policy information about studies with human participants or human data. See also policy information about sex, gender (identity/presentation), and sexual orientation and race, ethnicity and racism.

Reporting on sex and gender	N/A
Reporting on race, ethnicity, or other socially relevant groupings	N/A
Population characteristics	N/A
Recruitment	N/A
Ethics oversight	N/A

Note that full information on the approval of the study protocol must also be provided in the manuscript.

Field-specific reporting

Please select the one below that is the best fit for your research. If you are not sure, read the appropriate sections before making your selection.

☐ Life sciences ☐ Behavioural & social sciences ☒ Ecological, evolutionary & environmental sciences

For a reference copy of the document with all sections, see nature.com/documents/nr-reporting-summary-flat.pdf

Ecological, evolutionary & environmental sciences study design

All studies must disclose on these points even when the disclosure is negative.

Study description	The study analysed the exposure of current terrestrial vertebrate geographic ranges and ecoregions to extreme events (drought, heatwave, river flood, wildfires) based on climate model and climate impact model projections.
Research sample	Species richness data (all species and threatened species for terrestrial vertebrates), rarity-weighted richness data (terrestrial vertebrates), species geographic range data for terrestrial vertebrates and ecoregion data; and annual extreme event data for four types of extreme events (drought, heatwave, river flood, wildfire) based on six climate impact models forced by climate projections from five CMIP6 climate models under three emission scenarios (SSP1-2.6, SSP3-7.0, SSP5-8.5).
Sampling strategy	The initial dataset of geographic range data comprised 7,731 amphibians, 10,992 birds, 5,593 mammals, and 10,914 reptiles. We mapped species ranges to the ISIMIP grid, by calculating for each grid cell the proportion of overlap with the species range. Due to the spatial resolution, small islands are not included in the ISIMIP grid and extreme event data are not available. Species restricted to small islands not represented in the ISIMIP grid were excluded (126 amphibians, 430 birds, 117 mammals, 621 reptiles), resulting in a final dataset of 33,936 terrestrial vertebrate species (7,605 amphibians, 10,562 birds, 5,476 mammals, 10,293 reptiles).<br>The ecoregion dataset includes 847 ecoregions, but 53 ecoregions had no overlap with ISIMIP land cells. We used the remaining 794 ecoregions for our analysis.
Data collection	To identify hotspots of terrestrial biodiversity exposure, we used species richness layers from the IUCN Red List of Threatened Species (version 2023-1, accessed August 2024) for amphibians, birds, mammals, and reptiles. For each taxon, we used three types of layers: number of species per grid cell (species richness, Fig. 1a-d, Extended Data Fig. 1d-f), number of threatened species (Supplementary Fig. 1), and rarity-weighted richness (aggregate importance of each grid cell to the species occurring there, unitless, Supplementary Fig. 2).<br>To quantify exposure for individual species, we obtained species range data from the IUCN Red List of Threatened Species (terrestrial mammals and amphibians), the Global Assessment of Reptile Distributions (GARD, reptiles), and BirdLife International (birds).<br>Ecoregion outlines were obtained from the Ecoregions 2017 dataset (Dinerstein et al. 2017).<br>Climate and climate impact projections were obtained from the ISIMIP Repository.
Timing and spatial scale	spatial scale is global, pre-industrial control simulation was used for 1850-2100, historical simulation was used for 1985-2014, future simulation for 2015-2100
Data exclusions	No data was excluded.
Reproducibility	The analysis is fully reproducible. Biological data are from public sources, extreme event data and code is publicly available.
Randomization	N/A

Blinding | N/A

Did the study involve field work? ☐ Yes ☒ No

Reporting for specific materials, systems and methods

We require information from authors about some types of materials, experimental systems and methods used in many studies. Here, indicate whether each material, system or method listed is relevant to your study. If you are not sure if a list item applies to your research, read the appropriate section before selecting a response.

Materials & experimental systems		
n/a	Involved in the study	
☒	☐	Antibodies
☒	☐	Eukaryotic cell lines
☒	☐	Palaeontology and archaeology
☒	☐	Animals and other organisms
☒	☐	Clinical data
☒	☐	Dual use research of concern
☒	☐	Plants

Methods		
n/a	Involved in the study	
☒	☐	ChIP-seq
☒	☐	Flow cytometry
☒	☐	MRI-based neuroimaging

Plants

Seed stocks | N/A

Novel plant genotypes | N/A

Authentication | N/A
